# Integrated Genomic and Single‐Cell Analysis Reveals Heterogeneity, Prognosis, and Treatment Vulnerability in Urothelial Carcinoma

**DOI:** 10.1155/humu/2797474

**Published:** 2026-05-27

**Authors:** Chaozhi Tang, Yifan Liu, Tian-Long Wang, Jiakang Ma, Weihua Chen, Xiang Liu, Jingdong Xue, Lin Ye, Feng Yue

**Affiliations:** ^1^ Department of Urology, Shanghai East Hospital, School of Medicine, Tongji University, Shanghai, China, tongji.edu.cn; ^2^ Department of Urology, Tongji Hospital, School of Medicine, Tongji University, Shanghai, China, tongji.edu.cn; ^3^ Department of Urology, Shanghai Eighth People′s Hospital, Shanghai, China; ^4^ The First Affiliated Hospital, College of Clinical Medicine of Henan University of Science and Technology, Shanghai, China, haust.edu.cn; ^5^ Department of Urology, Putuo People′s Hospital, School of Medicine, Tongji University, Shanghai, China, tongji.edu.cn; ^6^ Department of Urology, The Affiliated Huizhou Hospital, Guangzhou Medical University, Huizhou, Guangdong, China, gzhmc.edu.cn

**Keywords:** immunotherapy response, prognostic assessment, signature subtypes, urothelial carcinoma

## Abstract

At the transcriptomic level, several molecular subtyping schemes have been established to elucidate the intrinsic heterogeneity of urothelial carcinoma and to inform prognostic assessment and therapeutic guidance. However, a unified molecular classification scheme characterizing genomic alterations is lacking. Unsupervised and supervised clustering identified two distinct mutational signature subtypes. Kaplan–Meier analysis demonstrated that patients with the MUT2 subtype had a higher risk of death than those with the MUT1 subtype across multiple cohorts, including IMvigor210 (hazard ratio [HR], 1.74; 95% confidence interval [CI], 1.27–2.37; *p* < 0.001), UC‐GENOME (HR, 1.54; 95% CI, 0.93–2.54; *p* = 0.091), The Cancer Genome Atlas (TCGA; HR, 1.45; 95% CI, 1.06–1.98; *p* = 0.020), MSK2022 (HR, 1.34; 95% CI, 1.10–1.64; *p* = 0.004), MSK2015 (HR, 3.43; 95% CI, 1.36–8.64; *p* = 0.005), and the Tongji cohort (HR, 4.99; 95% CI, 0.57–43.69; *p* = 0.11). Immunotherapy response rates were significantly higher in the MUT1 subtype than in the MUT2 subtype in IMvigor210 (31.8% vs. 13.1%; *p* = 0.003) and UC‐GENOME (42.3% vs. 29.0%; *p* = 0.022). Consistent with these findings, single‐cell analysis showed that MUT2 tumors were enriched in tumor‐associated fibroblast subpopulations and had a lower abundance of immune effector cells. Overall, this genomic analysis identified two mutation‐based subtypes of urothelial carcinoma associated with patient prognosis and immunotherapy response.

## 1. Introduction

Bladder cancer (BC) is a prevalent and lethal malignancy worldwide [1], and its development is associated with risk factors, such as smoking, advancing age, male sex, and certain occupational exposures [2–5]. Although therapeutic options continue to evolve, the mainstays of treatment include surgery, chemotherapy, and immunotherapy [6]. Nevertheless, patient outcomes vary markedly between individuals; tumor molecular characteristics and mutational profiles are thought to contribute to prognosis and treatment response [7, 8]. In recent years, molecular subtyping of BC has emerged as a critical factor in understanding the disease biology and developing personalized treatment strategies [9–11]. For instance, Choi et al. [12] used gene expression profiling to classify muscle‐invasive BC into luminal, basal, and p53‐like subtypes. The basal subtype is characterized by squamous differentiation and greater aggressiveness and is associated with shorter survival, whereas the p53‐like subtype exhibits significant resistance to chemotherapy. Building on these findings, Kamoun et al. [13] leveraged large‐scale transcriptomic data to establish the first consensus molecular classification of muscle‐invasive BC, which aids in patient risk stratification but does not incorporate treatment‐related data.

Studies have indicated that mutations in genes such as *FGFR3*, *TP53, MDM2*, *ERCC2*, *PIK3CA*, and *RB1* are associated with tumor aggressiveness, metastatic potential, and responses to chemotherapy and immunotherapy [14–25]. In upper tract urothelial carcinoma (UC), Fujii et al. [26] delineated five subtypes based on the mutation status of *TP53*, *MDM2*, *RAS*, and *FGFR3*, revealing distinct molecular and prognostic features. Nevertheless, single‐gene alterations alone are insufficient to capture the complexity of the cancer genomic landscape or fully explain tumor biology and therapeutic responses [27]. Mutational signatures—characteristic combinations of mutation types resulting from specific mutagenic processes—reflect the biological mechanisms or external influences to which a tumor has been exposed during its development [28, 29].

Compared with traditional bulk sequencing, single‐cell sequencing technologies (e.g., scRNA‐seq and scATAC‐seq) and spatial transcriptomics enable the investigation of tumor heterogeneity and dynamic microenvironmental changes at single‐cell resolution [30, 31]. These approaches overcome the limitations of signal averaging inherent in bulk methods, thereby uncovering key cellular subsets—such as cancer stem cells—and tracking their phenotypic evolution during disease progression or in response to therapy [32, 33]. Such insights are particularly valuable for elucidating mechanisms of drug resistance, as single‐cell profiling can identify adaptive cellular states and their molecular signatures within resistant tumors, revealing how transcriptional reprogramming or microenvironmental interactions contribute to therapeutic escape [33–35]. Collectively, single‐cell and spatial technologies provide an unprecedented level of resolution in cancer biology, enabling the characterization of cellular heterogeneity, reconstruction of dynamic regulatory networks, and identification of novel therapeutic targets.

Using an unsupervised mutational signature–based clustering approach, we identified two stable molecular subtypes of UC. In this study, we identified intrinsic mutational subtypes of UC and investigated, at single‐cell resolution, the differential tumor microenvironment associated with these subtypes. We further explored their therapeutic vulnerabilities, particularly in the context of immunotherapy. In addition, we evaluated the robustness and stability of this classification model using machine learning approaches.

## 2. Materials and Methods

### 2.1. Data Source

Public genomic data from The Cancer Genome Atlas (TCGA) for primary BC [36], as well as immunotherapy cohorts from the IMvigor210 trial [37, 38] and Project UC‐GENOME [39] for metastatic UC of the bladder, were used in this study. Additionally, tumor and matched normal DNA libraries were sequenced using the Memorial Sloan Kettering–Integrated Mutation Profiling of Actionable Cancer Targets (MSK‐IMPACT) targeted sequencing platform in 2015 [40] and 2022 [41]; these datasets are referred to as the MSK2015 and MSK2022 cohorts, respectively. Whole‐exome sequencing (WES) data from patients with upper tract UC at Shanghai East Hospital (the Tongji cohort) were included as an independent, retrospectively sequenced cohort outside a clinical trial setting. This study was reviewed and approved by the Ethical Committee of Shanghai East Hospital (No. 2018YS‐04) and conducted in accordance with the Declaration of Helsinki. Written informed consent was obtained from all patients.

### 2.2. Molecular Profiling

DNA libraries were prepared according to Illumina‐recommended protocols. A total of 1 *μ*g of DNA was fragmented into approximately 200–300 bp fragments using a Covaris S220 ultrasonicator (Woburn, MA, United States). The DNA fragments were end‐repaired and ligated to adaptors. Purified products from 10 PCR cycles were incubated with capture probes at 65°C for 24 h for exome capture. The libraries were then subjected to massively parallel sequencing using an Xten system (Illumina, Shanghai, China). Trimmomatic [42] was used for data trimming, and the Burrows–Wheeler Aligner [43] was used to align reads to the human reference genome hg38. Variant calling was performed using SAMtools mpileup output pipelines [44], and variant annotation was conducted using ANNOVAR [45], GATK [46], and VEP [47].

### 2.3. Mutational Signatures and Subtyping

Mutation datasets obtained through either WES or targeted gene panel sequencing, comprising single‐nucleotide polymorphisms mapped to reference genomes, were analyzed using the R package *SomaticSignatures* [48] (R Version 4.0.1, R Foundation for Statistical Computing, Vienna, Austria) to identify mutational signatures from the Catalog of Somatic Mutations in Cancer (COSMIC) v3 database [49]. Cosine similarities (CSs) were calculated across 79 single‐base substitution (SBS) signatures (Supporting Information 1: Methods S1). TCGA cohort was used as the training set. Mutation signatures with CS < 0.24 present in fewer than 10% of the samples were excluded, resulting in 46 retained signatures. To determine the optimal classification, non‐negative matrix factorization was applied to the CS matrix of mutational signatures (Supporting Information 1: Methods S2, Supporting Information 2: Figure S1, Supporting Information 3: Figure S2, Supporting Information 4: Figure S3, Supporting Information 5: Figure S4, and Supporting Information 6: Figure S5). Two clusters were selected as the optimal solution based on the evaluation of clustering stability. A prediction analysis for a microarray classifier was subsequently trained on TCGA cohort and used to assign mutational subtypes to external cohorts using the R package *pamr*. Calculation of the risk score is described in Supporting Information 1: Methods S3, Supporting Information 7: Figure S6, and Supporting Information 17: Table S1. Briefly, “risk” refers to a mutation‐based prognostic score developed in the MSK2022 cohort and dichotomized at a survival‐optimized threshold of 1 (low risk < 1; high risk ≥ 1).

### 2.4. Analysis for Single‐Cell Sequencing Data

Single‐cell datasets GSE169379 (25 BC samples), PRJNA662018 (eight BC cases), and GSE211388 (10 human bladder tumors) were integrated. Seurat objects [50] were generated for each dataset with quality control filters requiring a minimum of 200 features and no cells with fewer than three detected genes. Cells with fewer than 200 or more than 5000 detected features (nFeature_RNA) or with > 20% mitochondrial gene content were excluded. The combined dataset was normalized using *NormalizeData* and scaled using *ScaleData*. Variable features were selected using the “vst” method, with the Top 2000 features retained. Principal component analysis (PCA) was performed, followed by batch‐effect correction using Harmony based on the “orig.ident” variable. Clustering was conducted on Harmony‐corrected data with a resolution parameter of 0.4. UMAP and t‐SNE were used for dimensionality reduction and visualization.

### 2.5. Gene Set Variation Analysis (GSVA)

GSVA is a nonparametric, unsupervised method that estimates the enrichment of predefined gene sets at the individual sample level, generating scores that reflect pathway or biological process activity [51]. In bulk tumor sequencing, GSVA enables the identification of pathway‐level expression patterns and biological signatures that may not be apparent from single‐gene analyses, facilitating the investigation of tumor heterogeneity and its associations with clinical outcomes.

### 2.6. Machine Learning Validation of Mutational Subtypes

Machine learning approaches, including Random Forest [52], LightGBM [53], XGBoost [54], CatBoost [55], AdaBoost [56], and logistic regression [57], were used to evaluate classification performance. TCGA cohort was randomly split into training (70%) and testing (30%) sets. External validation was performed using independent datasets such as IMvigor210 [37] to assess model robustness and generalizability (Supporting Information 1: Methods S4).

### 2.7. Statistical Analysis

Continuous variables were compared using the Wilcoxon rank‐sum test. Survival outcomes were evaluated using the log‐rank test, and categorical variables were compared using the chi‐square test. Analyses were performed using R (Version 4.0.1; R Foundation for Statistical Computing, Vienna, Austria), with *p* values < 0.05 considered statistically significant. Cox proportional hazard regression was used to estimate the risk of death between MUT1 and MUT2 subtypes, adjusting for age, sex, and smoking status. Survival analyses were performed using samples with available overall survival (OS) data, excluding cases with missing OS information.

## 3. Results

### 3.1. Demographic and Clinical Characteristics

Table 1 summarizes the clinical and genomic characteristics of patients in the IMvigor210 (*n* = 244), UC‐GENOME (*n* = 191), TCGA (*n* = 412), MSK2022 (*n* = 1278), MSK2015 (*n* = 83), and Tongji (*n* = 21) cohorts. Among the 2229 patients, 1689 (75.8%) were men, and 536 (24.1%) were women, with a mean age of 66.0 years (interquartile range [IQR], 59.0–74.0 years). In terms of smoking status, 294 (13.2%) patients were current smokers, 1157 (51.9%) were former smokers, and 709 (31.8%) were never smokers. A total of 871 (39.1%) OS events were observed. The mean follow‐up time was 31.4 months (IQR, 11.4–43.4 months).

**Table 1:** Demographic characteristics of the cohorts.

Characteristic	IMvigor210	UC‐GENOME	TCGA	MSK2022	MSK2015	Tongji
	(*n* = 244)	(*n* = 191)	(*n* = 412)	(*n* = 1278)	(*n* = 83)	(*n* = 21)
Age, mean (SD)	NA	65.8 (10.1)	68.1 (10.6)	65.2 (11.2)	67.7 (10.3)	67.8 (11.7)
Sex, no. (%)						
Female	50 (20.5)	47 (24.6)	108 (26.2)	296 (23.2)	28 (33.7)	7 (33.3)
Male	194 (79.5)	143 (74.9)	304 (73.8)	980 (76.7)	54 (65.1)	14 (66.7)
NA	0 (0)	1 (0.5)	0 (0)	2 (0.1)	1 (0.2)	0 (0)
Smoker, no. (%)						
Former	145 (59.4)	112 (58.6)	198 (48.1)	650 (50.9)	44 (53.0)	8 (38.1)
Never	74 (30.3)	62 (32.5)	111 (26.9)	433 (33.9)	22 (26.5)	7 (33.3)
Current	25 (10.3)	16 (8.4)	90 (21.8)	141 (11.0)	16 (19.3)	6 (28.6)
NA	0 (0)	1 (0.5)	13 (3.2)	54 (4.2)	1 (1.2)	0 (0)
Subtype, no. (%)						
Hypermutated	1 (0.4)	2 (1.0)	1 (0.2)	7 (0.5)	1 (1.2)	1 (4.8)
FGFR3	41 (16.8)	26 (13.6)	44 (10.7)	241 (18.9)	39 (47.0)	1 (4.8)
RAS	7 (2.9)	8 (4.2)	30 (7.3)	68 (5.3)	11 (13.2)	4 (19.0)
TP53/MDM2	99 (40.6)	105 (55.0)	199 (48.3)	629 (49.2)	14 (16.9)	6 (28.6)
Triple negative	96 (39.3)	50 (26.2)	138 (33.5)	333 (26.1)	18 (21.7)	9 (42.9)
TMB, no. (%)						
< 10 Muts/Mb	213 (87.3)	76 (39.8)	315 (76.5)	672 (52.6)	61 (73.5)	19 (90.5)
≥ 10 Muts/Mb	31 (12.7)	81 (42.4)	97 (23.5)	606 (47.4)	22 (26.5)	2 (9.5)
NA	0 (0)	34 (17.8)	0 (0)	0 (0)	0 (0)	0 (0)
Risk, no. (%)						
Low	121 (49.6)	97 (50.8)	173 (42.0)	608 (47.6)	46 (55.4)	9 (42.9)
High	123 (50.4)	94 (49.2)	239 (58.0)	670 (52.4)	37 (44.6)	12 (57.1)
Overall survival, no. (%)						
Event	159 (65.2)	78 (40.8)	158 (38.3)	445 (34.8)	25 (30.1)	6 (28.6)
Censored	85 (34.8)	85 (44.5)	250 (60.7)	793 (62.1)	43 (51.8)	15 (71.4)
NA	0 (0)	28 (14.7)	4 (1.0)	40 (3.1)	15 (18.1)	0 (0)
MS subtype, no. (%)						
MUT1	150 (61.5)	63 (33.0)	161 (39.1)	833 (65.2)	37 (44.6)	12 (57.1)
MUT2	94 (38.5)	128 (67.0)	251 (60.9)	445 (34.8)	46 (55.4)	9 (42.9)

*Note:* Risk, mutation‐based genomic risk score (Supporting Information 1: Methods S3): low risk, score < 1; high risk, score ≥ 1.

Abbreviations: NA, not available; SD, standard deviation; TCGA, The Cancer Genome Atlas; TMB, tumor mutation burden.

### 3.2. MUT2 Subtype Predicts Unfavorable Prognosis

**Figure 1:**
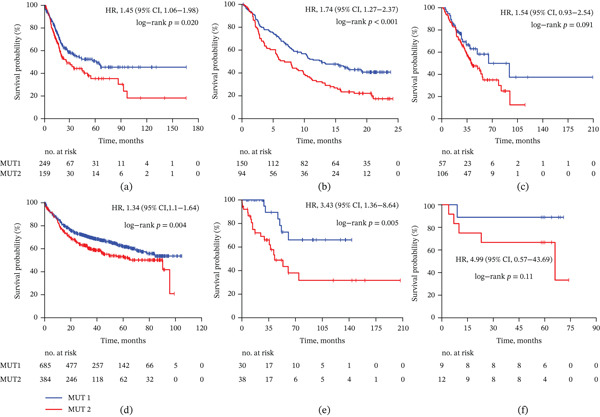
Survival outcomes according to the mutational subtype in all retrospective cohorts. Kaplan–Meier survival analysis results showing differences in overall survival for mutational signature subtypes in the (a) TCGA, (b) IMvigor210, (c) UC‐GENOME, (d) MSK2022, (e) MSK2015, and (f) Tongji self‐sequencing cohorts. Hazard ratios (HRs), 95% confidence intervals (CIs), and *p* values are indicated on each plot, along with the number of patients at risk.

Among the 244 patients with metastatic UC treated with immunotherapy, 150 (61.5%) were classified as MUT1 and 94 (38.5%) as MUT2 (Table 1). The median OS for patients with the MUT2 subtype was 7.5 months, which was shorter than both the overall cohort median (9.52 months) and the 15‐month median OS observed for the MUT1 subtype (hazard ratio [HR], 1.74; 95% confidence interval [CI], 1.27–2.37; *p* < 0.001) (Figure 1).

Consistent trends were observed across other cohorts, including UC‐GENOME (HR, 1.54; 95% CI, 0.93–2.54; *p* = 0.091) and TCGA (HR, 1.45; 95% CI, 1.06–1.98; *p* = 0.020). The association between MUT2 subtype and shorter survival was also evident in MSK2022 (HR, 1.34; 95% CI, 1.10–1.64; *p* = 0.004) and MSK2015 (HR, 3.43; 95% CI, 1.36–8.64; *p* = 0.005). Similarly, in the Tongji cohort, MUT2 was associated with poorer survival (HR, 4.99; 95% CI, 0.57–43.69; *p* = 0.11).

### 3.3. MUT1 Subtype Indicates Improved Immunotherapy Response

To explore the basis of survival differences between subtypes, we compared frequently mutated genes and clinical characteristics. No individual gene mutation showed a significant association with immunotherapy response (Supporting Information 8: Figure S7A). In contrast, mutational subtypes demonstrated clear differences: Patients with the MUT1 subtype had significantly higher response rates to immunotherapy than those with the MUT2 subtype in both IMvigor210 (31.8% vs. 13.1%; *χ* ^2^ test *p* = 0.003) and UC‐GENOME (42.3% vs. 29.0%; *χ* ^2^ test *p* = 0.022) (Supporting Information 8: Figure S7B). This disparity was also reflected in tumor immune phenotypes. MUT1 tumors showed a higher proportion of inflamed phenotypes compared with MUT2 in IMvigor210 (36.6% vs. 18.8%; *χ* ^2^ test *p* = 0.021) and UC‐GENOME (56.9% vs. 32.0%; *χ* ^2^ test *p* = 0.007) (Supporting Information 3: Figure S7C). Additionally, MUT1 tumors exhibited higher tumor mutational burden than MUT2 tumors (Supporting Information 8: Figure S7D,E and Supporting Information 17: Tables S2, S3, S4, S5, and S6), consistent with current understanding of immunotherapy responses in UC.

Subgroup analyses further supported these findings. Among patients with an Eastern Cooperative Oncology Group (ECOG) performance status of 1, MUT2 was associated with a higher risk of death than MUT1 (IMvigor210: HR, 2.47; 95% CI, 1.65–3.70; *p* < 0.001; UC‐GENOME: HR, 2.96; 95% CI, 1.03–8.46; *p* = 0.04) (Supporting Information 9: Figure S8 and Supporting Information 10: Figure S9). Similarly, in patients who received platinum chemotherapy (IMvigor210: HR, 2.02; 95% CI, 1.41–2.90; *p* < 0.001) or derived a clinical benefit from chemotherapy (UC‐GENOME: HR, 8.73; 95% CI, 1.07–71.43; *p* = 0.04), the MUT2 subtype conferred a higher risk of death than the MUT1 subtype. This increased risk for the MUT2 subtype (vs. the MUT1 subtype) was also observed in the subgroup of former smokers (IMvigor210: HR, 2.00; 95% CI, 1.32–3.03; *p* < 0.001; UC‐GENOME: HR, 1.79; 95% CI, 0.94–3.40; *p* = 0.08) and in the “excluded” immunophenotype subgroup (IMvigor210: HR, 1.57; 95% CI, 0.96–2.56; *p* = 0.07; UC‐GENOME: HR, 2.08; 95% CI, 0.92–4.74; *p* = 0.08).

In the IMvigor210 cohort, the MUT2 subtype was associated with a higher risk of death than the MUT1 subtype in patients with liver (HR, 1.88; 95% CI, 1.09–3.24; *p* = 0.02) or visceral metastases (HR, 1.73; 95% CI, 1.09–2.74; *p* = 0.02). It was also associated with a higher risk in patients with an IC PD‐L1 score of 0 at enrollment (IC0; HR, 2.05; 95% CI, 1.13–3.72; *p* = 0.02), an IC‐only PD‐L1 score of 0 (IC0; HR, 2.37; 95% CI, 1.28–4.29; *p* = 0.01), or a tumor cell PD‐L1 score of 0 (TC0; HR, 1.96; 95% CI, 1.37–2.82; *p* < 0.001). Additionally, the MUT2 subtype was associated with significantly worse survival in the “desert” immunophenotype subgroup (HR, 2.37; 95% CI, 1.27–4.45; *p* < 0.001).

### 3.4. Subgroup Analysis

Univariate analysis demonstrated that the mutational subtype remained a stable prognostic factor compared with clinical variables such as sex, age, smoking status, and tumor mutational burden. In IMvigor210, MUT2 was associated with increased risk of death compared with MUT1 (HR, 1.74; 95% CI, 1.27–2.37; *p* < 0.001). In the UC‐GENOME cohort, the HR for the MUT2 subtype versus the MUT1 subtype was 1.54 (95% CI, 0.93–2.54; *p* = 0.09). TCGA cohort showed an HR of 1.48 (95% CI, 1.10–1.98; *p* = 0.009), while the MSK2022 cohort exhibited an HR of 1.37 (95% CI, 1.12–1.69; *p* = 0.003). In the MSK2015 cohort, the HR for the MUT2 subtype versus the MUT1 subtype was 3.43 (95% CI, 1.36–8.64; *p* = 0.009) (Supporting Information 11: Figure S10). Multivariate analysis confirmed that the mutational subtype was an independent prognostic factor in the MSK2022 cohort (HR, 1.34; 95% CI, 1.07–1.67; *p* = 0.011) (Supporting Information 12: Figure S11).

### 3.5. Cell Type Identification of Single‐Cell Sequencing for UC

**Figure 2:**
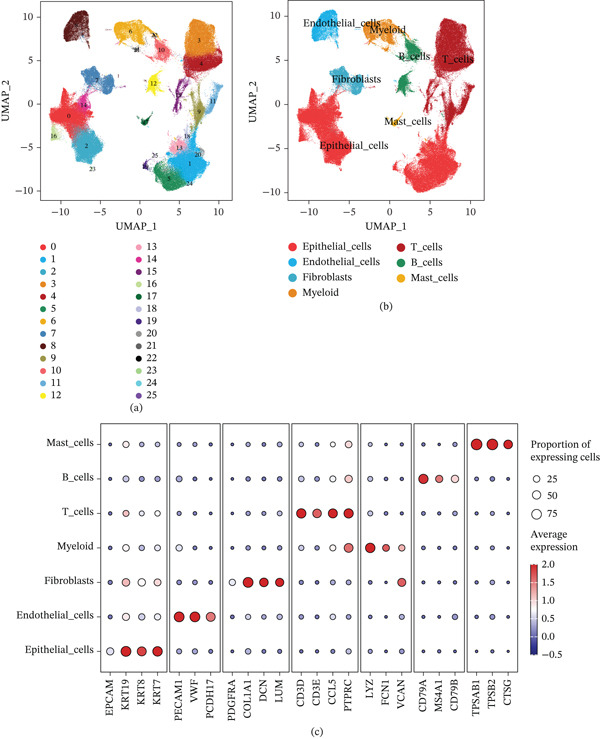
Single‐cell transcriptomic analysis of bladder cancer tissue reveals distinct cell populations and their marker genes. (a) UMAP plot of all profiled single cells, colored by unsupervised cluster. (b) UMAP plot of the same cells colored by manually annotated cell type, showing that the clusters correspond to epithelial tumor cells, fibroblasts, endothelial cells, myeloid cells, T cells, B cells, and mast cells. (c) Dot plot of canonical marker gene expression for each cell cluster, with selected marker genes on the *x*‐axis and cell clusters (grouped by cell type) on the *y*‐axis. Dot size represents the percentage of cells in each cluster expressing a given gene, while dot color (color intensity gradient) indicates the average expression level of that gene in the cluster. In the dot plot, each cell type′s clusters display the expected marker profile—for example, epithelial cell clusters express epithelial markers (e.g., EPCAM and keratins), fibroblast clusters express stromal markers (COL1A1 for collagen), endothelial clusters express vascular markers (PECAM1/CD31 and VWF), myeloid clusters express myeloid lineage markers (LYZ and CD68), T‐cell clusters express T‐lymphocyte markers (CD3D and CD3E), B‐cell clusters express B lineage markers (CD79A), and the mast cell cluster is characterized by high expression of mast cell tryptase (TPSAB1).

To elucidate the underlying mechanisms responsible for the disparities in prognosis and immunotherapy response between these two mutational subtypes, we performed a single‐cell–level interrogation of tumor microenvironment heterogeneity. This approach allowed us to systematically characterize the differential composition, transcriptional states, and cell–cell communication networks across distinct cellular clusters—including malignant, immune, and stromal populations—within each subtype. Single‐cell RNA sequencing (scRNA‐seq) of UC tissue yielded 26 transcriptionally distinct clusters of cells (Figure 2). Based on canonical marker genes, these clusters were annotated to all major cellular components of the bladder tumor microenvironment, including malignant epithelial cells, fibroblasts, endothelial cells, and diverse immune cells (T cells, B cells, myeloid cells, and a small population of mast cells).

### 3.6. Luminal‐Like Epithelial Cells Suggesting Immunotherapy Resistance and Enriched in MUT2 Subtype

**Figure 3:**
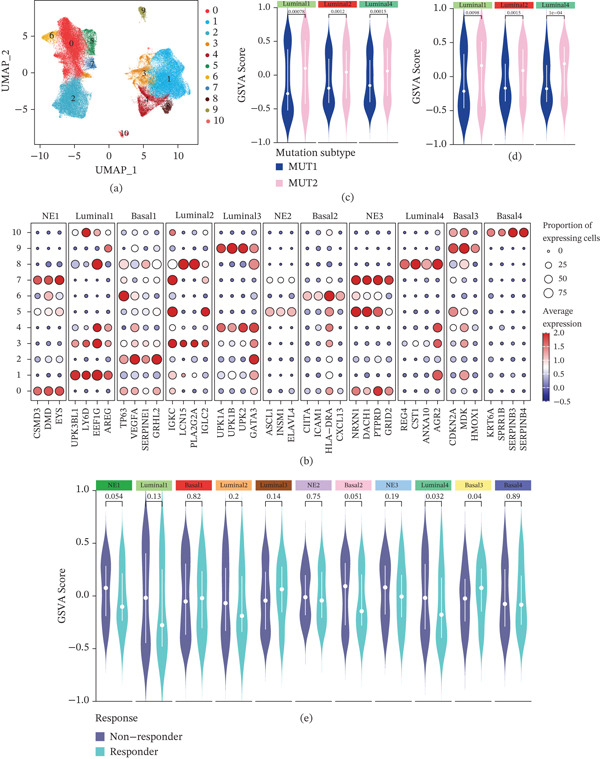
Single‐cell–defined epithelial subpopulations and their associations with genomic subtype and immunotherapy response. (a) UMAP embedding of reclustered bladder cancer epithelial cells, colored by 11 transcriptional subpopulations annotated as neuroendocrine‐like (NE1–3), luminal‐like (Lum1–4), and basal‐like (Bas1–4) phenotypes. (b, c) Violin plots of GSVA enrichment scores for luminal‐like subtype signatures (Lum1, Lum2, and Lum4) in TCGA and IMvigor210 stratified by mutational subtype (MUT1 vs. MUT2). Each violin shows the score distribution for each subpopulation signature within each mutational subtype; higher scores indicate stronger enrichment of that subtype. Statistical comparisons (two‐sided Wilcoxon′s rank‐sum tests) are indicated. (d) Dot plot of canonical marker gene expression across all 11 epithelial subtypes (columns). (e) Violin plots of GSVA scores for each subtype (NE1–3, Lum1–4, and Bas1–4) comparing patients who responded versus did not respond to immune checkpoint blockade. Each pair of violins shows the score distribution for a given subpopulation in responders versus nonresponders. Statistical significance (Wilcoxon′s rank‐sum test) is indicated. Abbreviations: GSVA, gene set variation analysis; NE, neuroendocrine‐like; Lum, luminal‐like; Bas, basal‐like.

Upon reclustering, epithelial cells were stratified into 11 distinct clusters. Based on their biological characteristics, three clusters (0, 5, and 7) were classified as neuroendocrine‐like epithelial cells (NE1–3), four clusters (1, 3, 4, and 8) as luminal‐like epithelial cells (Luminal1–4), and the remaining four clusters (2, 6, 9, and 10) as basal‐like epithelial cells (Basal1–4). Notably, luminal‐like epithelial cell clusters (Luminal1, Luminal2, and Luminal4) were consistently enriched in the MUT2 subtype across both primary and metastatic UC samples. Furthermore, a lower abundance of the Luminal4 epithelial subpopulation was observed in patients who responded to immunotherapy compared to nonresponders (Figure 3).

### 3.7. Fibroblast Subpopulations Are Indicative of Poor Prognosis and Therapeutic Resistance in UC

**Figure 4:**
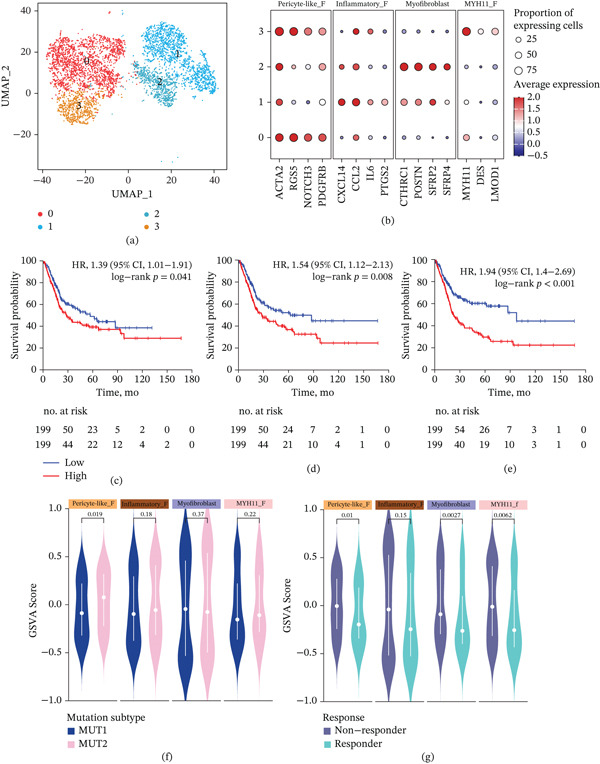
Fibroblast heterogeneity in bladder cancer and its clinical correlations. (a) UMAP plot of single‐cell RNA‐seq data from fibroblasts isolated from human bladder tumors. Four distinct fibroblast subtypes are shown. (b) Dot plot of selected canonical marker genes across the four fibroblast subpopulations. (c–e) Kaplan–Meier curves comparing overall survival of bladder cancer patients stratified by high versus low GSVA signature scores for three representative fibroblast subpopulations in TCGA cohort. Hazard ratios (HRs), 95% confidence intervals (CIs), and log‐rank *p* values are indicated on each plot, and the number of patients at risk in each group is shown below the *x*‐axis. (f) Violin plots comparing GSVA scores for the four fibroblast subpopulations between two mutational subtypes. Each violin depicts the distribution of signature scores; the white circle denotes the median, the box indicates the interquartile range, and whiskers extend to 1.5× IQR. *p* values above each pair (MUT1 vs. MUT2) were derived from a two‐tailed statistical test (Wilcoxon′s rank‐sum), showing significant differences in fibroblast signature abundance between the subtypes. (g) Violin plots comparing fibroblast subtype GSVA scores in patients who responded to immune checkpoint inhibitor therapy versus those who did not respond.

Through comprehensive transcriptomic profiling, we identified four distinct fibroblast subsets. These included a pericyte‐like, contractile CAF cluster (enriched for pericyte markers such as RGS5, NOTCH3, ACTA2, and PDGFRB), a myofibroblastic CAF cluster characterized by high extracellular matrix remodeling gene expression (e.g., CTHRC1, POSTN, and SFRP2), an inflammatory CAF cluster with a pronounced secretory phenotype (expressing cytokines/chemokines like IL6, CXCL12, and CXCL14), and a smooth muscle‐like fibroblast subset marked by MYH11 and other smooth muscle genes. Notably, the pericyte‐like and myofibroblastic CAFs represent contractile fibroblast phenotypes, whereas the inflammatory CAFs display an immune‐regulatory phenotype, highlighting functionally diverse roles within the tumor microenvironment (Figure 4a,b).

To evaluate the clinical significance of these fibroblast subpopulations, we quantified their enrichment in bulk tumors using GSVA scores in TCGA UC cohort. Kaplan–Meier survival analyses demonstrated that patients with high GSVA scores for certain CAF subpopulations had significantly worse OS than those with low scores (Figure 4c–e). For example, tumors enriched in the myofibroblastic signature showed poorer outcomes (HR = 1.39, 95% CI, 1.01–1.91; *p* = 0.041), suggesting that a dense, desmoplastic stroma portends aggressive disease. Likewise, a high inflammatory CAF signature was associated with shortened survival (HR = 1.54, 95% CI, 1.12–2.13; *p* = 0.008), consistent with reports that cytokine‐secreting CAFs can suppress antitumor immunity and drive tumor progression. Furthermore, the smooth muscle‐like MYH11 fibroblast signature was significantly prognostic (HR = 1.94, 95% CI, 1.4–2.69; *p* < 0.001), indicating a more invasive role for that subpopulation.

We further observed that fibroblast composition differed by tumor subtype and therapy response. The molecular subtype MUT2 exhibited significantly higher GSVA scores for all four fibroblast subpopulations compared to MUT1 (Figure 4f), implying that MUT2 tumors have a more CAF‐rich, stromal microenvironment. In particular, MUT2 tumors (putatively more aggressive) were enriched in inflammatory and pericyte‐like CAF signatures relative to MUT1. Finally, analyses of an immunotherapy‐treated patient cohort revealed associations between fibroblast subsets and anti‐PD‐1/PD‐L1 treatment response. Patients who responded to immune checkpoint blockade showed a distinct fibroblast signature profile: Notably, responders had diminished inflammatory CAF scores (*p* = 0.0062) and lower pericyte‐like and MYH11 CAF scores (*p* = 0.01 and *p* = 0.0027) compared to nonresponders (Figure 4g). Similar results were also observed in endothelial cell subsets (Supporting Information 13: Figure S12). Taken together, these findings highlight how fibroblast heterogeneity in BC impacts tumor biology and clinical outcomes, in line with evidence that activated CAF subsets are linked to advanced disease, immune evasion, and poorer patient survival.

Further analysis of immune‐related cells revealed distinct immunological profiles between the MUT1 and MUT2 subtypes. The MUT1 subtype exhibited increased infiltration of plasma cells and plasmablasts, whereas MUT2 demonstrated a higher prevalence of naïve B cells. Similarly, MUT1 showed elevated levels of effector CD8^+^ T cells and cycling CD8^+^ T cells, while MUT2 was characterized by a greater abundance of naïve T cells. This divergence in immune cell infiltration extended to the myeloid compartment, with MUT2 displaying reduced levels of classical CD14^+^ monocytes (Supporting Information 14: Figure S13, Supporting Information 15: Figure S14, and Supporting Information 16: Figure S15). Collectively, these findings suggest a more immunologically active neoplastic milieu in the MUT1 subtype, which may underlie the more favorable clinical outcomes observed in these patients.

### 3.8. Machine Learning Performance

**Figure 5:**
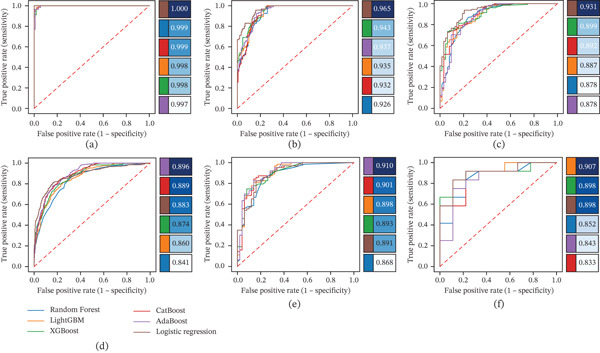
Receiver operating characteristic (ROC) curves comparing the performance of six machine learning classifiers in distinguishing between two mutational subtypes (MUT1 vs. MUT2) across six cohorts. The classifiers include Random Forest, LightGBM, XGBoost, CatBoost, AdaBoost, and logistic regression. All models were trained on TCGA training cohort and evaluated on (a) TCGA internal test set as well as five independent validation cohorts: (b) IMvigor210, (c) UC‐GENOME, (d) MSK2022, (e) MSK2015, and (f) Tongji. Each panel corresponds to one dataset and displays the ROC curves for all six models, with the legend indicating the AUC of each model in that cohort.

Logistic regression achieved the highest predictive performance on the held‐out TCGA test set, yielding the greatest area under the ROC curve (AUC). Ensemble methods (Random Forest, LightGBM, XGBoost, CatBoost, and AdaBoost) also achieved high AUC values, slightly below those of logistic regression. In external validation cohorts, logistic regression maintained strong discriminative performance between MUT1 and MUT2, with AUC values generally around or above 0.90 (Figure 5). For example, AUCs reached 0.963 in IMvigor210 and 0.931 in UC‐GENOME, representing top‐ranking performance in those validations. Performance remained robust even in the largest external set, MSK2022, and the smallest Tongji cohort (AUCs ~0.88 and ~0.84, respectively). Overall, all six machine learning models demonstrated robust and stable performance across cohorts, supporting the robustness and generalizability of the mutation subtype classification.

## 4. Discussion

In this study, we developed an approach based on mutational signature CS to identify stable molecular subtypes of UC. This method revealed distinct distributions of underlying mutational signatures between the MUT1 and MUT2 subtypes and provided biologically meaningful interpretations with corresponding prognostic and therapeutic implications. For example, patients with the MUT1 subtype exhibited enrichment of APOBEC‐associated mutational signatures (SBS2 and SBS13). In a mouse melanoma model, APOBEC3B‐mediated mutagenesis generated immunogenic neoepitopes in vaccine cells, activating de novo T‐cell responses and enhancing sensitivity to immune checkpoint blockade [58]. Consistent with these findings, our analysis of the IMvigor210 and UC‐GENOME cohorts showed that the MUT1 subtype was associated with a higher proportion of tumors with an “inflamed” immune phenotype [58–61]. Furthermore, our mutation classifier showed a strong correlation with the five subtypes of UC defined by Fujii et al. [26]. Specifically, MUT1 corresponded to the Fujii subtype with a higher proportion of TP53‐mutant cases, whereas MUT2 was significantly enriched in the triple‐negative subtype. The specific inactivating status of TP53 enhances sensitivity to immunotherapy, not primarily because the tumor becomes “more indolent” but because it becomes “more recognizable to the immune system” under certain molecular contexts. Jin et al. and Lyu et al. collectively point to a central axis involving higher mutational/neoantigen burden, stronger antigen presentation signals, and IFN‐*γ*/CD8‐mediated inflammation [62, 63]. Manzano et al. further trace this inflammatory state upstream to features such as a T‐cell‐inflamed signature, tumor‐infiltrating lymphocyte fraction, and APOBEC‐related mutational processes [64]. This explains why MUT1 is more sensitive to immunotherapy and is associated with a better prognosis. By contrast, the MUT2 subtype was enriched for “clock‐like,” DNA mismatch repair (MMR) deficiency, and exogenous chemotherapy‐associated mutational signatures (e.g., SBS1, SBS6, SBS15, and SBS87). This pattern aligns with our Cox regression analysis, which identified older age as a risk factor for UC, and with subgroup analyses showing that the MUT2 subtype was more prevalent in older patients. Both clinical evidence and cell/animal models suggest that MMR deficiency (reflected by SBS6/15 signatures) is associated with resistance to platinum‐based chemotherapy [65, 66]. Loss of MMR function impairs the ability of tumor cells to initiate apoptosis in response to platinum‐induced DNA damage, thereby promoting the selection of resistant clones and reducing treatment efficacy. Similar mechanisms have been reported in other malignancies, including MMR‐deficient lung and ovarian cancers, suggesting that comparable resistance mechanisms may operate in MMR‐deficient UC.

Our findings indicate that patients with the MUT2 subtype, characterized by MMR deficiency‐associated signatures, have a poorer prognosis than those with the MUT1 subtype, which is enriched for APOBEC signatures. This prognostic difference was observed across primary and metastatic settings and was consistent across multiple clinical subgroups. Specifically, MUT2 tumors exhibit a higher abundance of CAFs, including inflammatory and pericyte‐like CAF subpopulations, along with reduced infiltration of immune effector cells associated with reduced responsiveness to immunotherapy. We observed this trend among patients with a history of smoking, those with an ECOG performance status of 1, those lacking PD‐L1 expression on immune (IC0) and tumor cells (TC0), and those with the “desert” immunophenotype. A similar risk differential was noted even among patients who received platinum‐based chemotherapy and achieved a clinical benefit; in each of these contexts, the MUT2 subtype was associated with a significantly higher risk of death than the MUT1 subtype. These differences in prognostic and therapeutic response between patients with the MUT2 and MUT1 subtypes may reflect alterations in the tumor microenvironment driven by their distinct genomic profiles. Our findings offer insights for clinical practice by providing more refined mortality risk assessments and guiding the development of precise individualized treatment strategies.

This study has several limitations. First, differences in exome sequencing approaches across cohorts may have influenced the detection of mutational signatures because targeted sequencing panels typically cover fewer genes (and thus capture fewer mutations) than WES. Second, CS (a mathematical measure of how closely the shape of a sample′s mutational pattern matches a reference signature) was used to quantify the similarity between tumor mutational profiles and reference signatures; moderate similarity may reflect a mixture of multiple mutational processes rather than a single dominant signature. Third, although the machine learning models demonstrated strong performance across retrospective datasets, prospective validation and calibration will be required to determine optimal thresholds and implementation in clinical settings.

Despite these limitations, our findings were consistent across multiple cohorts and sequencing platforms, supporting the robustness and generalizability of this approach. Classification based on mutational signature similarity enabled reproducible risk stratification and provided insights into the biological mechanisms underlying subtype‐specific differences in treatment response. By linking mutational patterns to underlying carcinogenic processes, this framework may also facilitate the identification of novel therapeutic targets and improve patient stratification strategies.

## 5. Conclusion

In this retrospective study, we identified two distinct mutational signature subtypes of UC (MUT1 and MUT2), reflecting underlying tumor heterogeneity. MUT1, characterized predominantly by APOBEC‐associated mutational signatures, was associated with a more favorable prognosis and higher immunotherapy response rates. In contrast, MUT2, characterized by MMR deficiency–related signatures, was associated with poorer outcomes, including increased risk of death, particularly among patients receiving chemotherapy, with a history of smoking, or displaying certain immune phenotypes. These findings provide novel insights into clinical decision‐making for personalized treatment strategies and will help improve patient outcomes in UC by facilitating an exploration of resistance mechanisms, immunotherapy optimization, and long‐term outcomes and survivorship.

## Author Contributions

Chaozhi Tang was responsible for conceptualization, methodology, data curation, investigation, validation, formal analysis, visualization, and writing of the original draft, as well as review and editing of the manuscript. Yifan Liu and Tian‐Long Wang were responsible for methodology, software, data curation, validation, writing of the original draft, and resources. Jiakang Ma was responsible for methodology and software. Weihua Chen was responsible for data curation and investigation. Xiang Liu, Jingdong Xue, Lin Ye, and Feng Yue were responsible for methodology, resources, and supervision. Chaozhi Tang, Yifan Liu, and Tian‐Long Wang have contributed to the work equally and should be regarded as cofirst authors.

## Funding

This work was supported by the Shanghai Xuhui Medical Research Project (Grant Numbers SHXH202336 and SHXH202513), the Shanghai Eighth People′s Hospital Research Project (Grant Number SHBY202513), and the Medical Scientific Research Foundation of Guangdong Province, China (Grant Number B2022055).

## Disclosure

All authors have read and approved the final manuscript.

## Ethics Statement

This study was reviewed and approved by the Ethical Committee of the Shanghai East Hospital (No. 2018YS‐04) and complied with the principles of the Declaration of Helsinki. Written informed consent was obtained from all patients.

## Conflicts of Interest

The authors declare no conflicts of interest.

## Supporting information


**Supporting Information** Additional supporting information can be found online in the Supporting Information 1. **Supporting Information.** Methods S1: Computation and interpretation of cosine similarity. Methods S2: Identification of the mutational subtype of urothelial carcinoma. Methods S3: Identification of the risk score for urothelial carcinoma. Methods S4: Development and external validation of a machine learning–based prediction model. Supporting Information 2. Figure S1: Non‐negative matrix factorization (NMF) of the cosine similarity matrix of mutation signatures from TCGA cohort. Supporting Information 3. Figure S2: Identification and clinical characteristics of mutational signature classification in TCGA training cohort. Supporting Information 4. Figure S3: Clinical characteristics of mutational signature classifications in the MSK2022 test cohort. Supporting Information 5. Figure S4: Clinical characteristics according to the mutational signature classification in the MSK2015 test cohort. Supporting Information 6. Figure S5: Clinical characteristics of mutational signature classification in the IMvigor210 and UC‐GENOME test cohorts. Supporting Information 7. Figure S6: Kaplan–Meier curves depicting overall survival (OS) in patients stratified by the genomic mutation–based risk score: score < 1 (low risk) versus ≥ 1 (high risk). Supporting Information 8. Figure S7: Differences in clinical characteristics according to the mutation signature subtype and immunotherapy response. Supporting Information 9. Figure S8: Subgroup analysis of clinical characteristics based on the mutational subtype (IMvigor210 cohort). Supporting Information 10. Figure S9: Subgroup analysis of clinical characteristics based on the mutational subtype (UC‐GENOME cohort). Supporting Information 11. Figure S10: Univariate analysis for mutational signature subtype and clinical characteristics across multiple cohorts. Supporting Information 12. Figure S11: Multivariate analysis for mutational signature subtype and clinical characteristics across multiple cohorts. Supporting Information 13. Figure S12: Endothelial subpopulations are indicative of poor prognosis and therapeutic resistance in urothelial carcinoma. Supporting Information 14. Figure S13: Distribution of myeloid cells in mutational subtypes. Supporting Information 15. Figure S14: Distribution of B cells in mutational subtypes. Supporting Information 16. Figure S15: Distribution of T cells in mutational subtypes. Supporting Information 17. Table S1: Mutational signatures used in mutational subtype classifier. Table S2: Association of genetic alterations with overall survival in the multivariable regression analysis. Table S3: Distribution of clinical and genomic features by mutational signature subtype in the IMvigor210 cohort. Table S4: Distribution of clinical and genomic features by mutational signature subtype in the UC‐GENOME cohort. Table S5: Distribution of clinical and genomic features by mutational signature subtype in TCGA cohort. Table S6: Distribution of clinical and genomic features by mutational signature subtype in the MSK2022 cohort. Table S7: Distribution of clinical and genomic features by mutational signature subtype in the MSK2015 cohort.

## Data Availability

The data supporting the findings of this study are available from the corresponding authors upon reasonable request.
