# Supplementary material for: Integrated Genomic and Single‐Cell Analysis Reveals Heterogeneity, Prognosis, and Treatment Vulnerability in Urothelial Carcinoma
Source: Hum Mutat. 2026 May 27;2026:2797474. doi: 10.1155/humu/2797474 (PMC13213714; doi:10.1155/humu/2797474)
Supplement: Supplementary file 1 — Supporting Information Additional supporting information can be found online in the Supporting Information 1. Supporting Information. Methods S1: Computation and interpretation of cosine similarity. Methods S2: Identification of the mutational subtype of urothelial carcinoma. Methods S3: Identification of the risk score for urothelial carcinoma. Methods S4: Development and external validation of a machine learning–based prediction model. Supporting Information 2. Figure S1: Non‐negative matrix factorization (NMF) of the cosine similarity matrix of mutation signatures from TCGA cohort. Supporting Information 3. Figure S2: Identification and clinical characteristics of mutational signature classification in TCGA training cohort. Supporting Information 4. Figure S3: Clinical characteristics of mutational signature classifications in the MSK2022 test cohort. Supporting Information 5. Figure S4: Clinical characteristics according to the mutational signature classification in the MSK2015 test cohort. Supporting Information 6. Figure S5: Clinical characteristics of mutational signature classification in the IMvigor210 and UC‐GENOME test cohorts. Supporting Information 7. Figure S6: Kaplan–Meier curves depicting overall survival (OS) in patients stratified by the genomic mutation–based risk score: score < 1 (low risk) versus ≥ 1 (high risk). Supporting Information 8. Figure S7: Differences in clinical characteristics according to the mutation signature subtype and immunotherapy response. Supporting Information 9. Figure S8: Subgroup analysis of clinical characteristics based on the mutational subtype (IMvigor210 cohort). Supporting Information 10. Figure S9: Subgroup analysis of clinical characteristics based on the mutational subtype (UC‐GENOME cohort). Supporting Information 11. Figure S10: Univariate analysis for mutational signature subtype and clinical characteristics across multiple cohorts. Supporting Information 12. Figure S11: Multivariate analysis for mutational s [file HUMU-2026-2797474-s001.zip › Supplementary Materials.docx]

**Supplementary Materials**

**List of Contents**

Supplementary Methods 1. Computation and Interpretation of Cosine Similarity

Supplementary Methods 2. Identification of the Mutational Subtype of Urothelial Carcinoma

Supplementary Methods 3. Identification of the Risk Score for Urothelial Carcinoma

Supplementary Methods 4. Development and External Validation of a Machine Learning-Based Prediction Model

Supplementary Figure 1. Non-negative matrix factorization (NMF) of the cosine similarities matrix of mutation signatures from the TCGA cohort

Supplementary Figure 2. Identification and clinical characteristics of mutational signature classification in the TCGA training cohort

Supplementary Figure 3. Clinical characteristics of mutational signature classifications in the MSK2022 test cohort

Supplementary Figure 4. Clinical characteristics according to the mutational signature classification in the MSK2015 test cohort

Supplementary Figure 5. Clinical characteristics of mutational signature classification in the IMvigor210 and UC-GENOME test cohorts

Supplementary Figure 6. Kaplan–Meier curves depicting overall survival (OS) in patients stratified by the genomic mutation-based risk score: score <1 (low risk) vs. ≥1 (high risk)

Supplementary Figure 7. Differences in clinical characteristics according to the mutation signature subtype and immunotherapy response

Supplementary Figure 8. Subgroup analysis of clinical characteristics based on the mutational subtype (IMvigor210 cohort)

Supplementary Figure 9. Subgroup analysis of clinical characteristics based on the mutational subtype (UC-GENOME cohort)

Supplementary Figure 10. Univariate analysis for mutational signature subtype and clinical characteristics cross multiple cohorts

Supplementary Figure 11. Multivariate analysis for mutational signature subtype and clinical characteristics cross multiple cohorts

Supplementary Figure 12. Endothelial subpopulations are indicative of poor prognosis and therapeutic resistance in urothelial carcinoma

Supplementary Figure 13. Distribution of myeloid cells in mutational subtypes

Supplementary Figure 14. Distribution of B cells in mutational subtypes

Supplementary Figure 15. Distribution of T cells in mutational subtypes

Supplementary Table 1. Mutational signatures used in mutational subtypes classifier

Supplementary Table 2. Association of Genetic Alterations with Overall Survival in the Multivariable Regression Analysis

Supplementary Table 3. Distribution of Clinical and Genomic Features by Mutational Signature Subtype in the IMvigor210 Cohort

Supplementary Table 4. Distribution of Clinical and Genomic Features by Mutational Signature Subtype in the UC-GENOME Cohort

Supplementary Table 5. Distribution of Clinical and Genomic Features by Mutational Signature Subtype in the TCGA Cohort

Supplementary Table 6. Distribution of Clinical and Genomic Features by Mutational Signature Subtype in the MSK2022 Cohort

Supplementary Table 7. Distribution of Clinical and Genomic Features by Mutational Signature Subtype in the MSK2015 Cohort

**Supplementary Methods 1. Computation and Interpretation of Cosine Similarity**

**Cosine Similarity as a Measure of Profile Similarity**

To compare the similarity of a tumor’s mutational profile to a reference signature (or to compare any two mutational profiles), we employed the cosine-similarity metric. The cosine similarity measures the orientation (direction) similarity between two vectors in an *n*-dimensional space, independent of their magnitudes. In other words, it evaluates how closely aligned the two profiles are in terms of the distribution of mutation types rather than comparing their absolute mutation counts. Mathematically, the cosine similarity between the two vectors is defined as the dot product of the vectors divided by the product of their Euclidean norms. This is equivalent to the cosine of the angle between the two vectors. For the two mutational profile vectors *A* and *B*, the formula is as follows:

$$Cosine Similarity (A,B)=\frac{\sum_{i=1}^{n} A_{i} B_{i}}{\sqrt{\sum_{i=1}^{n} {A_{i}}^{2}}*\sqrt{\sum_{i=1}^{n} {B_{i}}^{2}}}$$

Here, 𝐴 = (𝐴_1_,𝐴_2_,…,𝐴_𝑛_) and 𝐵 = (𝐵_1_,𝐵_2_,…,𝐵_𝑛_) are the two vectors being compared (for example, 𝐴 could be a known signature’s vector, and *B* could be a tumor sample’s mutational profile vector). The index 𝑖 iterates over the mutation type categories (from 1 to 𝑛, with 𝑛=96 in the single base substitution (SBS) context), so that *𝐴_𝑖_* and *B_i_* represent the values corresponding to the same mutation type in each profile.

**Interpretation of the Cosine Similarity Value**

​The resulting cosine similarity is a unitless score ranging from –1 to 1. In the context of mutational signature analysis, all vector components *A_i_* and *B_i_* are typically non-negative because they represent frequencies or probabilities of mutations; therefore, the cosine similarity in practice ranges from 0 to 1 for realistic mutation data. A cosine similarity of 1 indicates that the two profiles are identical in shape. The vectors point in exactly the same direction in the mutation space, indicating that the relative contribution of each mutation type is the same in both profiles. A value close to zero indicates that the two profiles share very little similarity in their mutational patterns (the vectors are nearly orthogonal, meaning that when one profile has high values for certain mutation types, the other tends to have low values for those types, and vice versa). Intermediate values (e.g., 0.5, 0.8) represent varying degrees of partial similarity or pattern alignment between the mutation spectra.

​

​ **Supplementary Methods 2. Identification of the Mutational Subtype of Urothelial Carcinoma**

At rank 2, the best classification clusters were obtained using non-negative matrix factorization (NMF) on the cosine similarities (CSs) matrix composed of mutation signatures from The Cancer Genome Atlas (TCGA) cohort. Based on the COSMIC database^1^ and functional annotation of mutational signatures by Damrauer et al.,^2^ we classified the identified mutational subtypes and found that Cluster 1 (defined as the MUT1 subtype) was mainly associated with APOBEC activity events, such as SBS2, SBS13, and ultraviolet light exposure (SBS7a and SBS7b). Cluster 2 (defined as the MUT2 subtype) seemed to mainly accumulate DNA mismatch repair (MMR) deficiency events, such as SBS1 (“clock” like), SBS6 (defective mismatch repair), SBS15 (defective mismatch repair), and SBS87 (exogenous chemotherapy). Subsequently, we used the TCGA-derived mutational signature centroids and their cluster labels as a training reference in a nearest-centroid classifier (implemented via the “pamr” R package).^3^ This classifier was applied to independent cohorts, including the IMvigor210 trial cohort, the UC-GENOME study population, the MSK2022 and MSK2015 datasets, and the Tongji validation cohort, which assigned samples to the MUT1 and MUT2 subtypes in a manner highly consistent with the original clustering. In each of these external validation sets, we observed a similar two-subtype pattern, underscoring the robustness and reproducibility of the NMF-derived mutational subtype classification across diverse patient cohorts (Supplementary Figure 1).

**Supplementary Methods 3. Identification of the Risk Score for Urothelial Carcinoma**

Similar to the approach used by Long et al.,^4^ we identified a risk score for urothelial carcinoma based on the gene mutation status. Because the MSK2022 cohort included the largest number of urothelial carcinoma cases, we used it as a training set to develop this risk score. Propensity score matching (PSM) was employed to balance potential confounders such as age, sex, tumor mutational burden, and disease state (low-grade primary tumors, non-invasive and invasive high-grade [HG] primary tumors, and metastatic sites) between the mutant and wild-type groups for each gene in the MSK-IMPACT panel. Propensity scores were estimated using logistic regression (with gene mutation status as the dependent variable), and a PSM-based weighting scheme was subsequently applied to assign continuous weights to each sample according to these scores, thereby achieving a covariate balance.^5^

Genes that had both a nominal P value and a multiple-testing-adjusted P value < 0.05 were considered significantly associated with prognosis and were retained for further analysis. In the training cohort, we applied three sequential methods to identify prognostic genes and constructed a mutation-based gene set using PSM analysis, Lasso-penalized Cox regression, and multivariate Cox regression. First, genes were considered significant if the PSM analysis yielded a P value of < 0.05 (the PSM algorithm was implemented as described above). Second, to prevent overfitting and reduce multicollinearity, we performed a Lasso-penalized Cox proportional hazards regression using the glmnet R package (version 4.0-2) to further narrow down the candidate prognostic genes. The LASSO imposes a penalty on the regression coefficients, shrinking many of them toward zero, thereby selecting a subset of key prognostic genes.^6^ We performed 10-fold cross-validation to determine the optimal value of the penalty parameter (lambda). At this optimal lambda value, most candidate gene coefficients were driven to zero, leaving only a small number of genes with nonzero coefficients. Finally, to evaluate the stability of the LASSO selection, we generated 1,000 bootstrap resamples of the training dataset. Genes that maintained non-zero coefficients in more than 100 of these 1,000 resamples were deemed robust and retained in the final mutation-based gene set.

$risk score = \sum_{i=1}^{n} coef_{i}\times\mathrm{mu}t_{i}$ + 1

The coefficients (coef_1_, coef_2_, …, coef_n_) measure the impact (i.e., effect size) of the covariates.

mut_i_ is the value of the ith covariate from the subjects.

Using the optimal survival-based score threshold of 1, we stratified patients with urothelial carcinoma into low-risk (score < 1) and high-risk (score ≥ 1) groups, and applied this threshold to the IMvigor210, UC-GENOME, TCGA, MSK2015, and Tongji cohorts for risk stratification.

**Supplementary Methods 4. Development and External Validation of a Machine Learning-Based Prediction Model**

To further evaluate the discriminative ability of the predefined binary classification label in urothelial carcinoma, we developed a supervised machine learning workflow using the TCGA cohort as the model-development cohort. The dependent variable was the binary outcome label coded as y, and all remaining variables in the input matrix were used as candidate predictors. Samples from the TCGA cohort were randomly divided into a training set and an internal test set at a ratio of 7:3 using stratified sampling to preserve the distribution of the outcome classes. A fixed random seed of 42 was used to ensure reproducibility.

Six supervised classification algorithms were trained on the TCGA training set, including Random Forest, LightGBM, XGBoost, CatBoost, AdaBoost, and logistic regression. Default model parameters were used unless otherwise specified. For XGBoost, the log-loss evaluation metric was applied, and the logistic regression model was fitted with a maximum of 1,000 iterations. After model training, predicted probabilities for the positive class were generated for each sample using the fitted classifiers.

**Supplementary Figure 1. Non-negative matrix factorization (NMF) of the cosine similarities matrix of mutation signatures from the TCGA cohort.**

**Supplementary Figure 2. Identification and** **clinical characteristics of mutational signature classification in the TCGA training cohort.**

(A) Heatmap showing the distribution of cosine similarity and clinical characteristics of mutational signature classification. (B) Kaplan–Meier survival analysis showing the differences in disease-free survival between mutational signature subtypes. (C) Bar plot showing the tumor mutational burden (TMB) from whole-exome sequencing in the TCGA cohort (two-tailed Mann–Whitney U test; P < .001). Mb, megabase; MS, mutational signature.

**Supplementary Figure 3. Clinical characteristics of mutational signature classifications in the MSK2022 test cohort.**

(A) Heatmap showing the distribution of cosine similarity and clinical characteristics according to the mutational signature classification. (B) Bar plot showing the tumor mutational burden (TMB) from the target gene panel sequencing in the TCGA cohort (two-tailed Mann–Whitney U test; P < .001). Mb, megabase; MS, mutational signature.

**Supplementary Figure 4. Clinical characteristics according to the mutational signature classification in the MSK2015 test cohort.**

(A) Heatmap showing the distribution of cosine similarity and clinical characteristics according to the mutational signature classification. (B) Kaplan–Meier survival analysis showing the differences in disease-specific survival between mutational signature subtypes. (C) Bar plot showing the tumor mutational burden (TMB) from whole-exome sequencing in the TCGA cohort (two-tailed Mann–Whitney U test; P < .001). Mb, megabase; MS, mutational signature.

**Supplementary Figure 5. Clinical characteristics of mutational signature classification in the IMvigor210 and UC-GENOME test cohorts.**

(A) Heatmap showing the distribution of cosine similarity and clinical characteristics according to the mutational signature classification in the (A) IMvigor210 and (B) UC-GENOME test cohorts. Mb, megabase; MS, mutational signature.

**Supplementary Figure 6. Kaplan–Meier curves depicting overall survival (OS) in patients stratified by the genomic mutation-based risk score: score <1 (low risk) vs. ≥1 (high risk).**

Panels A–F correspond to cohorts: The Cancer Genome Atlas (TCGA; A), IMvigor210 (B), UC‑GENOME (C), MSK2022 (D), MSK2015 (E), and Tongji (F). Each panel shows the hazard ratio (HR) with 95% confidence interval (CI) and log-rank P values. Time is in months.

**Supplementary Figure 7. Differences in clinical characteristics according to the mutation signature subtype and immunotherapy response.**

(A) Bar charts showing the frequency of mutations in the commonly mutated genes *RB1*, *TP53*, *PIK3CA*, *ATM*, *ERCC2*, *FGFR3*, and *FANCC* between immunotherapy responders and non-responders. (B) and (C) show the immunotherapy response and immune phenotype, respectively, stratified by the mutation signature subtype (MUT1 vs. MUT2). (D) show the tumor mutational burden (TMB) from whole-exome sequencing in the IMvigor210 cohort and (E) targeted gene panel sequencing in the UC-GENOME cohort, respectively, stratified by the mutation signature subtype (two-tailed Mann–Whitney U test; *P* < .001 for both comparisons between MUT1 and MUT2). ns: not significant.

**Supplementary Figure 8. Subgroup analysis of clinical characteristics based on the mutational subtype (IMvigor210 cohort).**

ECOG, Eastern Cooperative Oncology Group performance status; IC, immune cell; TC, tumor cell; LN, lymph node; TMB, tumor mutation burden; Mb, megabase. HR, hazard ratio; CI, confidence interval.

**Supplementary Figure 9. Subgroup analysis of clinical characteristics based on the mutational subtype (UC-GENOME cohort).**

ECOG, Eastern Cooperative Oncology Group performance status; TMB, tumor mutation burden; Mb, megabase; CB, clinical benefit. HR, hazard ratio; CI, confidence interval.

**Supplementary Figure 10. Univariate analysis for Mutational signature subtype and clinical characteristics cross multiple cohorts.**

Mb, megabase; MS, mutational signature. TMB: tumor mutational burden.

**Supplementary Figure 11. Multivariate analysis for Mutational signature subtype and clinical characteristics cross multiple cohorts.**

Mb, megabase; MS, mutational signature. TMB: tumor mutational burden.

**Supplementary Figure 12. Endothelial subpopulations are indicative of poor prognosis and therapeutic resistance in urothelial carcinoma**

Endothelial cells heterogeneity in bladder cancer and its clinical correlations. (A) UMAP plot of single-cell RNA-seq data from endothelial cells isolated from human bladder tumors, four distinct fibroblast subtypes are shown. (B) Dot plot of selected canonical marker genes across the four endothelial subpopulations. (C–D) Kaplan–Meier curves comparing overall survival of bladder cancer patients stratified by high vs low GSVA signature scores for three representative endothelial subpopulations in the TCGA cohort. Hazard ratios (HR), 95% confidence intervals (CI), and log-rank p-values are indicated on each plot, and the number of patients at risk in each group is shown below the x-axis. (E) Violin plots showing significant differences in fibroblast signature abundance between the subtypes. (g) Violin plots comparing endothelial subpopulation GSVA scores in patients who responded to immune checkpoint inhibitor therapy versus those who did not respond.

**Supplementary Figure 13. Distribution of** **myeloid cells in mutational subtypes**

(A) UMAP embedding of re-clustered myeloid cells, colored by 7 transcriptional subpopulations annotated as classical monocytes, inflammatory monocytes, cDC2, LAMP3 monocytes, proliferating monocytes, cDC1 and CD207 DC. (B) Dot plot of selected canonical marker genes across the myeloid cell subpopulations. (C–D) Violin plots of GSVA enrichment scores for myeloid cells subcluster signatures in TCGA and IMvigor210 stratified by mutational subtype (MUT1 vs MUT2). Abbreviations: GSVA, gene set variation analysis; DC, dendritic cell.

**Supplementary Figure 14. Distribution of B cells in mutational subtypes**

(A) UMAP embedding of re-clustered B cells, colored by 6 transcriptional subpopulations annotated as Plasma, Naïve B cell, Extrafollicular B cell, Memory B cell, Plasmablast, Activate B cell. (B) Dot plot of selected canonical marker genes across the B cell subpopulations. (C–D) Violin plots of GSVA enrichment scores for B cells subcluster signatures in TCGA and IMvigor210 stratified by mutational subtype (MUT1 vs MUT2). Abbreviations: GSVA, gene set variation analysis.

**Supplementary Figure 15. Distribution of T cells in mutational subtypes**

(A) UMAP embedding of re-clustered T cells, colored by 6 transcriptional subpopulations annotated as CD8 exhausted progenitor cells (CD8_Pro), CD8 exhausted intermediate cells (CD8_Int), Naïve CD4 cell, Treg1, NK cell, Early CD8 cell, Cycling CD8 cell, Treg2, CD8 terminally exhausted cell (CD8_Tem) and γδ T cell. (B) Dot plot of selected canonical marker genes across the T cell subpopulations. (C–D) Violin plots of GSVA enrichment scores for T cells subcluster signatures in TCGA and IMvigor210 stratified by mutational subtype (MUT1 vs MUT2). (E) Violin plots comparing endothelial subpopulation GSVA scores in patients who responded to immune checkpoint inhibitor therapy versus those who did not respond.
Abbreviations: GSVA, gene set variation analysis.

**Supplementary Table 1. Mutational Signatures Used in Mutational Subtypes Classifier**

| eTable1 |  |  |  |
| --- | --- | --- | --- |
| SBS1 | SBS2 | SBS3 | SBS4 |
| SBS5 | SBS6 | SBS7a | SBS7b |
| SBS8 | SBS9 | SBS10b | SBS10c |
| SBS11 | SBS12 | SBS13 | SBS15 |
| SBS16 | SBS18 | SBS19 | SBS23 |
| SBS24 | SBS25 | SBS26 | SBS29 |
| SBS30 | SBS31 | SBS32 | SBS35 |
| SBS36 | SBS37 | SBS39 | SBS40 |
| SBS41 | SBS42 | SBS44 | SBS50 |
| SBS57 | SBS58 | SBS84 | SBS86 |
| SBS87 | SBS89 | SBS92 | SBS93 |
| SBS94 | SBS95 |  |  |

**Supplementary Table 2. Association of Genetic Alterations with Overall Survival in the Multivariable Regression Analysis**

| **Gene** | **Coef.** | **Exp (Coef.)** | **SE (Coef.)** | **z** | **P-value** |
| --- | --- | --- | --- | --- | --- |
| *FGFR3* | -3.90E-01 | 6.77E-01 | 1.22E-01 | -3.21 | 0.001327 |
| *PAK5* | -1.92E+00 | 1.47E-01 | 1.00E+00 | -1.91 | 0.056082 |
| *ERCC2* | -5.26E-01 | 5.91E-01 | 1.84E-01 | -2.863 | 0.004192 |
| *ERCC3* | -1.95E+00 | 1.42E-01 | 1.00E+00 | -1.948 | 0.051424 |
| *STAG2* | -3.78E-01 | 6.85E-01 | 1.78E-01 | -2.118 | 0.03418 |
| *CTCF* | -8.15E-01 | 4.43E-01 | 4.56E-01 | -1.787 | 0.073871 |
| *DAXX* | 8.70E-01 | 2.39E+00 | 2.58E-01 | 3.375 | 0.000738 |
| *BRCA2* | -3.77E-01 | 6.86E-01 | 2.20E-01 | -1.712 | 0.086921 |
| *PDGFRB* | -6.84E-01 | 5.05E-01 | 4.17E-01 | -1.641 | 0.100843 |
| *LATS1* | -6.01E-01 | 5.48E-01 | 3.21E-01 | -1.874 | 0.060919 |
| *MYOD1* | -1.58E+01 | 1.39E-07 | 1.24E+03 | -0.013 | 0.989877 |

SE: standard error

**Supplementary Table 3. Distribution of Clinical and Genomic Features by Mutational Signature Subtype in the IMvigor210 Cohort**

| **Characteristic** | **MUT1 (%)** | **MUT2 (%)** | **P-value** |
| --- | --- | --- | --- |
| Sex |  |  |  |
| Male | 122 (81.3) | 72 (76.6) | 0.466 |
| Female | 28 (18.7) | 22 (23.4) |  |
| Smoker |  |  |  |
| Current | 16 (10.7) | 9 (9.6) | 0.770 |
| Never | 43 (28.7) | 31 (33.0) |  |
| Former | 91 (60.6) | 54 (57.4) |  |
| TMB |  |  |  |
| < 10 Muts/Mb | 123 (82) | 90 (95.7) | **0.003** |
| ≥ 10 Muts/Mb | 27 (18) | 4 (4.3) |  |
| risk |  |  |  |
| low | 83 (55.3) | 38 (40.4) | **0.033** |
| high | 67 (44.7) | 56 (59.6) |  |
| Five subtype |  |  |  |
| Hypermutated | 1 (0.7) | 0 (0) | **3.652e-05** |
| *FGFR3* | 22 (14.7) | 19 (20.2) |  |
| *RAS* | 4 (2.7) | 3 (3.2) |  |
| *TP53*/*MDM2* | 79 (52.6) | 20 (21.3) |  |
| Triple negative | 44 (29.3) | 52 (55.3) |  |
| Received platinum |  |  |  |
| No | 40 (26.7) | 27 (28.7) | 0.839 |
| Yes | 110 (73.3) | 67 (71.2) |  |
| Metastatic site |  |  |  |
| Liver | 34 (22.7) | 35 (37.2) | **0.036** |
| LN Only | 32 (21.3) | 12 (12.8) |  |
| Visceral | 66 (44) | 41 (43.6) |  |
| NA | 18 (12) | 6 (6.4) |  |
| ECOG Score |  |  |  |
| 0 | 55 (36.7) | 40 (42.6) | 0.472 |
| 1 | 85 (56.7) | 50 (53.2) |  |
| 2 and 3 | 10 (6.6) | 4 (4.2) |  |
| Immune phenotype |  |  |  |
| Desert | 30 (20) | 25 (26.6) | **0.049** |
| Excluded | 53 (35.3) | 40 (42.5) |  |
| Inflamed | 48 (32) | 15 (16.0) |  |
| NA | 19 (12.7) | 14 (14.9) |  |
| Immunotherapy Response |  |  |  |
| Responder | 42 (28) | 11 (11.7) | **0.007** |
| Non-Responder | 90 (60) | 73 (77.7) |  |
| NA | 18 (12) | 10 (10.6) |  |
| Enrollment IC |  |  |  |
| IC0 | 31 (20.7) | 30 (31.9) | 0.107 |
| IC1 | 58 (38.7) | 35 (37.2) |  |
| IC2 | 61 (40.6) | 29 (30.9) |  |
| IC Level |  |  |  |
| IC0 | 30 (20) | 30 (31.9) | 0.079 |
| IC1 | 58 (38.7) | 35 (37.2) |  |
| IC2+ | 62 (41.3) | 29 (30.9) |  |
| TC Level |  |  |  |
| **Characteristic** | **MUT1 (%)** | **MUT2 (%)** | **P-value** |
| TC0 | 109 (72.7) | 73 (77.7) | 0.376 |
| TC1 | 10 (6.6) | 8 (8.5) |  |
| TC2+ | 31 (20.7) | 13 (13.8) |  |
| Consensus subtype |  |  |  |
| LumU | 8 (5.3) | 1 (1.1) | 0.185 |
| LumP | 9 (6) | 13 (13.8) |  |
| LumNS | 7 (4.7) | 5 (5.3) |  |
| Ba/Sq | 39 (26) | 26 (27.7) |  |
| Stroma-rich | 77 (51.4) | 46 (48.9) |  |
| NE-like | 2 (1.3) | 0 (0) |  |
| NA | 8 (5.3) | 3 (3.2) |  |

TMB: tumor mutation burden; LN: lymph node; ECOG: Eastern Cooperative Oncology Group; IC: immune cell; TC: tumor cell; LumU: luminal unstable; LumP: luminal papillary; LumNS: luminal not specific; Ba/Sq: basal/squamous; NE: neuroendocrine; Mb: megabase.

**Supplementary Table 4. Distribution of Clinical and Genomic Features by Mutational Signature Subtype in the UC-GENOME Cohort**

| **Characteristic** | **MUT1 (%)** | **MUT2 (%)** | **P-value** |
| --- | --- | --- | --- |
| Sex |  |  |  |
| Male | 47 (74.6) | 96 (75) | 0.772 |
| Female | 16 (25.4) | 31 (24.2) |  |
| Unknown | 0 (0) | 1 (0.8) |  |
| Age |  |  |  |
| ≤65 | 20 (31.7) | 63 (49.2) | 0.281 |
| >65 | 43 (68.3) | 65 (50.8) |  |
| Smoker |  |  |  |
| Current | 6 (9.5) | 10 (7.8) | 0.874 |
| Never | 21 (33.3) | 41 (32.0) |  |
| Former | 36 (57.2) | 76 (59.4) |  |
| Unknown | 0 (0) | 1 (0.8) |  |
| TMB |  |  |  |
| < 10 Muts/Mb | 10 (15.9) | 66 (51.6) | **1.027e-05** |
| ≥ 10 Muts/Mb | 39 (61.9) | 42 (32.8) |  |
| NA | 14 (22.2) | 20 (15.6) |  |
| risk |  |  |  |
| low | 36 (57.1) | 61 (47.7) | 0.281 |
| high | 27 (42.9) | 67 (52.3) |  |
| Five subtype |  |  |  |
| Hypermutated | 1 (1.6) | 1 (0.8) | 0.312 |
| FGFR3 | 8 (12.7) | 18 (14.1) |  |
| RAS | 0 (0) | 8 (6.3) |  |
| TP53/MDM2 | 38 (60.3) | 67 (52.3) |  |
| Triple negative | 16 (25.4) | 34 (26.5) |  |
| ECOG Score |  |  |  |
| 0 | 28 (44.4) | 50 (39.0) | 0.303 |
| 1 | 22 (35.0) | 59 (46.1) |  |
| 2 and 3 | 13 (20.6) | 19 (14.8) |  |
| Immune phenotype |  |  |  |
| Desert | 0 (0) | 1 (0.8) | **0.030** |
| Excluded | 22 (35.0) | 64 (50) |  |
| Inflamed | 29 (46.0) | 32 (25) |  |
| IND | 3 (4.7) | 15 (11.7) |  |
| NA | 9 (14.3) | 16 (12.5) |  |
| Chemo_CB |  |  |  |
| Benefit | 12 (19.0) | 29 (22.6) | 0.646 |
| No-Benefit | 6 (9.5) | 8 (6.3) |  |
| NA | 45 (71.5) | 91 (71.1) |  |
| Immuno_CB |  |  |  |
| Benefit | 19 (30.2) | 40 (31.3) | 0.485 |
| No-Benefit | 7 (11.1) | 22 (17.2) |  |
| NA | 37 (58.7) | 66 (51.5) |  |
| Chemotherapy Response |  |  |  |
| Responder | 15 (23.8) | 26 (20.3) | 0.881 |
| Non-Responder | 7 (11.1) | 17 (13.3) |  |
| NA | 41 (65.1) | 85 (66.4) |  |
| Immunotherapy  Response |  |  |  |
| Responder | 18 (28.6) | 23 (18.0) | 0.095 |
| Non-Responder | 15 (23.8) | 48 (37.5) |  |
| NA | 30 (47.6) | 57 (44.5) |  |
| Sample Site |  |  |  |
| Bladder | 44 (69.8) | 94 (73.4) | 0.726 |
| Non-Bladder | 19 (30.2) | 34 (26.6) |  |
| Consensus subtype |  |  |  |
| LumU | 7 (11.1) | 16 (12.5) | 0.270 |
| LumP | 9 (14.3) | 15 (11.7) |  |
| LumNS | 2 (3.2) | 2 (1.6) |  |
| Ba/Sq | 20 (31.7) | 33 (25.8) |  |
| Stroma-rich | 13 (20.7) | 45 (35.1) |  |
| NE-like | 3 (4.7) | 1 (0.8) |  |
| NA | 9 (14.3) | 16 (12.5) |  |

TMB: tumor mutation burden; ECOG: Eastern Cooperative Oncology Group; IND: indeterminate; CB: clinical benefit; LumU: luminal unstable; LumP: luminal papillary; LumNS: luminal not specific; Ba/Sq: basal/squamous; NE: neuroendocrine; Mb: megabase.

**Supplementary Table 5. Distribution of Clinical and Genomic Features by Mutational Signature Subtype in the TCGA Cohort**

| **Characteristic** | **MUT1 (%)** | **MUT2 (%)** | **P-value** |
| --- | --- | --- | --- |
| Sex |  |  |  |
| Male | 195 (77.7) | 109 (67.7) | **0.033** |
| Female | 56 (22.3) | 52 (32.3) |  |
| Age |  |  |  |
| ≤65 | 93 (37.1) | 69 (42.9) | 0.283 |
| >65 | 158 (62.9) | 92 (57.1) |  |
| Smoker |  |  |  |
| Current | 44 (17.5) | 46 (28.6) | 0.070 |
| Never | 72 (28.7) | 39 (24.2) |  |
| Former | 127 (50.6) | 71 (44.1) |  |
| Unknown | 8 (3.2) | 5 (3.1) |  |
| TMB |  |  |  |
| < 10 Muts/Mb | 169 (67.3) | 146 (90.7) | **9.703e-08** |
| ≥ 10 Muts/Mb | 82 (32.7) | 15 (9.3) |  |
| risk |  |  |  |
| low | 119 (47.4) | 54 (33.5) | **0.007** |
| high | 132 (52.6) | 107 (66.5) |  |
| Five subtype |  |  |  |
| Hypermutated | 0 (0) | 1 (0.6) | **0.002** |
| *FGFR3* | 25 (10.0) | 19 (11.8) |  |
| *RAS* | 10 (4.0) | 20 (12.4) |  |
| *TP53*/*MDM2* | 137 (54.6) | 62 (38.5) |  |
| Triple negative | 79 (31.4) | 59 (36.7) |  |
| T |  |  |  |
| T1 | 1 (0.4) | 0 (0) | 0.223 |
| T2 | 68 (27.1) | 55 (34.1) |  |
| T3 | 127 (50.6) | 69 (42.9) |  |
| T4 | 32 (12.7) | 27 (16.8) |  |
| NA | 23 (9.2) | 10 (6.2) |  |
| stage |  |  |  |
| I | 1 (0.4) | 0 (0) | 0.346 |
| II | 78 (31.1) | 54 (33.5) |  |
| III | 90 (35.9) | 51 (31.7) |  |
| IV | 82 (32.6) | 54 (33.5) |  |
| NA | 0 (0) | 2 (1.3) |  |
| Grade |  |  |  |
| Low Grade | 6 (2.4) | 15 (9.3) | **0.004** |
| High Grade | 244 (97.2) | 144 (89.4) |  |
| NA | 1 (0.4) | 2 (1.3) |  |
| TCGA |  |  |  |
| Luminal papillary | 77 (30.7) | 65 (40.4) | 0.267 |
| Luminal infiltrated | 54 (21.5) | 24 (14.9) |  |
| Luminal | 14 (5.6) | 12 (7.4) |  |
| Basal squamous | 91 (36.2) | 51 (31.7) |  |
| Neuronal | 12 (4.8) | 8 (5.0) |  |
| NA | 3 (1.2) | 1 (0.6) |  |
| Consensus subtype |  |  |  |
| LumU | 21 (8.4) | 7 (4.3) | 0.139 |
| LumP | 61 (24.3) | 59 (36.7) |  |
| LumNS | 25 (10.0) | 16 (10.0) |  |
| Ba/Sq | 92 (36.6) | 49 (30.4) |  |
| Stroma-rich | 45 (17.9) | 28 (17.4) |  |
| NE-like | 4 (1.6) | 1 (0.6) |  |
| NA | 3 (1.2) | 1 (0.6) |  |

TMB: tumor mutation burden; T: tumor; LumU: luminal unstable; LumP: luminal papillary; LumNS: luminal not specific; Ba/Sq: basal/squamous; NE: neuroendocrine; Mb: megabase.

**Supplementary Table 6. Distribution of Clinical and Genomic Features by Mutational Signature Subtype in the MSK2022 Cohort**

| **Characteristic** | **MUT1 (%)** | **MUT2 (%)** | **P-value** |
| --- | --- | --- | --- |
| Sex |  |  |  |
| Male | 656 (78.8) | 324 (72.8) | **0.027** |
| Female | 175 (21.0) | 121 (27.2) |  |
| NA | 2 (0.2) | 0 (0) |  |
| Age |  |  |  |
| ≤65 | 264 (31.7) | 150 (33.7) | 0.610 |
| >65 | 540 (64.8) | 283 (63.6) |  |
| NA | 29 (3.5) | 12 (2.7) |  |
| Smoker |  |  |  |
| Current | 84 (10.1) | 57 (12.8) | **0.007** |
| Never | 307 (36.9) | 126 (28.3) |  |
| Former | 403 (48.4) | 247 (55.5) |  |
| Unknown | 39 (4.6) | 15 (3.4) |  |
| TMB |  |  |  |
| < 10 Muts/Mb | 337 (40.4) | 335 (75.3) | **3.115e-32** |
| ≥ 10 Muts/Mb | 496 (59.6) | 110 (24.7) |  |
| risk |  |  |  |
| low | 429 (51.5) | 179 (40.2) | **0.00015** |
| high | 404 (48.5) | 266 (59.8) |  |
| Five subtype |  |  |  |
| Hypermutated | 2 (2.4) | 5 (1.1) | 0.327 |
| *FGFR3* | 157 (18.8) | 84 (18.9) |  |
| *RAS* | 45 (5.4) | 23 (5.1) |  |
| *TP53*/*MDM2* | 416 (49.9) | 213 (47.9) |  |
| Triple negative | 213 (25.5) | 120 (27.0) |  |
| Sample Type |  |  |  |
| Primary | 690 (82.7) | 353 (79.3) | 0.154 |
| Metastasis | 141 (16.9) | 92 (20.7) |  |
| Local Recurrence | 2 (2.4) | 0 (0) |  |
| Specimen Stage |  |  |  |
| low grade | 40 (4.8) | 26 (5.8) | 0.214 |
| HG Non-invasive | 143 (17.2) | 75 (16.9) |  |
| HG Invasive | 512 (61.4) | 252 (56.6) |  |
| Metastasis | 138 (16.6) | 92 (20.7) |  |
| Systemic Treatment |  |  |  |
| Naive | 642 (77.1) | 329 (73.9) | **0.004** |
| Chemotherapy | 140 (16.8) | 75 (16.9) |  |
| Immunotherapy | 7 (0.8) | 9 (2.0) |  |
| Both | 11 (1.3) | 19 (4.3) |  |
| Unknown | 33 (4.0) | 13 (2.9) |  |

TMB: tumor mutation burden; HG: high grade; Mb: megabase.

**Supplementary Table 7. Distribution of Clinical and Genomic Features by Mutational Signature Subtype in the MSK2015 Cohort**

| **Characteristic** | **MUT1 (%)** | **MUT2 (%)** | **P-value** |
| --- | --- | --- | --- |
| Sex |  |  |  |
| Male | 25 (67.6) | 29 (63.0) | 0.637 |
| Female | 12 (32.4) | 16 (34.8) |  |
| NA | 0 (0) | 1 (2.2) |  |
| Age |  |  |  |
| ≤65 | 13 (35.1) | 16 (34.8) | 0.665 |
| >65 | 24 (64.9) | 29 (63.0) |  |
| NA | 0 (0) | 1 (2.2) |  |
| Smoker |  |  |  |
| Current | 4 (10.8) | 12 (26.1) | 0.172 |
| Never | 12 (32.4) | 10 (21.7) |  |
| Former | 21 (56.8) | 23 (50) |  |
| Unknown | 0 (0) | 1 (2.2) |  |
| TMB |  |  |  |
| < 10 Muts/Mb | 21 (56.8) | 40 (87.0) | **0.004** |
| ≥ 10 Muts/Mb | 16 (43.2) | 6 (13) |  |
| risk |  |  |  |
| low | 26 (70.3) | 20 (43.5) | **0.027** |
| high | 11 (29.7) | 26 (56.5) |  |
| Five subtype |  |  |  |
| Hypermutated | 0 (0) | 1 (2.2) | **0.035** |
| *FGFR3* | 24 (64.9) | 15 (32.6) |  |
| *RAS* | 3 (8.1) | 8 (17.4) |  |
| *TP53*/*MDM2* | 6 (16.2) | 8 (17.4) |  |
| Triple negative | 4 (10.8) | 14 (30.4) |  |
| Adjuvant Chemotherapy |  |  |  |
| No | 27 (73.0) | 25 (54.3) | 0.249 |
| Yes | 10 (27.0) | 18 (39.1) |  |
| NA | 0 (0) | 3 (6.5) |  |
| Local Recurrence |  |  |  |
| No | 16 (43.2) | 27 (58.7) | 0.212 |
| Yes | 21 (56.8) | 18 (39.1) |  |
| NA | 0 (0) | 1 (2.2) |  |

TMB: tumor mutation burden; Mb: megabase.

**References**

1. Tate JG, Bamford S, Jubb HC, et al. COSMIC: the Catalogue Of Somatic Mutations In Cancer. *Nucleic Acids Res*. Jan 8 2019;47(D1):D941-D947. doi:10.1093/nar/gky1015

2. Damrauer JS, Beckabir W, Klomp J, et al. Collaborative study from the Bladder Cancer Advocacy Network for the genomic analysis of metastatic urothelial cancer. *Nat Commun*. Nov 4 2022;13(1):6658. doi:10.1038/s41467-022-33980-9

3. Tibshirani R, Hastie T, Narasimhan B, Chu G. Diagnosis of multiple cancer types by shrunken centroids of gene expression. *Proc Natl Acad Sci U S A*. May 14 2002;99(10):6567-72. doi:10.1073/pnas.082099299

4. Long J, Wang D, Wang A, et al. A mutation-based gene set predicts survival benefit after immunotherapy across multiple cancers and reveals the immune response landscape. *Genome Med*. Feb 24 2022;14(1):20. doi:10.1186/s13073-022-01024-y

5. Luo Z, Wang W, Li F, et al. Pan-cancer analysis identifies telomerase-associated signatures and cancer subtypes. *Mol Cancer*. Jun 10 2019;18(1):106. doi:10.1186/s12943-019-1035-x

6. Wu TT, Chen YF, Hastie T, Sobel E, Lange K. Genome-wide association analysis by lasso penalized logistic regression. *Bioinformatics*. Mar 15 2009;25(6):714-21. doi:10.1093/bioinformatics/btp041
