# Supplementary material for: Integrated Genomic and Single‐Cell Analysis Reveals Heterogeneity, Prognosis, and Treatment Vulnerability in Urothelial Carcinoma
Source: Hum Mutat. 2026 May 27;2026:2797474. doi: 10.1155/humu/2797474 (PMC13213714; doi:10.1155/humu/2797474)
Supplement: Supplementary file 1 — Supporting Information Additional supporting information can be found online in the Supporting Information 1. Supporting Information. Methods S1: Computation and interpretation of cosine similarity. Methods S2: Identification of the mutational subtype of urothelial carcinoma. Methods S3: Identification of the risk score for urothelial carcinoma. Methods S4: Development and external validation of a machine learning–based prediction model. Supporting Information 2. Figure S1: Non‐negative matrix factorization (NMF) of the cosine similarity matrix of mutation signatures from TCGA cohort. Supporting Information 3. Figure S2: Identification and clinical characteristics of mutational signature classification in TCGA training cohort. Supporting Information 4. Figure S3: Clinical characteristics of mutational signature classifications in the MSK2022 test cohort. Supporting Information 5. Figure S4: Clinical characteristics according to the mutational signature classification in the MSK2015 test cohort. Supporting Information 6. Figure S5: Clinical characteristics of mutational signature classification in the IMvigor210 and UC‐GENOME test cohorts. Supporting Information 7. Figure S6: Kaplan–Meier curves depicting overall survival (OS) in patients stratified by the genomic mutation–based risk score: score < 1 (low risk) versus ≥ 1 (high risk). Supporting Information 8. Figure S7: Differences in clinical characteristics according to the mutation signature subtype and immunotherapy response. Supporting Information 9. Figure S8: Subgroup analysis of clinical characteristics based on the mutational subtype (IMvigor210 cohort). Supporting Information 10. Figure S9: Subgroup analysis of clinical characteristics based on the mutational subtype (UC‐GENOME cohort). Supporting Information 11. Figure S10: Univariate analysis for mutational signature subtype and clinical characteristics across multiple cohorts. Supporting Information 12. Figure S11: Multivariate analysis for mutational s [file HUMU-2026-2797474-s001.zip › Supplementary Methods 4.html]

Untitled7


In [12]:

```
import pandas as pd
import numpy as np
import matplotlib.pyplot as plt
import warnings

# ====== 0) Global Plot Settings======
plt.rcParams['font.family'] = 'Times New Roman'
plt.rcParams['axes.unicode_minus'] = False
warnings.filterwarnings("ignore")

# ====== 1) Load training/testing data ======
path = r"/TCGA.xlsx"
df = pd.read_excel(path)

# ====== 2) Split into training set / test set ======
from sklearn.model_selection import train_test_split

X = df.drop(['y'], axis=1)
y = df['y']

X_train, X_test, y_train, y_test = train_test_split(
    X, y,
    test_size=0.3,
    random_state=42,
    stratify=y
)

# ====== 3) train model ======
from sklearn.linear_model import LogisticRegression
from sklearn.ensemble import RandomForestClassifier, AdaBoostClassifier
import lightgbm as lgb
import xgboost as xgb
from catboost import CatBoostClassifier

rf_model = RandomForestClassifier(random_state=42)
rf_model.fit(X_train, y_train)

lgb_model = lgb.LGBMClassifier(random_state=42, verbose=-1)
lgb_model.fit(X_train, y_train)

xgb_model = xgb.XGBClassifier(random_state=42, eval_metric='logloss')
xgb_model.fit(X_train, y_train)

catboost_model = CatBoostClassifier(random_state=42, verbose=0)
catboost_model.fit(X_train, y_train)

ada_model = AdaBoostClassifier(random_state=42)
ada_model.fit(X_train, y_train)

lr_model = LogisticRegression(random_state=42, max_iter=1000)
lr_model.fit(X_train, y_train)

# ====== 4) Unified model collection + fixed color mapping (critical)======
trained_models = {
    "RandomForest": rf_model,
    "LightGBM": lgb_model,
    "XGBoost": xgb_model,
    "CatBoost": catboost_model,
    "AdaBoost": ada_model,
    "Logistic Regression": lr_model
    # If you have stacked models, you may add them：
    # "Stacked Model": stacked_model
}

# Fixed color dictionary: reused for all subsequent plots, ensuring "same color for the same model”
colors = {name: f"C{i}" for i, name in enumerate(trained_models.keys())}

# ====== 5) General function: plot ROC + AUC bar on the right (and save as PDF)======
import matplotlib.gridspec as gridspec
from matplotlib.colors import Normalize
from matplotlib.cm import ScalarMappable
import matplotlib.patches as mpatches
from sklearn.metrics import roc_curve, auc

def plot_roc_with_auc_bar(y_true, X_data, trained_models, colors, title, pdf_name):
    """
    在给定数据集(X_data, y_true)上：
    - 左：ROC曲线（颜色来自 colors 字典）
    - 右：AUC条（左块=模型固定色；右块=按AUC渐变）
    并保存为PDF。
    """
    # 计算ROC与AUC
    results = []
    for name, model in trained_models.items():
        y_prob = model.predict_proba(X_data)[:, 1]
        fpr, tpr, _ = roc_curve(y_true, y_prob)
        roc_auc = auc(fpr, tpr)
        results.append({'name': name, 'fpr': fpr, 'tpr': tpr, 'auc': roc_auc})

    # AUC降序（右侧条形图显示用）
    sorted_results = sorted(results, key=lambda x: x['auc'], reverse=True)

    # 布局
    fig = plt.figure(figsize=(10, 7))
    gs = gridspec.GridSpec(1, 2, width_ratios=[4, 1], wspace=0.05)

    # --- 左：ROC曲线 ---
    ax_left = plt.subplot(gs[0])
    for res in results:
        model_name = res['name']
        style = '--' if model_name == 'Stacked Model' else '-'
        lw = 3 if model_name == 'Stacked Model' else 2.5
        ax_left.plot(
            res['fpr'], res['tpr'],
            lw=lw,
            color=colors[model_name],   # 统一颜色
            linestyle=style,
            label=model_name
        )

    # 
    ax_left.plot([0, 1], [0, 1], 'r--', linewidth=1.5, alpha=0.8)

    ax_left.set_xlim([-0.05, 1.05])
    ax_left.set_ylim([-0.05, 1.05])
    ax_left.set_xlabel("False Positive Rate (1-Specificity)", fontsize=18)
    ax_left.set_ylabel("True Positive Rate (Sensitivity)", fontsize=18)
    ax_left.set_title(title, fontsize=20, fontweight="bold")
    ax_left.legend(loc="lower right", fontsize=15, frameon=False)
    ax_left.tick_params(axis='both', which='major', labelsize=18)

    #  ---
    ax_right = plt.subplot(gs[1])
    ax_right.axis('off')

    norm = Normalize(vmin=min(r['auc'] for r in results), vmax=max(r['auc'] for r in results))
    cmap = plt.cm.Blues

    num_models = len(sorted_results)
    bar_height = 0.9

    for i, res in enumerate(sorted_results):
        y_pos = num_models - i - 1.1
        auc_value = res['auc']

        # 
        bg_color = ScalarMappable(norm=norm, cmap=cmap).to_rgba(auc_value)

        # 
        ax_right.add_patch(
            mpatches.Rectangle(
                (0, y_pos), 0.3, bar_height,
                facecolor=colors[res['name']], edgecolor='black', lw=1.5
            )
        )

        # 
        ax_right.add_patch(
            mpatches.Rectangle(
                (0.3, y_pos), 0.7, bar_height,
                facecolor=bg_color, edgecolor='black', lw=1.5
            )
        )

        # 
        text_color = 'white' if i < 3 else 'black'
        ax_right.text(
            0.65, y_pos + bar_height / 2,
            f"{auc_value:.3f}",
            ha='center', va='center',
            fontsize=18, color=text_color, fontweight='bold'
        )

    ax_right.set_ylim(-0.1, num_models - (1 - bar_height) - 0.1)
    ax_right.set_xlim(0, 1)

    # 
    fig.savefig(pdf_name, format='pdf', bbox_inches='tight', dpi=1200)
    plt.show()

    return results

# ====== 6) Test set: plot and save ======
plot_roc_with_auc_bar(
    y_true=y_test,
    X_data=X_test,
    trained_models=trained_models,
    colors=colors,
    title="ROC Curve - Test Set Model Comparison",
    pdf_name="ROC_Test.pdf"
)

# ====== 7)  Load independent validation sets======
df1 = pd.read_excel("/UC_GENOME.xlsx")
df2 = pd.read_excel("/IMvigor210_all.xlsx")

X1 = df1.drop(['y'], axis=1)
y1 = df1['y']

X2 = df2.drop(['y'], axis=1)
y2 = df2['y']

# ====== 8) ======
plot_roc_with_auc_bar(
    y_true=y1,
    X_data=X1,
    trained_models=trained_models,
    colors=colors,
    title="ROC Curve - Data1 Set Model Comparison",
    pdf_name="ROC_Data1.pdf"
)

# ====== 9)  ======
plot_roc_with_auc_bar(
    y_true=y2,
    X_data=X2,
    trained_models=trained_models,
    colors=colors,
    title="ROC Curve - Data2 Set Model Comparison",
    pdf_name="ROC_Data2.pdf"
)

print("Done! PDFs saved as: ROC_Test.pdf, ROC_Data1.pdf, ROC_Data2.pdf")
```

```
findfont: Font family 'Times New Roman' not found.
findfont: Font family 'Times New Roman' not found.
findfont: Font family 'Times New Roman' not found.
findfont: Font family 'Times New Roman' not found.
findfont: Font family 'Times New Roman' not found.
findfont: Font family 'Times New Roman' not found.
findfont: Font family 'Times New Roman' not found.
findfont: Font family 'Times New Roman' not found.
findfont: Font family 'Times New Roman' not found.
findfont: Font family 'Times New Roman' not found.
findfont: Font family 'Times New Roman' not found.
findfont: Font family 'Times New Roman' not found.
findfont: Font family 'Times New Roman' not found.
findfont: Font family 'Times New Roman' not found.
findfont: Font family 'Times New Roman' not found.
findfont: Font family 'Times New Roman' not found.
findfont: Font family 'Times New Roman' not found.
findfont: Font family 'Times New Roman' not found.
findfont: Font family 'Times New Roman' not found.
findfont: Font family 'Times New Roman' not found.
findfont: Font family 'Times New Roman' not found.
findfont: Font family 'Times New Roman' not found.
findfont: Font family 'Times New Roman' not found.
findfont: Font family 'Times New Roman' not found.
findfont: Font family 'Times New Roman' not found.
findfont: Font family 'Times New Roman' not found.
findfont: Font family 'Times New Roman' not found.
findfont: Font family 'Times New Roman' not found.
findfont: Font family 'Times New Roman' not found.
findfont: Font family 'Times New Roman' not found.
findfont: Font family 'Times New Roman' not found.
findfont: Font family 'Times New Roman' not found.
findfont: Font family 'Times New Roman' not found.
findfont: Font family 'Times New Roman' not found.
findfont: Font family 'Times New Roman' not found.
findfont: Font family 'Times New Roman' not found.
findfont: Font family 'Times New Roman' not found.
findfont: Font family 'Times New Roman' not found.
findfont: Font family 'Times New Roman' not found.
findfont: Font family 'Times New Roman' not found.
findfont: Font family 'Times New Roman' not found.
findfont: Font family 'Times New Roman' not found.
findfont: Font family 'Times New Roman' not found.
findfont: Font family 'Times New Roman' not found.
findfont: Font family 'Times New Roman' not found.
findfont: Font family 'Times New Roman' not found.
findfont: Font family 'Times New Roman' not found.
findfont: Font family 'Times New Roman' not found.
findfont: Font family 'Times New Roman' not found.
findfont: Font family 'Times New Roman' not found.
findfont: Font family 'Times New Roman' not found.
findfont: Font family 'Times New Roman' not found.
findfont: Font family 'Times New Roman' not found.
findfont: Font family 'Times New Roman' not found.
findfont: Font family 'Times New Roman' not found.
findfont: Font family 'Times New Roman' not found.
findfont: Font family 'Times New Roman' not found.
findfont: Font family 'Times New Roman' not found.
findfont: Font family 'Times New Roman' not found.
findfont: Font family 'Times New Roman' not found.
findfont: Font family 'Times New Roman' not found.
findfont: Font family 'Times New Roman' not found.
findfont: Font family 'Times New Roman' not found.
findfont: Font family 'Times New Roman' not found.
findfont: Font family 'Times New Roman' not found.
findfont: Font family 'Times New Roman' not found.
findfont: Font family 'Times New Roman' not found.
findfont: Font family 'Times New Roman' not found.
findfont: Font family 'Times New Roman' not found.
findfont: Font family 'Times New Roman' not found.
findfont: Font family 'Times New Roman' not found.
findfont: Font family 'Times New Roman' not found.
findfont: Font family 'Times New Roman' not found.
findfont: Font family 'Times New Roman' not found.
findfont: Font family 'Times New Roman' not found.
findfont: Font family 'Times New Roman' not found.
findfont: Font family 'Times New Roman' not found.
findfont: Font family 'Times New Roman' not found.
findfont: Font family 'Times New Roman' not found.
findfont: Font family 'Times New Roman' not found.
findfont: Font family 'Times New Roman' not found.
findfont: Font family 'Times New Roman' not found.
findfont: Font family 'Times New Roman' not found.
findfont: Font family 'Times New Roman' not found.
findfont: Font family 'Times New Roman' not found.
findfont: Font family 'Times New Roman' not found.
findfont: Font family 'Times New Roman' not found.
findfont: Font family 'Times New Roman' not found.
findfont: Font family 'Times New Roman' not found.
findfont: Font family 'Times New Roman' not found.
findfont: Font family 'Times New Roman' not found.
findfont: Font family 'Times New Roman' not found.
findfont: Font family 'Times New Roman' not found.
findfont: Font family 'Times New Roman' not found.
findfont: Font family 'Times New Roman' not found.
findfont: Font family 'Times New Roman' not found.
findfont: Font family 'Times New Roman' not found.
findfont: Font family 'Times New Roman' not found.
findfont: Font family 'Times New Roman' not found.
findfont: Font family 'Times New Roman' not found.
findfont: Font family 'Times New Roman' not found.
findfont: Font family 'Times New Roman' not found.
findfont: Font family 'Times New Roman' not found.
findfont: Font family 'Times New Roman' not found.
findfont: Font family 'Times New Roman' not found.
findfont: Font family 'Times New Roman' not found.
findfont: Font family 'Times New Roman' not found.
findfont: Font family 'Times New Roman' not found.
findfont: Font family 'Times New Roman' not found.
findfont: Font family 'Times New Roman' not found.
findfont: Font family 'Times New Roman' not found.
findfont: Font family 'Times New Roman' not found.
findfont: Font family 'Times New Roman' not found.
findfont: Font family 'Times New Roman' not found.
findfont: Font family 'Times New Roman' not found.
findfont: Font family 'Times New Roman' not found.
findfont: Font family 'Times New Roman' not found.
findfont: Font family 'Times New Roman' not found.
findfont: Font family 'Times New Roman' not found.
findfont: Font family 'Times New Roman' not found.
findfont: Font family 'Times New Roman' not found.
findfont: Font family 'Times New Roman' not found.
findfont: Font family 'Times New Roman' not found.
findfont: Font family 'Times New Roman' not found.
findfont: Font family 'Times New Roman' not found.
findfont: Font family 'Times New Roman' not found.
findfont: Font family 'Times New Roman' not found.
findfont: Font family 'Times New Roman' not found.
findfont: Font family 'Times New Roman' not found.
findfont: Font family 'Times New Roman' not found.
findfont: Font family 'Times New Roman' not found.
findfont: Font family 'Times New Roman' not found.
findfont: Font family 'Times New Roman' not found.
findfont: Font family 'Times New Roman' not found.
findfont: Font family 'Times New Roman' not found.
findfont: Font family 'Times New Roman' not found.
findfont: Font family 'Times New Roman' not found.
findfont: Font family 'Times New Roman' not found.
findfont: Font family 'Times New Roman' not found.
findfont: Font family 'Times New Roman' not found.
findfont: Font family 'Times New Roman' not found.
findfont: Font family 'Times New Roman' not found.
findfont: Font family 'Times New Roman' not found.
findfont: Font family 'Times New Roman' not found.
findfont: Font family 'Times New Roman' not found.
findfont: Font family 'Times New Roman' not found.
findfont: Font family 'Times New Roman' not found.
findfont: Font family 'Times New Roman' not found.
findfont: Font family 'Times New Roman' not found.
findfont: Font family 'Times New Roman' not found.
findfont: Font family 'Times New Roman' not found.
findfont: Font family 'Times New Roman' not found.
findfont: Font family 'Times New Roman' not found.
findfont: Font family 'Times New Roman' not found.
findfont: Font family 'Times New Roman' not found.
findfont: Font family 'Times New Roman' not found.
findfont: Font family 'Times New Roman' not found.
findfont: Font family 'Times New Roman' not found.
findfont: Font family 'Times New Roman' not found.
findfont: Font family 'Times New Roman' not found.
findfont: Font family 'Times New Roman' not found.
findfont: Font family 'Times New Roman' not found.
findfont: Font family 'Times New Roman' not found.
findfont: Font family 'Times New Roman' not found.
findfont: Font family 'Times New Roman' not found.
findfont: Font family 'Times New Roman' not found.
findfont: Font family 'Times New Roman' not found.
findfont: Font family 'Times New Roman' not found.
findfont: Font family 'Times New Roman' not found.
findfont: Font family 'Times New Roman' not found.
findfont: Font family 'Times New Roman' not found.
findfont: Font family 'Times New Roman' not found.
findfont: Font family 'Times New Roman' not found.
findfont: Font family 'Times New Roman' not found.
findfont: Font family 'Times New Roman' not found.
findfont: Font family 'Times New Roman' not found.
findfont: Font family 'Times New Roman' not found.
findfont: Font family 'Times New Roman' not found.
findfont: Font family 'Times New Roman' not found.
findfont: Font family 'Times New Roman' not found.
findfont: Font family 'Times New Roman' not found.
findfont: Font family 'Times New Roman' not found.
findfont: Font family 'Times New Roman' not found.
findfont: Font family 'Times New Roman' not found.
findfont: Font family 'Times New Roman' not found.
findfont: Font family 'Times New Roman' not found.
findfont: Font family 'Times New Roman' not found.
findfont: Font family 'Times New Roman' not found.
findfont: Font family 'Times New Roman' not found.
findfont: Font family 'Times New Roman' not found.
findfont: Font family 'Times New Roman' not found.
findfont: Font family 'Times New Roman' not found.
findfont: Font family 'Times New Roman' not found.
findfont: Font family 'Times New Roman' not found.
findfont: Font family 'Times New Roman' not found.
findfont: Font family 'Times New Roman' not found.
findfont: Font family 'Times New Roman' not found.
findfont: Font family 'Times New Roman' not found.
findfont: Font family 'Times New Roman' not found.
findfont: Font family 'Times New Roman' not found.
findfont: Font family 'Times New Roman' not found.
findfont: Font family 'Times New Roman' not found.
findfont: Font family 'Times New Roman' not found.
findfont: Font family 'Times New Roman' not found.
findfont: Font family 'Times New Roman' not found.
findfont: Font family 'Times New Roman' not found.
findfont: Font family 'Times New Roman' not found.
findfont: Font family 'Times New Roman' not found.
findfont: Font family 'Times New Roman' not found.
findfont: Font family 'Times New Roman' not found.
findfont: Font family 'Times New Roman' not found.
findfont: Font family 'Times New Roman' not found.
findfont: Font family 'Times New Roman' not found.
findfont: Font family 'Times New Roman' not found.
findfont: Font family 'Times New Roman' not found.
findfont: Font family 'Times New Roman' not found.
findfont: Font family 'Times New Roman' not found.
findfont: Font family 'Times New Roman' not found.
findfont: Font family 'Times New Roman' not found.
findfont: Font family 'Times New Roman' not found.
findfont: Font family 'Times New Roman' not found.
findfont: Font family 'Times New Roman' not found.
findfont: Font family 'Times New Roman' not found.
findfont: Font family 'Times New Roman' not found.
findfont: Font family 'Times New Roman' not found.
findfont: Font family 'Times New Roman' not found.
findfont: Font family 'Times New Roman' not found.
findfont: Font family 'Times New Roman' not found.
findfont: Font family 'Times New Roman' not found.
findfont: Font family 'Times New Roman' not found.
findfont: Font family 'Times New Roman' not found.
findfont: Font family 'Times New Roman' not found.
findfont: Font family 'Times New Roman' not found.
findfont: Font family 'Times New Roman' not found.
findfont: Font family 'Times New Roman' not found.
findfont: Font family 'Times New Roman' not found.
findfont: Font family 'Times New Roman' not found.
findfont: Font family 'Times New Roman' not found.
findfont: Font family 'Times New Roman' not found.
findfont: Font family 'Times New Roman' not found.
findfont: Font family 'Times New Roman' not found.
findfont: Font family 'Times New Roman' not found.
findfont: Font family 'Times New Roman' not found.
findfont: Font family 'Times New Roman' not found.
findfont: Font family 'Times New Roman' not found.
findfont: Font family 'Times New Roman' not found.
findfont: Font family 'Times New Roman' not found.
findfont: Font family 'Times New Roman' not found.
findfont: Font family 'Times New Roman' not found.
findfont: Font family 'Times New Roman' not found.
findfont: Font family 'Times New Roman' not found.
findfont: Font family 'Times New Roman' not found.
findfont: Font family 'Times New Roman' not found.
findfont: Font family 'Times New Roman' not found.
findfont: Font family 'Times New Roman' not found.
findfont: Font family 'Times New Roman' not found.
findfont: Font family 'Times New Roman' not found.
findfont: Font family 'Times New Roman' not found.
findfont: Font family 'Times New Roman' not found.
findfont: Font family 'Times New Roman' not found.
findfont: Font family 'Times New Roman' not found.
findfont: Font family 'Times New Roman' not found.
findfont: Font family 'Times New Roman' not found.
findfont: Font family 'Times New Roman' not found.
findfont: Font family 'Times New Roman' not found.
findfont: Font family 'Times New Roman' not found.
findfont: Font family 'Times New Roman' not found.
findfont: Font family 'Times New Roman' not found.
findfont: Font family 'Times New Roman' not found.
findfont: Font family 'Times New Roman' not found.
findfont: Font family 'Times New Roman' not found.
findfont: Font family 'Times New Roman' not found.
findfont: Font family 'Times New Roman' not found.
findfont: Font family 'Times New Roman' not found.
findfont: Font family 'Times New Roman' not found.
findfont: Font family 'Times New Roman' not found.
findfont: Font family 'Times New Roman' not found.
findfont: Font family 'Times New Roman' not found.
findfont: Font family 'Times New Roman' not found.
findfont: Font family 'Times New Roman' not found.
findfont: Font family 'Times New Roman' not found.
findfont: Font family 'Times New Roman' not found.
findfont: Font family 'Times New Roman' not found.
findfont: Font family 'Times New Roman' not found.
findfont: Font family 'Times New Roman' not found.
findfont: Font family 'Times New Roman' not found.
findfont: Font family 'Times New Roman' not found.
findfont: Font family 'Times New Roman' not found.
findfont: Font family 'Times New Roman' not found.
findfont: Font family 'Times New Roman' not found.
findfont: Font family 'Times New Roman' not found.
findfont: Font family 'Times New Roman' not found.
findfont: Font family 'Times New Roman' not found.
findfont: Font family 'Times New Roman' not found.
findfont: Font family 'Times New Roman' not found.
findfont: Font family 'Times New Roman' not found.
findfont: Font family 'Times New Roman' not found.
findfont: Font family 'Times New Roman' not found.
findfont: Font family 'Times New Roman' not found.
findfont: Font family 'Times New Roman' not found.
findfont: Font family 'Times New Roman' not found.
findfont: Font family 'Times New Roman' not found.
```

```
findfont: Font family 'Times New Roman' not found.
findfont: Font family 'Times New Roman' not found.
findfont: Font family 'Times New Roman' not found.
findfont: Font family 'Times New Roman' not found.
findfont: Font family 'Times New Roman' not found.
findfont: Font family 'Times New Roman' not found.
findfont: Font family 'Times New Roman' not found.
findfont: Font family 'Times New Roman' not found.
findfont: Font family 'Times New Roman' not found.
findfont: Font family 'Times New Roman' not found.
findfont: Font family 'Times New Roman' not found.
findfont: Font family 'Times New Roman' not found.
findfont: Font family 'Times New Roman' not found.
findfont: Font family 'Times New Roman' not found.
findfont: Font family 'Times New Roman' not found.
findfont: Font family 'Times New Roman' not found.
findfont: Font family 'Times New Roman' not found.
findfont: Font family 'Times New Roman' not found.
findfont: Font family 'Times New Roman' not found.
findfont: Font family 'Times New Roman' not found.
findfont: Font family 'Times New Roman' not found.
findfont: Font family 'Times New Roman' not found.
findfont: Font family 'Times New Roman' not found.
findfont: Font family 'Times New Roman' not found.
findfont: Font family 'Times New Roman' not found.
findfont: Font family 'Times New Roman' not found.
findfont: Font family 'Times New Roman' not found.
findfont: Font family 'Times New Roman' not found.
findfont: Font family 'Times New Roman' not found.
findfont: Font family 'Times New Roman' not found.
findfont: Font family 'Times New Roman' not found.
findfont: Font family 'Times New Roman' not found.
findfont: Font family 'Times New Roman' not found.
findfont: Font family 'Times New Roman' not found.
findfont: Font family 'Times New Roman' not found.
findfont: Font family 'Times New Roman' not found.
findfont: Font family 'Times New Roman' not found.
findfont: Font family 'Times New Roman' not found.
findfont: Font family 'Times New Roman' not found.
findfont: Font family 'Times New Roman' not found.
findfont: Font family 'Times New Roman' not found.
findfont: Font family 'Times New Roman' not found.
findfont: Font family 'Times New Roman' not found.
findfont: Font family 'Times New Roman' not found.
findfont: Font family 'Times New Roman' not found.
findfont: Font family 'Times New Roman' not found.
findfont: Font family 'Times New Roman' not found.
findfont: Font family 'Times New Roman' not found.
findfont: Font family 'Times New Roman' not found.
findfont: Font family 'Times New Roman' not found.
findfont: Font family 'Times New Roman' not found.
findfont: Font family 'Times New Roman' not found.
findfont: Font family 'Times New Roman' not found.
findfont: Font family 'Times New Roman' not found.
findfont: Font family 'Times New Roman' not found.
findfont: Font family 'Times New Roman' not found.
findfont: Font family 'Times New Roman' not found.
findfont: Font family 'Times New Roman' not found.
findfont: Font family 'Times New Roman' not found.
findfont: Font family 'Times New Roman' not found.
findfont: Font family 'Times New Roman' not found.
findfont: Font family 'Times New Roman' not found.
findfont: Font family 'Times New Roman' not found.
findfont: Font family 'Times New Roman' not found.
findfont: Font family 'Times New Roman' not found.
findfont: Font family 'Times New Roman' not found.
findfont: Font family 'Times New Roman' not found.
findfont: Font family 'Times New Roman' not found.
findfont: Font family 'Times New Roman' not found.
findfont: Font family 'Times New Roman' not found.
findfont: Font family 'Times New Roman' not found.
findfont: Font family 'Times New Roman' not found.
findfont: Font family 'Times New Roman' not found.
findfont: Font family 'Times New Roman' not found.
findfont: Font family 'Times New Roman' not found.
findfont: Font family 'Times New Roman' not found.
findfont: Font family 'Times New Roman' not found.
findfont: Font family 'Times New Roman' not found.
findfont: Font family 'Times New Roman' not found.
findfont: Font family 'Times New Roman' not found.
findfont: Font family 'Times New Roman' not found.
findfont: Font family 'Times New Roman' not found.
findfont: Font family 'Times New Roman' not found.
findfont: Font family 'Times New Roman' not found.
findfont: Font family 'Times New Roman' not found.
findfont: Font family 'Times New Roman' not found.
findfont: Font family 'Times New Roman' not found.
findfont: Font family 'Times New Roman' not found.
findfont: Font family 'Times New Roman' not found.
findfont: Font family 'Times New Roman' not found.
findfont: Font family 'Times New Roman' not found.
findfont: Font family 'Times New Roman' not found.
findfont: Font family 'Times New Roman' not found.
findfont: Font family 'Times New Roman' not found.
findfont: Font family 'Times New Roman' not found.
findfont: Font family 'Times New Roman' not found.
findfont: Font family 'Times New Roman' not found.
findfont: Font family 'Times New Roman' not found.
findfont: Font family 'Times New Roman' not found.
findfont: Font family 'Times New Roman' not found.
findfont: Font family 'Times New Roman' not found.
findfont: Font family 'Times New Roman' not found.
findfont: Font family 'Times New Roman' not found.
findfont: Font family 'Times New Roman' not found.
findfont: Font family 'Times New Roman' not found.
findfont: Font family 'Times New Roman' not found.
findfont: Font family 'Times New Roman' not found.
findfont: Font family 'Times New Roman' not found.
findfont: Font family 'Times New Roman' not found.
findfont: Font family 'Times New Roman' not found.
findfont: Font family 'Times New Roman' not found.
findfont: Font family 'Times New Roman' not found.
findfont: Font family 'Times New Roman' not found.
findfont: Font family 'Times New Roman' not found.
findfont: Font family 'Times New Roman' not found.
findfont: Font family 'Times New Roman' not found.
findfont: Font family 'Times New Roman' not found.
findfont: Font family 'Times New Roman' not found.
findfont: Font family 'Times New Roman' not found.
findfont: Font family 'Times New Roman' not found.
findfont: Font family 'Times New Roman' not found.
findfont: Font family 'Times New Roman' not found.
findfont: Font family 'Times New Roman' not found.
findfont: Font family 'Times New Roman' not found.
findfont: Font family 'Times New Roman' not found.
findfont: Font family 'Times New Roman' not found.
findfont: Font family 'Times New Roman' not found.
findfont: Font family 'Times New Roman' not found.
findfont: Font family 'Times New Roman' not found.
findfont: Font family 'Times New Roman' not found.
findfont: Font family 'Times New Roman' not found.
findfont: Font family 'Times New Roman' not found.
findfont: Font family 'Times New Roman' not found.
findfont: Font family 'Times New Roman' not found.
findfont: Font family 'Times New Roman' not found.
findfont: Font family 'Times New Roman' not found.
findfont: Font family 'Times New Roman' not found.
findfont: Font family 'Times New Roman' not found.
findfont: Font family 'Times New Roman' not found.
findfont: Font family 'Times New Roman' not found.
findfont: Font family 'Times New Roman' not found.
findfont: Font family 'Times New Roman' not found.
findfont: Font family 'Times New Roman' not found.
findfont: Font family 'Times New Roman' not found.
findfont: Font family 'Times New Roman' not found.
findfont: Font family 'Times New Roman' not found.
findfont: Font family 'Times New Roman' not found.
findfont: Font family 'Times New Roman' not found.
findfont: Font family 'Times New Roman' not found.
findfont: Font family 'Times New Roman' not found.
findfont: Font family 'Times New Roman' not found.
findfont: Font family 'Times New Roman' not found.
findfont: Font family 'Times New Roman' not found.
findfont: Font family 'Times New Roman' not found.
findfont: Font family 'Times New Roman' not found.
findfont: Font family 'Times New Roman' not found.
findfont: Font family 'Times New Roman' not found.
findfont: Font family 'Times New Roman' not found.
findfont: Font family 'Times New Roman' not found.
findfont: Font family 'Times New Roman' not found.
findfont: Font family 'Times New Roman' not found.
findfont: Font family 'Times New Roman' not found.
findfont: Font family 'Times New Roman' not found.
findfont: Font family 'Times New Roman' not found.
findfont: Font family 'Times New Roman' not found.
findfont: Font family 'Times New Roman' not found.
findfont: Font family 'Times New Roman' not found.
findfont: Font family 'Times New Roman' not found.
findfont: Font family 'Times New Roman' not found.
findfont: Font family 'Times New Roman' not found.
findfont: Font family 'Times New Roman' not found.
findfont: Font family 'Times New Roman' not found.
findfont: Font family 'Times New Roman' not found.
findfont: Font family 'Times New Roman' not found.
findfont: Font family 'Times New Roman' not found.
findfont: Font family 'Times New Roman' not found.
findfont: Font family 'Times New Roman' not found.
findfont: Font family 'Times New Roman' not found.
findfont: Font family 'Times New Roman' not found.
findfont: Font family 'Times New Roman' not found.
findfont: Font family 'Times New Roman' not found.
findfont: Font family 'Times New Roman' not found.
findfont: Font family 'Times New Roman' not found.
findfont: Font family 'Times New Roman' not found.
findfont: Font family 'Times New Roman' not found.
findfont: Font family 'Times New Roman' not found.
findfont: Font family 'Times New Roman' not found.
findfont: Font family 'Times New Roman' not found.
findfont: Font family 'Times New Roman' not found.
findfont: Font family 'Times New Roman' not found.
findfont: Font family 'Times New Roman' not found.
findfont: Font family 'Times New Roman' not found.
findfont: Font family 'Times New Roman' not found.
findfont: Font family 'Times New Roman' not found.
findfont: Font family 'Times New Roman' not found.
findfont: Font family 'Times New Roman' not found.
findfont: Font family 'Times New Roman' not found.
findfont: Font family 'Times New Roman' not found.
findfont: Font family 'Times New Roman' not found.
findfont: Font family 'Times New Roman' not found.
findfont: Font family 'Times New Roman' not found.
findfont: Font family 'Times New Roman' not found.
findfont: Font family 'Times New Roman' not found.
findfont: Font family 'Times New Roman' not found.
findfont: Font family 'Times New Roman' not found.
findfont: Font family 'Times New Roman' not found.
findfont: Font family 'Times New Roman' not found.
findfont: Font family 'Times New Roman' not found.
findfont: Font family 'Times New Roman' not found.
findfont: Font family 'Times New Roman' not found.
findfont: Font family 'Times New Roman' not found.
findfont: Font family 'Times New Roman' not found.
findfont: Font family 'Times New Roman' not found.
findfont: Font family 'Times New Roman' not found.
findfont: Font family 'Times New Roman' not found.
findfont: Font family 'Times New Roman' not found.
findfont: Font family 'Times New Roman' not found.
findfont: Font family 'Times New Roman' not found.
findfont: Font family 'Times New Roman' not found.
findfont: Font family 'Times New Roman' not found.
findfont: Font family 'Times New Roman' not found.
findfont: Font family 'Times New Roman' not found.
findfont: Font family 'Times New Roman' not found.
findfont: Font family 'Times New Roman' not found.
findfont: Font family 'Times New Roman' not found.
findfont: Font family 'Times New Roman' not found.
findfont: Font family 'Times New Roman' not found.
findfont: Font family 'Times New Roman' not found.
findfont: Font family 'Times New Roman' not found.
findfont: Font family 'Times New Roman' not found.
findfont: Font family 'Times New Roman' not found.
findfont: Font family 'Times New Roman' not found.
findfont: Font family 'Times New Roman' not found.
findfont: Font family 'Times New Roman' not found.
findfont: Font family 'Times New Roman' not found.
findfont: Font family 'Times New Roman' not found.
findfont: Font family 'Times New Roman' not found.
findfont: Font family 'Times New Roman' not found.
findfont: Font family 'Times New Roman' not found.
findfont: Font family 'Times New Roman' not found.
findfont: Font family 'Times New Roman' not found.
findfont: Font family 'Times New Roman' not found.
findfont: Font family 'Times New Roman' not found.
findfont: Font family 'Times New Roman' not found.
findfont: Font family 'Times New Roman' not found.
findfont: Font family 'Times New Roman' not found.
findfont: Font family 'Times New Roman' not found.
findfont: Font family 'Times New Roman' not found.
findfont: Font family 'Times New Roman' not found.
findfont: Font family 'Times New Roman' not found.
findfont: Font family 'Times New Roman' not found.
findfont: Font family 'Times New Roman' not found.
findfont: Font family 'Times New Roman' not found.
findfont: Font family 'Times New Roman' not found.
findfont: Font family 'Times New Roman' not found.
findfont: Font family 'Times New Roman' not found.
findfont: Font family 'Times New Roman' not found.
findfont: Font family 'Times New Roman' not found.
findfont: Font family 'Times New Roman' not found.
findfont: Font family 'Times New Roman' not found.
findfont: Font family 'Times New Roman' not found.
findfont: Font family 'Times New Roman' not found.
findfont: Font family 'Times New Roman' not found.
findfont: Font family 'Times New Roman' not found.
findfont: Font family 'Times New Roman' not found.
findfont: Font family 'Times New Roman' not found.
findfont: Font family 'Times New Roman' not found.
findfont: Font family 'Times New Roman' not found.
findfont: Font family 'Times New Roman' not found.
findfont: Font family 'Times New Roman' not found.
findfont: Font family 'Times New Roman' not found.
findfont: Font family 'Times New Roman' not found.
findfont: Font family 'Times New Roman' not found.
findfont: Font family 'Times New Roman' not found.
findfont: Font family 'Times New Roman' not found.
findfont: Font family 'Times New Roman' not found.
findfont: Font family 'Times New Roman' not found.
findfont: Font family 'Times New Roman' not found.
findfont: Font family 'Times New Roman' not found.
findfont: Font family 'Times New Roman' not found.
findfont: Font family 'Times New Roman' not found.
findfont: Font family 'Times New Roman' not found.
findfont: Font family 'Times New Roman' not found.
findfont: Font family 'Times New Roman' not found.
findfont: Font family 'Times New Roman' not found.
findfont: Font family 'Times New Roman' not found.
findfont: Font family 'Times New Roman' not found.
findfont: Font family 'Times New Roman' not found.
findfont: Font family 'Times New Roman' not found.
findfont: Font family 'Times New Roman' not found.
findfont: Font family 'Times New Roman' not found.
findfont: Font family 'Times New Roman' not found.
findfont: Font family 'Times New Roman' not found.
findfont: Font family 'Times New Roman' not found.
findfont: Font family 'Times New Roman' not found.
findfont: Font family 'Times New Roman' not found.
findfont: Font family 'Times New Roman' not found.
findfont: Font family 'Times New Roman' not found.
findfont: Font family 'Times New Roman' not found.
findfont: Font family 'Times New Roman' not found.
findfont: Font family 'Times New Roman' not found.
findfont: Font family 'Times New Roman' not found.
findfont: Font family 'Times New Roman' not found.
findfont: Font family 'Times New Roman' not found.
findfont: Font family 'Times New Roman' not found.
findfont: Font family 'Times New Roman' not found.
```

```
findfont: Font family 'Times New Roman' not found.
findfont: Font family 'Times New Roman' not found.
findfont: Font family 'Times New Roman' not found.
findfont: Font family 'Times New Roman' not found.
findfont: Font family 'Times New Roman' not found.
findfont: Font family 'Times New Roman' not found.
findfont: Font family 'Times New Roman' not found.
findfont: Font family 'Times New Roman' not found.
findfont: Font family 'Times New Roman' not found.
findfont: Font family 'Times New Roman' not found.
findfont: Font family 'Times New Roman' not found.
findfont: Font family 'Times New Roman' not found.
findfont: Font family 'Times New Roman' not found.
findfont: Font family 'Times New Roman' not found.
findfont: Font family 'Times New Roman' not found.
findfont: Font family 'Times New Roman' not found.
findfont: Font family 'Times New Roman' not found.
findfont: Font family 'Times New Roman' not found.
findfont: Font family 'Times New Roman' not found.
findfont: Font family 'Times New Roman' not found.
findfont: Font family 'Times New Roman' not found.
findfont: Font family 'Times New Roman' not found.
findfont: Font family 'Times New Roman' not found.
findfont: Font family 'Times New Roman' not found.
findfont: Font family 'Times New Roman' not found.
findfont: Font family 'Times New Roman' not found.
findfont: Font family 'Times New Roman' not found.
findfont: Font family 'Times New Roman' not found.
findfont: Font family 'Times New Roman' not found.
findfont: Font family 'Times New Roman' not found.
findfont: Font family 'Times New Roman' not found.
findfont: Font family 'Times New Roman' not found.
findfont: Font family 'Times New Roman' not found.
findfont: Font family 'Times New Roman' not found.
findfont: Font family 'Times New Roman' not found.
findfont: Font family 'Times New Roman' not found.
findfont: Font family 'Times New Roman' not found.
findfont: Font family 'Times New Roman' not found.
findfont: Font family 'Times New Roman' not found.
findfont: Font family 'Times New Roman' not found.
findfont: Font family 'Times New Roman' not found.
findfont: Font family 'Times New Roman' not found.
findfont: Font family 'Times New Roman' not found.
findfont: Font family 'Times New Roman' not found.
findfont: Font family 'Times New Roman' not found.
findfont: Font family 'Times New Roman' not found.
findfont: Font family 'Times New Roman' not found.
findfont: Font family 'Times New Roman' not found.
findfont: Font family 'Times New Roman' not found.
findfont: Font family 'Times New Roman' not found.
findfont: Font family 'Times New Roman' not found.
findfont: Font family 'Times New Roman' not found.
findfont: Font family 'Times New Roman' not found.
findfont: Font family 'Times New Roman' not found.
findfont: Font family 'Times New Roman' not found.
findfont: Font family 'Times New Roman' not found.
findfont: Font family 'Times New Roman' not found.
findfont: Font family 'Times New Roman' not found.
findfont: Font family 'Times New Roman' not found.
findfont: Font family 'Times New Roman' not found.
findfont: Font family 'Times New Roman' not found.
findfont: Font family 'Times New Roman' not found.
findfont: Font family 'Times New Roman' not found.
findfont: Font family 'Times New Roman' not found.
findfont: Font family 'Times New Roman' not found.
findfont: Font family 'Times New Roman' not found.
findfont: Font family 'Times New Roman' not found.
findfont: Font family 'Times New Roman' not found.
findfont: Font family 'Times New Roman' not found.
findfont: Font family 'Times New Roman' not found.
findfont: Font family 'Times New Roman' not found.
findfont: Font family 'Times New Roman' not found.
findfont: Font family 'Times New Roman' not found.
findfont: Font family 'Times New Roman' not found.
findfont: Font family 'Times New Roman' not found.
findfont: Font family 'Times New Roman' not found.
findfont: Font family 'Times New Roman' not found.
findfont: Font family 'Times New Roman' not found.
findfont: Font family 'Times New Roman' not found.
findfont: Font family 'Times New Roman' not found.
findfont: Font family 'Times New Roman' not found.
findfont: Font family 'Times New Roman' not found.
findfont: Font family 'Times New Roman' not found.
findfont: Font family 'Times New Roman' not found.
findfont: Font family 'Times New Roman' not found.
findfont: Font family 'Times New Roman' not found.
findfont: Font family 'Times New Roman' not found.
findfont: Font family 'Times New Roman' not found.
findfont: Font family 'Times New Roman' not found.
findfont: Font family 'Times New Roman' not found.
findfont: Font family 'Times New Roman' not found.
findfont: Font family 'Times New Roman' not found.
findfont: Font family 'Times New Roman' not found.
findfont: Font family 'Times New Roman' not found.
findfont: Font family 'Times New Roman' not found.
findfont: Font family 'Times New Roman' not found.
findfont: Font family 'Times New Roman' not found.
findfont: Font family 'Times New Roman' not found.
findfont: Font family 'Times New Roman' not found.
findfont: Font family 'Times New Roman' not found.
findfont: Font family 'Times New Roman' not found.
findfont: Font family 'Times New Roman' not found.
findfont: Font family 'Times New Roman' not found.
findfont: Font family 'Times New Roman' not found.
findfont: Font family 'Times New Roman' not found.
findfont: Font family 'Times New Roman' not found.
findfont: Font family 'Times New Roman' not found.
findfont: Font family 'Times New Roman' not found.
findfont: Font family 'Times New Roman' not found.
findfont: Font family 'Times New Roman' not found.
findfont: Font family 'Times New Roman' not found.
findfont: Font family 'Times New Roman' not found.
findfont: Font family 'Times New Roman' not found.
findfont: Font family 'Times New Roman' not found.
findfont: Font family 'Times New Roman' not found.
findfont: Font family 'Times New Roman' not found.
findfont: Font family 'Times New Roman' not found.
findfont: Font family 'Times New Roman' not found.
findfont: Font family 'Times New Roman' not found.
findfont: Font family 'Times New Roman' not found.
findfont: Font family 'Times New Roman' not found.
findfont: Font family 'Times New Roman' not found.
findfont: Font family 'Times New Roman' not found.
findfont: Font family 'Times New Roman' not found.
findfont: Font family 'Times New Roman' not found.
findfont: Font family 'Times New Roman' not found.
findfont: Font family 'Times New Roman' not found.
findfont: Font family 'Times New Roman' not found.
findfont: Font family 'Times New Roman' not found.
findfont: Font family 'Times New Roman' not found.
findfont: Font family 'Times New Roman' not found.
findfont: Font family 'Times New Roman' not found.
findfont: Font family 'Times New Roman' not found.
findfont: Font family 'Times New Roman' not found.
findfont: Font family 'Times New Roman' not found.
findfont: Font family 'Times New Roman' not found.
findfont: Font family 'Times New Roman' not found.
findfont: Font family 'Times New Roman' not found.
findfont: Font family 'Times New Roman' not found.
findfont: Font family 'Times New Roman' not found.
findfont: Font family 'Times New Roman' not found.
findfont: Font family 'Times New Roman' not found.
findfont: Font family 'Times New Roman' not found.
findfont: Font family 'Times New Roman' not found.
findfont: Font family 'Times New Roman' not found.
findfont: Font family 'Times New Roman' not found.
findfont: Font family 'Times New Roman' not found.
findfont: Font family 'Times New Roman' not found.
findfont: Font family 'Times New Roman' not found.
findfont: Font family 'Times New Roman' not found.
findfont: Font family 'Times New Roman' not found.
findfont: Font family 'Times New Roman' not found.
findfont: Font family 'Times New Roman' not found.
findfont: Font family 'Times New Roman' not found.
findfont: Font family 'Times New Roman' not found.
findfont: Font family 'Times New Roman' not found.
findfont: Font family 'Times New Roman' not found.
findfont: Font family 'Times New Roman' not found.
findfont: Font family 'Times New Roman' not found.
findfont: Font family 'Times New Roman' not found.
findfont: Font family 'Times New Roman' not found.
findfont: Font family 'Times New Roman' not found.
findfont: Font family 'Times New Roman' not found.
findfont: Font family 'Times New Roman' not found.
findfont: Font family 'Times New Roman' not found.
findfont: Font family 'Times New Roman' not found.
findfont: Font family 'Times New Roman' not found.
findfont: Font family 'Times New Roman' not found.
findfont: Font family 'Times New Roman' not found.
findfont: Font family 'Times New Roman' not found.
findfont: Font family 'Times New Roman' not found.
findfont: Font family 'Times New Roman' not found.
findfont: Font family 'Times New Roman' not found.
findfont: Font family 'Times New Roman' not found.
findfont: Font family 'Times New Roman' not found.
findfont: Font family 'Times New Roman' not found.
findfont: Font family 'Times New Roman' not found.
findfont: Font family 'Times New Roman' not found.
findfont: Font family 'Times New Roman' not found.
findfont: Font family 'Times New Roman' not found.
findfont: Font family 'Times New Roman' not found.
findfont: Font family 'Times New Roman' not found.
findfont: Font family 'Times New Roman' not found.
findfont: Font family 'Times New Roman' not found.
findfont: Font family 'Times New Roman' not found.
findfont: Font family 'Times New Roman' not found.
findfont: Font family 'Times New Roman' not found.
findfont: Font family 'Times New Roman' not found.
findfont: Font family 'Times New Roman' not found.
findfont: Font family 'Times New Roman' not found.
findfont: Font family 'Times New Roman' not found.
findfont: Font family 'Times New Roman' not found.
findfont: Font family 'Times New Roman' not found.
findfont: Font family 'Times New Roman' not found.
findfont: Font family 'Times New Roman' not found.
findfont: Font family 'Times New Roman' not found.
findfont: Font family 'Times New Roman' not found.
findfont: Font family 'Times New Roman' not found.
findfont: Font family 'Times New Roman' not found.
findfont: Font family 'Times New Roman' not found.
findfont: Font family 'Times New Roman' not found.
findfont: Font family 'Times New Roman' not found.
findfont: Font family 'Times New Roman' not found.
findfont: Font family 'Times New Roman' not found.
findfont: Font family 'Times New Roman' not found.
findfont: Font family 'Times New Roman' not found.
findfont: Font family 'Times New Roman' not found.
findfont: Font family 'Times New Roman' not found.
findfont: Font family 'Times New Roman' not found.
findfont: Font family 'Times New Roman' not found.
findfont: Font family 'Times New Roman' not found.
findfont: Font family 'Times New Roman' not found.
findfont: Font family 'Times New Roman' not found.
findfont: Font family 'Times New Roman' not found.
findfont: Font family 'Times New Roman' not found.
findfont: Font family 'Times New Roman' not found.
findfont: Font family 'Times New Roman' not found.
findfont: Font family 'Times New Roman' not found.
findfont: Font family 'Times New Roman' not found.
findfont: Font family 'Times New Roman' not found.
findfont: Font family 'Times New Roman' not found.
findfont: Font family 'Times New Roman' not found.
findfont: Font family 'Times New Roman' not found.
findfont: Font family 'Times New Roman' not found.
findfont: Font family 'Times New Roman' not found.
findfont: Font family 'Times New Roman' not found.
findfont: Font family 'Times New Roman' not found.
findfont: Font family 'Times New Roman' not found.
findfont: Font family 'Times New Roman' not found.
findfont: Font family 'Times New Roman' not found.
findfont: Font family 'Times New Roman' not found.
findfont: Font family 'Times New Roman' not found.
findfont: Font family 'Times New Roman' not found.
findfont: Font family 'Times New Roman' not found.
findfont: Font family 'Times New Roman' not found.
findfont: Font family 'Times New Roman' not found.
findfont: Font family 'Times New Roman' not found.
findfont: Font family 'Times New Roman' not found.
findfont: Font family 'Times New Roman' not found.
findfont: Font family 'Times New Roman' not found.
findfont: Font family 'Times New Roman' not found.
findfont: Font family 'Times New Roman' not found.
findfont: Font family 'Times New Roman' not found.
findfont: Font family 'Times New Roman' not found.
findfont: Font family 'Times New Roman' not found.
findfont: Font family 'Times New Roman' not found.
findfont: Font family 'Times New Roman' not found.
findfont: Font family 'Times New Roman' not found.
findfont: Font family 'Times New Roman' not found.
findfont: Font family 'Times New Roman' not found.
findfont: Font family 'Times New Roman' not found.
findfont: Font family 'Times New Roman' not found.
findfont: Font family 'Times New Roman' not found.
findfont: Font family 'Times New Roman' not found.
findfont: Font family 'Times New Roman' not found.
findfont: Font family 'Times New Roman' not found.
findfont: Font family 'Times New Roman' not found.
findfont: Font family 'Times New Roman' not found.
findfont: Font family 'Times New Roman' not found.
findfont: Font family 'Times New Roman' not found.
findfont: Font family 'Times New Roman' not found.
findfont: Font family 'Times New Roman' not found.
findfont: Font family 'Times New Roman' not found.
findfont: Font family 'Times New Roman' not found.
findfont: Font family 'Times New Roman' not found.
findfont: Font family 'Times New Roman' not found.
findfont: Font family 'Times New Roman' not found.
findfont: Font family 'Times New Roman' not found.
findfont: Font family 'Times New Roman' not found.
findfont: Font family 'Times New Roman' not found.
findfont: Font family 'Times New Roman' not found.
findfont: Font family 'Times New Roman' not found.
findfont: Font family 'Times New Roman' not found.
findfont: Font family 'Times New Roman' not found.
findfont: Font family 'Times New Roman' not found.
findfont: Font family 'Times New Roman' not found.
findfont: Font family 'Times New Roman' not found.
findfont: Font family 'Times New Roman' not found.
findfont: Font family 'Times New Roman' not found.
findfont: Font family 'Times New Roman' not found.
findfont: Font family 'Times New Roman' not found.
findfont: Font family 'Times New Roman' not found.
findfont: Font family 'Times New Roman' not found.
findfont: Font family 'Times New Roman' not found.
findfont: Font family 'Times New Roman' not found.
findfont: Font family 'Times New Roman' not found.
findfont: Font family 'Times New Roman' not found.
findfont: Font family 'Times New Roman' not found.
findfont: Font family 'Times New Roman' not found.
findfont: Font family 'Times New Roman' not found.
findfont: Font family 'Times New Roman' not found.
findfont: Font family 'Times New Roman' not found.
findfont: Font family 'Times New Roman' not found.
findfont: Font family 'Times New Roman' not found.
findfont: Font family 'Times New Roman' not found.
findfont: Font family 'Times New Roman' not found.
findfont: Font family 'Times New Roman' not found.
findfont: Font family 'Times New Roman' not found.
findfont: Font family 'Times New Roman' not found.
findfont: Font family 'Times New Roman' not found.
findfont: Font family 'Times New Roman' not found.
findfont: Font family 'Times New Roman' not found.
findfont: Font family 'Times New Roman' not found.
findfont: Font family 'Times New Roman' not found.
findfont: Font family 'Times New Roman' not found.
findfont: Font family 'Times New Roman' not found.
findfont: Font family 'Times New Roman' not found.
findfont: Font family 'Times New Roman' not found.
findfont: Font family 'Times New Roman' not found.
findfont: Font family 'Times New Roman' not found.
```

```
Done! PDFs saved as: ROC_Test.pdf, ROC_Data1.pdf, ROC_Data2.pdf
```

In [8]:

```
import matplotlib.pyplot as plt

# 
plt.rcParams['font.family'] = 'sans-serif'
plt.rcParams['font.sans-serif'] = ['DejaVu Sans', 'Arial', 'Helvetica', 'Verdana']

# 
# plt.rcParams['font.sans-serif'] = ['SimHei', 'Microsoft YaHei']  # 
plt.rcParams['axes.unicode_minus'] = False  #
```

In [7]:

```
import os

work_dir = r"/Plot"
os.makedirs(work_dir, exist_ok=True)   # 
os.chdir(work_dir)                     # 

print("Current working directory:", os.getcwd())
```

```
Current working directory: /home/data/t050556/newR
```

In [ ]:

```

```
