# Supplementary figures and images for: Integrated Genomic and Single‐Cell Analysis Reveals Heterogeneity, Prognosis, and Treatment Vulnerability in Urothelial Carcinoma
Source: Hum Mutat. 2026 May 27;2026:2797474. doi: 10.1155/humu/2797474 (PMC13213714; doi:10.1155/humu/2797474)

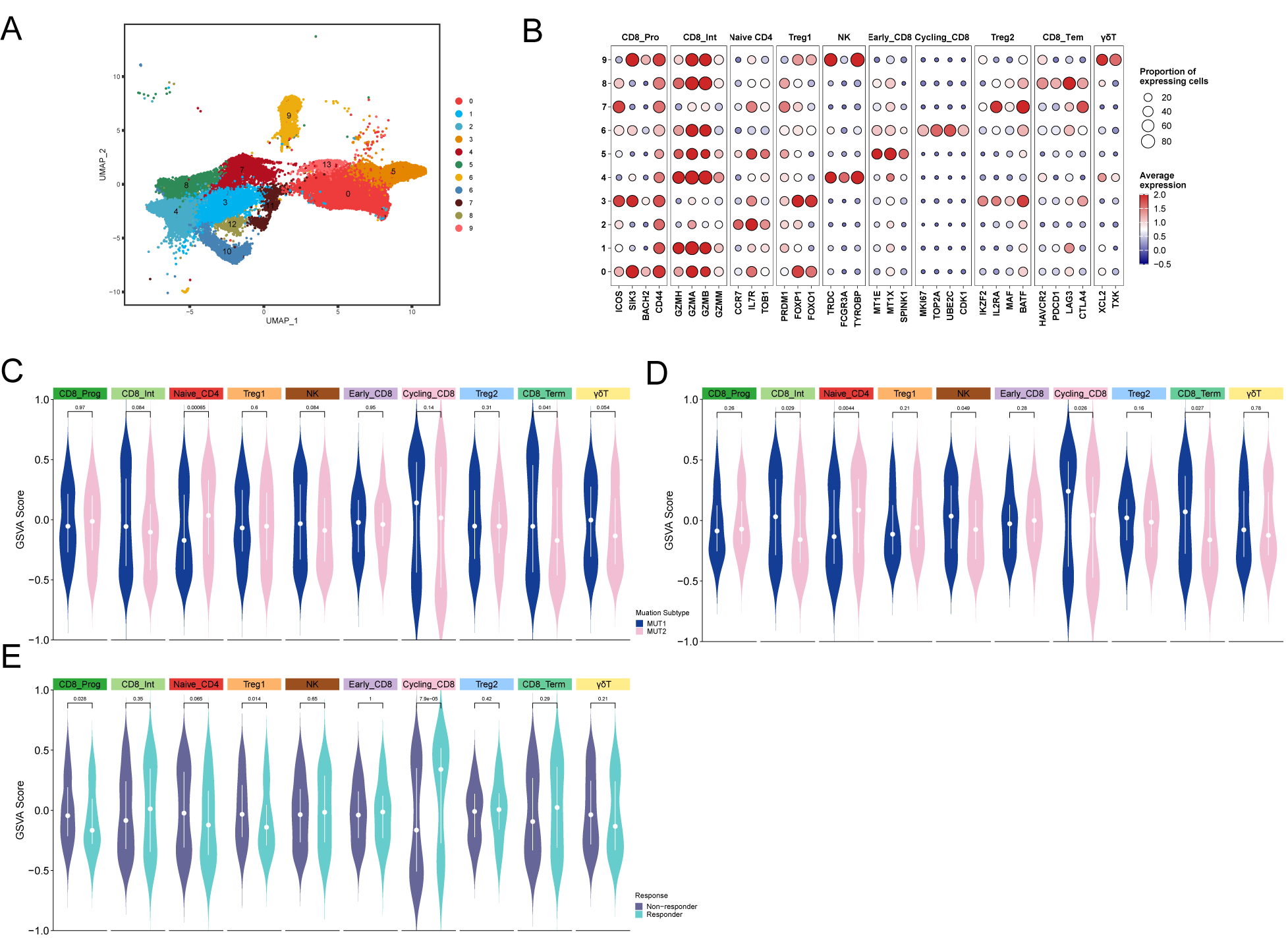

Supplement: Supplementary file 1 — Supporting Information Additional supporting information can be found online in the Supporting Information 1. Supporting Information. Methods S1: Computation and interpretation of cosine similarity. Methods S2: Identification of the mutational subtype of urothelial carcinoma. Methods S3: Identification of the risk score for urothelial carcinoma. Methods S4: Development and external validation of a machine learning–based prediction model. Supporting Information 2. Figure S1: Non‐negative matrix factorization (NMF) of the cosine similarity matrix of mutation signatures from TCGA cohort. Supporting Information 3. Figure S2: Identification and clinical characteristics of mutational signature classification in TCGA training cohort. Supporting Information 4. Figure S3: Clinical characteristics of mutational signature classifications in the MSK2022 test cohort. Supporting Information 5. Figure S4: Clinical characteristics according to the mutational signature classification in the MSK2015 test cohort. Supporting Information 6. Figure S5: Clinical characteristics of mutational signature classification in the IMvigor210 and UC‐GENOME test cohorts. Supporting Information 7. Figure S6: Kaplan–Meier curves depicting overall survival (OS) in patients stratified by the genomic mutation–based risk score: score < 1 (low risk) versus ≥ 1 (high risk). Supporting Information 8. Figure S7: Differences in clinical characteristics according to the mutation signature subtype and immunotherapy response. Supporting Information 9. Figure S8: Subgroup analysis of clinical characteristics based on the mutational subtype (IMvigor210 cohort). Supporting Information 10. Figure S9: Subgroup analysis of clinical characteristics based on the mutational subtype (UC‐GENOME cohort). Supporting Information 11. Figure S10: Univariate analysis for mutational signature subtype and clinical characteristics across multiple cohorts. Supporting Information 12. Figure S11: Multivariate analysis for mutational s [file HUMU-2026-2797474-s001.zip › Supplementary Figure 15.tif]

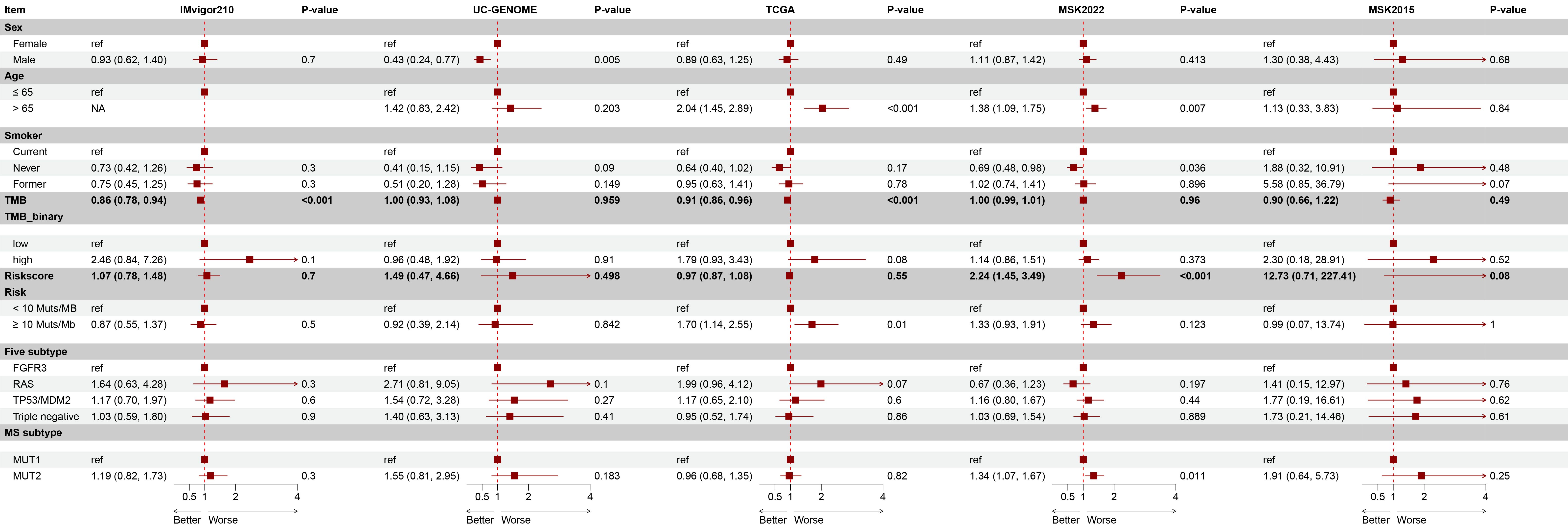

Supplement: Supplementary file 1 — Supporting Information Additional supporting information can be found online in the Supporting Information 1. Supporting Information. Methods S1: Computation and interpretation of cosine similarity. Methods S2: Identification of the mutational subtype of urothelial carcinoma. Methods S3: Identification of the risk score for urothelial carcinoma. Methods S4: Development and external validation of a machine learning–based prediction model. Supporting Information 2. Figure S1: Non‐negative matrix factorization (NMF) of the cosine similarity matrix of mutation signatures from TCGA cohort. Supporting Information 3. Figure S2: Identification and clinical characteristics of mutational signature classification in TCGA training cohort. Supporting Information 4. Figure S3: Clinical characteristics of mutational signature classifications in the MSK2022 test cohort. Supporting Information 5. Figure S4: Clinical characteristics according to the mutational signature classification in the MSK2015 test cohort. Supporting Information 6. Figure S5: Clinical characteristics of mutational signature classification in the IMvigor210 and UC‐GENOME test cohorts. Supporting Information 7. Figure S6: Kaplan–Meier curves depicting overall survival (OS) in patients stratified by the genomic mutation–based risk score: score < 1 (low risk) versus ≥ 1 (high risk). Supporting Information 8. Figure S7: Differences in clinical characteristics according to the mutation signature subtype and immunotherapy response. Supporting Information 9. Figure S8: Subgroup analysis of clinical characteristics based on the mutational subtype (IMvigor210 cohort). Supporting Information 10. Figure S9: Subgroup analysis of clinical characteristics based on the mutational subtype (UC‐GENOME cohort). Supporting Information 11. Figure S10: Univariate analysis for mutational signature subtype and clinical characteristics across multiple cohorts. Supporting Information 12. Figure S11: Multivariate analysis for mutational s [file HUMU-2026-2797474-s001.zip › Supplementary Figure 11.jpg]

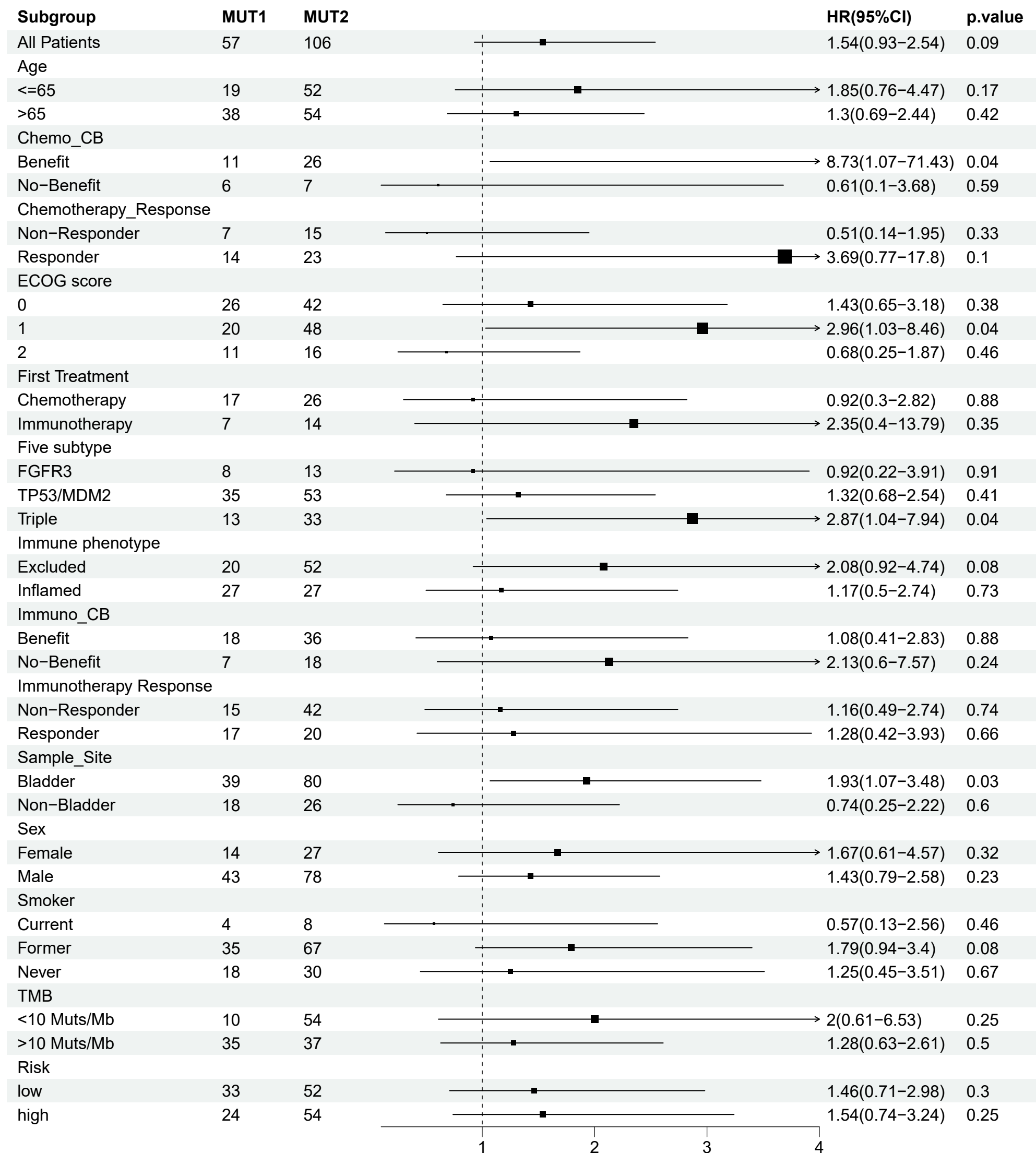

Supplement: Supplementary file 1 — Supporting Information Additional supporting information can be found online in the Supporting Information 1. Supporting Information. Methods S1: Computation and interpretation of cosine similarity. Methods S2: Identification of the mutational subtype of urothelial carcinoma. Methods S3: Identification of the risk score for urothelial carcinoma. Methods S4: Development and external validation of a machine learning–based prediction model. Supporting Information 2. Figure S1: Non‐negative matrix factorization (NMF) of the cosine similarity matrix of mutation signatures from TCGA cohort. Supporting Information 3. Figure S2: Identification and clinical characteristics of mutational signature classification in TCGA training cohort. Supporting Information 4. Figure S3: Clinical characteristics of mutational signature classifications in the MSK2022 test cohort. Supporting Information 5. Figure S4: Clinical characteristics according to the mutational signature classification in the MSK2015 test cohort. Supporting Information 6. Figure S5: Clinical characteristics of mutational signature classification in the IMvigor210 and UC‐GENOME test cohorts. Supporting Information 7. Figure S6: Kaplan–Meier curves depicting overall survival (OS) in patients stratified by the genomic mutation–based risk score: score < 1 (low risk) versus ≥ 1 (high risk). Supporting Information 8. Figure S7: Differences in clinical characteristics according to the mutation signature subtype and immunotherapy response. Supporting Information 9. Figure S8: Subgroup analysis of clinical characteristics based on the mutational subtype (IMvigor210 cohort). Supporting Information 10. Figure S9: Subgroup analysis of clinical characteristics based on the mutational subtype (UC‐GENOME cohort). Supporting Information 11. Figure S10: Univariate analysis for mutational signature subtype and clinical characteristics across multiple cohorts. Supporting Information 12. Figure S11: Multivariate analysis for mutational s [file HUMU-2026-2797474-s001.zip › Supplementary Figure 9.pdf]

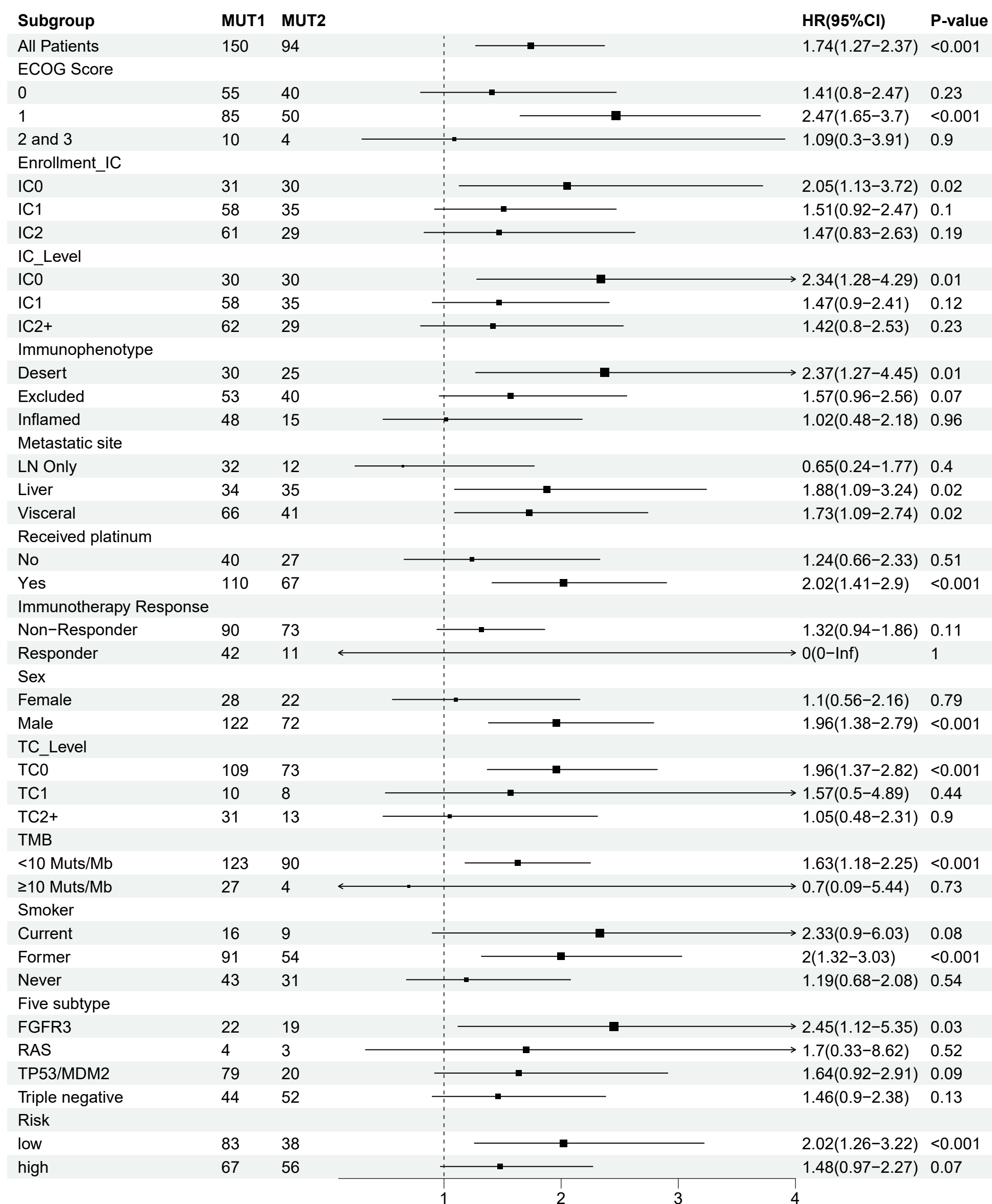

Supplement: Supplementary file 1 — Supporting Information Additional supporting information can be found online in the Supporting Information 1. Supporting Information. Methods S1: Computation and interpretation of cosine similarity. Methods S2: Identification of the mutational subtype of urothelial carcinoma. Methods S3: Identification of the risk score for urothelial carcinoma. Methods S4: Development and external validation of a machine learning–based prediction model. Supporting Information 2. Figure S1: Non‐negative matrix factorization (NMF) of the cosine similarity matrix of mutation signatures from TCGA cohort. Supporting Information 3. Figure S2: Identification and clinical characteristics of mutational signature classification in TCGA training cohort. Supporting Information 4. Figure S3: Clinical characteristics of mutational signature classifications in the MSK2022 test cohort. Supporting Information 5. Figure S4: Clinical characteristics according to the mutational signature classification in the MSK2015 test cohort. Supporting Information 6. Figure S5: Clinical characteristics of mutational signature classification in the IMvigor210 and UC‐GENOME test cohorts. Supporting Information 7. Figure S6: Kaplan–Meier curves depicting overall survival (OS) in patients stratified by the genomic mutation–based risk score: score < 1 (low risk) versus ≥ 1 (high risk). Supporting Information 8. Figure S7: Differences in clinical characteristics according to the mutation signature subtype and immunotherapy response. Supporting Information 9. Figure S8: Subgroup analysis of clinical characteristics based on the mutational subtype (IMvigor210 cohort). Supporting Information 10. Figure S9: Subgroup analysis of clinical characteristics based on the mutational subtype (UC‐GENOME cohort). Supporting Information 11. Figure S10: Univariate analysis for mutational signature subtype and clinical characteristics across multiple cohorts. Supporting Information 12. Figure S11: Multivariate analysis for mutational s [file HUMU-2026-2797474-s001.zip › Supplementary Figure 8.pdf]

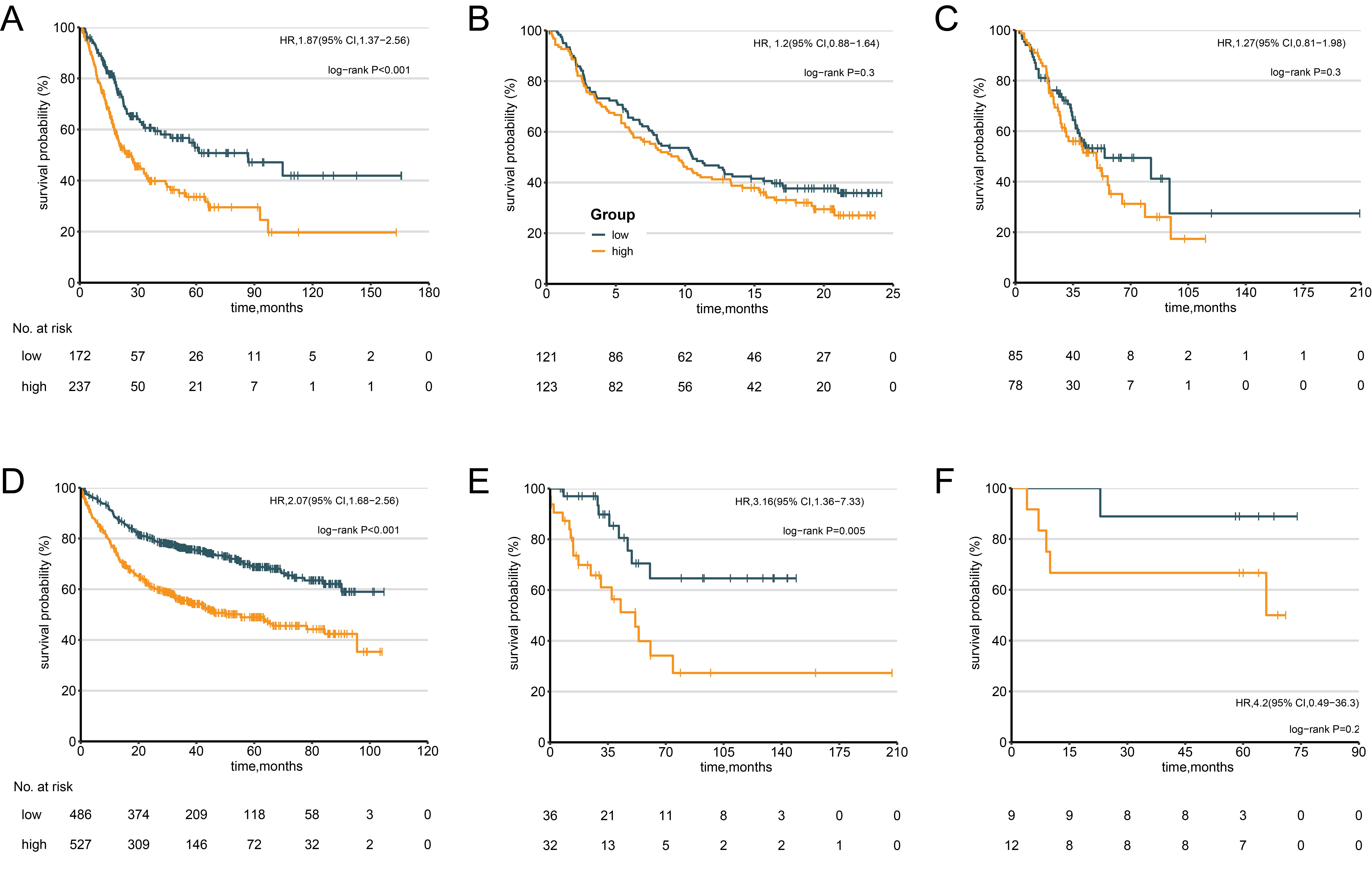

Supplement: Supplementary file 1 — Supporting Information Additional supporting information can be found online in the Supporting Information 1. Supporting Information. Methods S1: Computation and interpretation of cosine similarity. Methods S2: Identification of the mutational subtype of urothelial carcinoma. Methods S3: Identification of the risk score for urothelial carcinoma. Methods S4: Development and external validation of a machine learning–based prediction model. Supporting Information 2. Figure S1: Non‐negative matrix factorization (NMF) of the cosine similarity matrix of mutation signatures from TCGA cohort. Supporting Information 3. Figure S2: Identification and clinical characteristics of mutational signature classification in TCGA training cohort. Supporting Information 4. Figure S3: Clinical characteristics of mutational signature classifications in the MSK2022 test cohort. Supporting Information 5. Figure S4: Clinical characteristics according to the mutational signature classification in the MSK2015 test cohort. Supporting Information 6. Figure S5: Clinical characteristics of mutational signature classification in the IMvigor210 and UC‐GENOME test cohorts. Supporting Information 7. Figure S6: Kaplan–Meier curves depicting overall survival (OS) in patients stratified by the genomic mutation–based risk score: score < 1 (low risk) versus ≥ 1 (high risk). Supporting Information 8. Figure S7: Differences in clinical characteristics according to the mutation signature subtype and immunotherapy response. Supporting Information 9. Figure S8: Subgroup analysis of clinical characteristics based on the mutational subtype (IMvigor210 cohort). Supporting Information 10. Figure S9: Subgroup analysis of clinical characteristics based on the mutational subtype (UC‐GENOME cohort). Supporting Information 11. Figure S10: Univariate analysis for mutational signature subtype and clinical characteristics across multiple cohorts. Supporting Information 12. Figure S11: Multivariate analysis for mutational s [file HUMU-2026-2797474-s001.zip › Supplementary Figure 6.jpg]

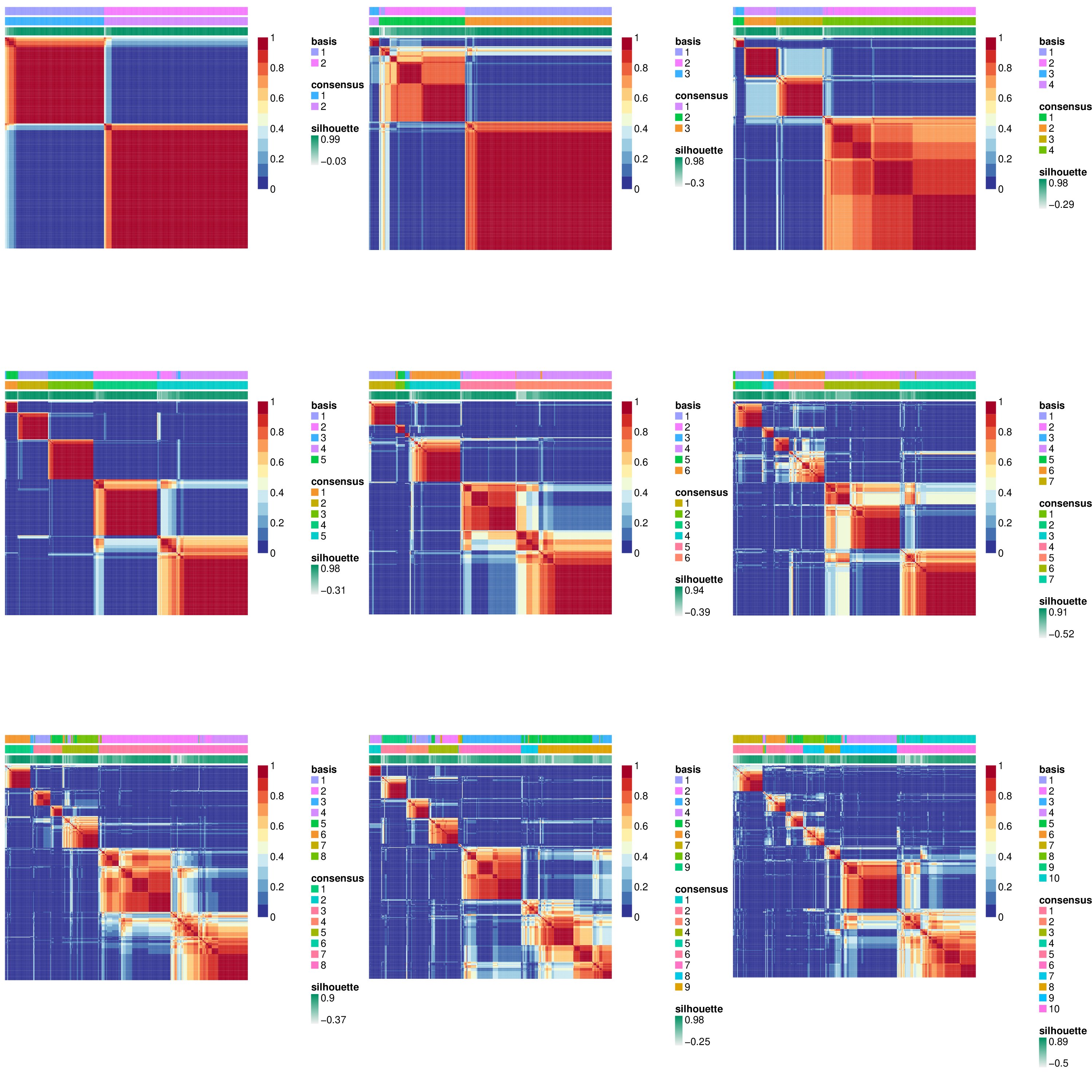

Supplement: Supplementary file 1 — Supporting Information Additional supporting information can be found online in the Supporting Information 1. Supporting Information. Methods S1: Computation and interpretation of cosine similarity. Methods S2: Identification of the mutational subtype of urothelial carcinoma. Methods S3: Identification of the risk score for urothelial carcinoma. Methods S4: Development and external validation of a machine learning–based prediction model. Supporting Information 2. Figure S1: Non‐negative matrix factorization (NMF) of the cosine similarity matrix of mutation signatures from TCGA cohort. Supporting Information 3. Figure S2: Identification and clinical characteristics of mutational signature classification in TCGA training cohort. Supporting Information 4. Figure S3: Clinical characteristics of mutational signature classifications in the MSK2022 test cohort. Supporting Information 5. Figure S4: Clinical characteristics according to the mutational signature classification in the MSK2015 test cohort. Supporting Information 6. Figure S5: Clinical characteristics of mutational signature classification in the IMvigor210 and UC‐GENOME test cohorts. Supporting Information 7. Figure S6: Kaplan–Meier curves depicting overall survival (OS) in patients stratified by the genomic mutation–based risk score: score < 1 (low risk) versus ≥ 1 (high risk). Supporting Information 8. Figure S7: Differences in clinical characteristics according to the mutation signature subtype and immunotherapy response. Supporting Information 9. Figure S8: Subgroup analysis of clinical characteristics based on the mutational subtype (IMvigor210 cohort). Supporting Information 10. Figure S9: Subgroup analysis of clinical characteristics based on the mutational subtype (UC‐GENOME cohort). Supporting Information 11. Figure S10: Univariate analysis for mutational signature subtype and clinical characteristics across multiple cohorts. Supporting Information 12. Figure S11: Multivariate analysis for mutational s [file HUMU-2026-2797474-s001.zip › Supplementary Figure 1.jpg]

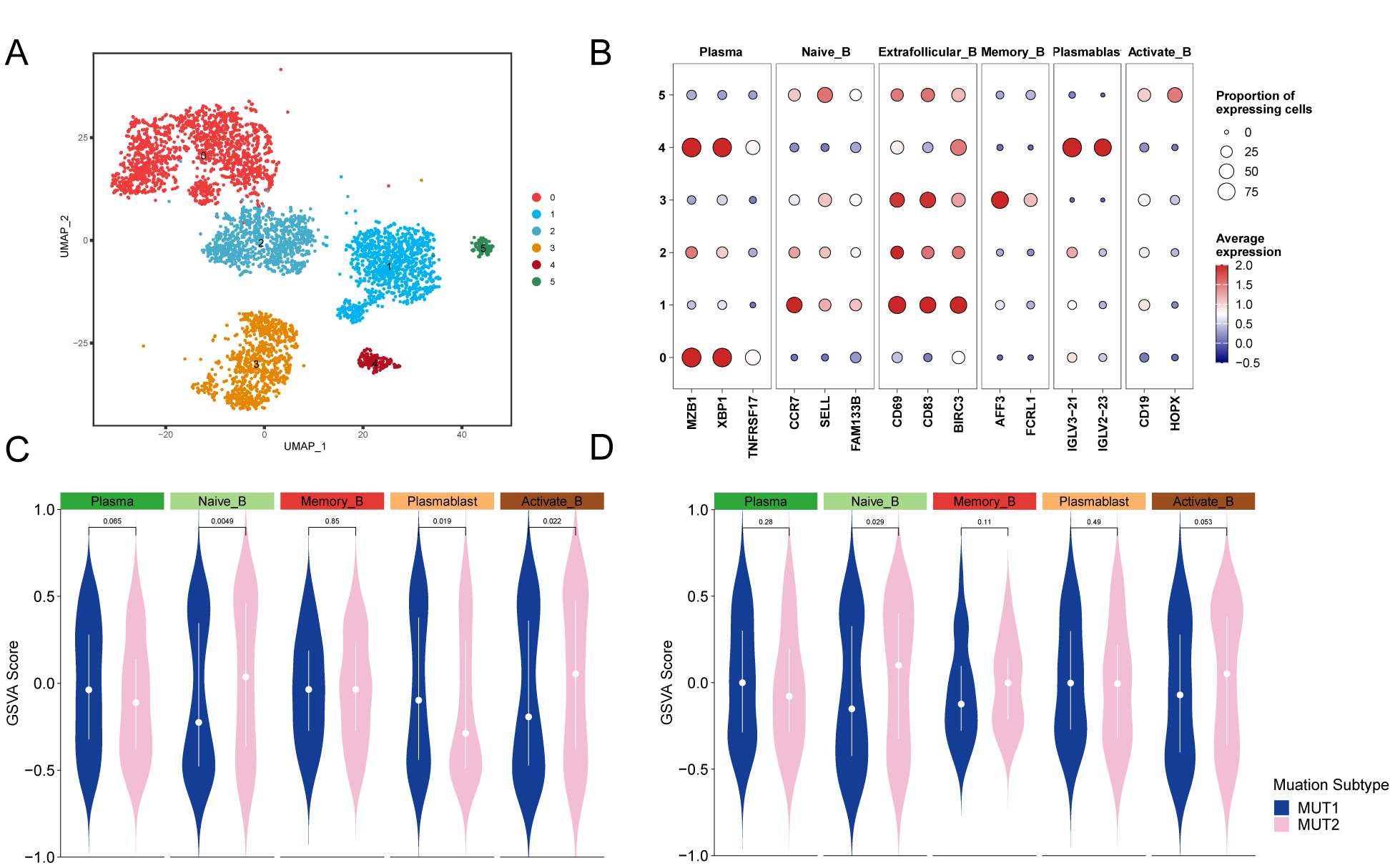

Supplement: Supplementary file 1 — Supporting Information Additional supporting information can be found online in the Supporting Information 1. Supporting Information. Methods S1: Computation and interpretation of cosine similarity. Methods S2: Identification of the mutational subtype of urothelial carcinoma. Methods S3: Identification of the risk score for urothelial carcinoma. Methods S4: Development and external validation of a machine learning–based prediction model. Supporting Information 2. Figure S1: Non‐negative matrix factorization (NMF) of the cosine similarity matrix of mutation signatures from TCGA cohort. Supporting Information 3. Figure S2: Identification and clinical characteristics of mutational signature classification in TCGA training cohort. Supporting Information 4. Figure S3: Clinical characteristics of mutational signature classifications in the MSK2022 test cohort. Supporting Information 5. Figure S4: Clinical characteristics according to the mutational signature classification in the MSK2015 test cohort. Supporting Information 6. Figure S5: Clinical characteristics of mutational signature classification in the IMvigor210 and UC‐GENOME test cohorts. Supporting Information 7. Figure S6: Kaplan–Meier curves depicting overall survival (OS) in patients stratified by the genomic mutation–based risk score: score < 1 (low risk) versus ≥ 1 (high risk). Supporting Information 8. Figure S7: Differences in clinical characteristics according to the mutation signature subtype and immunotherapy response. Supporting Information 9. Figure S8: Subgroup analysis of clinical characteristics based on the mutational subtype (IMvigor210 cohort). Supporting Information 10. Figure S9: Subgroup analysis of clinical characteristics based on the mutational subtype (UC‐GENOME cohort). Supporting Information 11. Figure S10: Univariate analysis for mutational signature subtype and clinical characteristics across multiple cohorts. Supporting Information 12. Figure S11: Multivariate analysis for mutational s [file HUMU-2026-2797474-s001.zip › Supplementary Figure 14.tif]

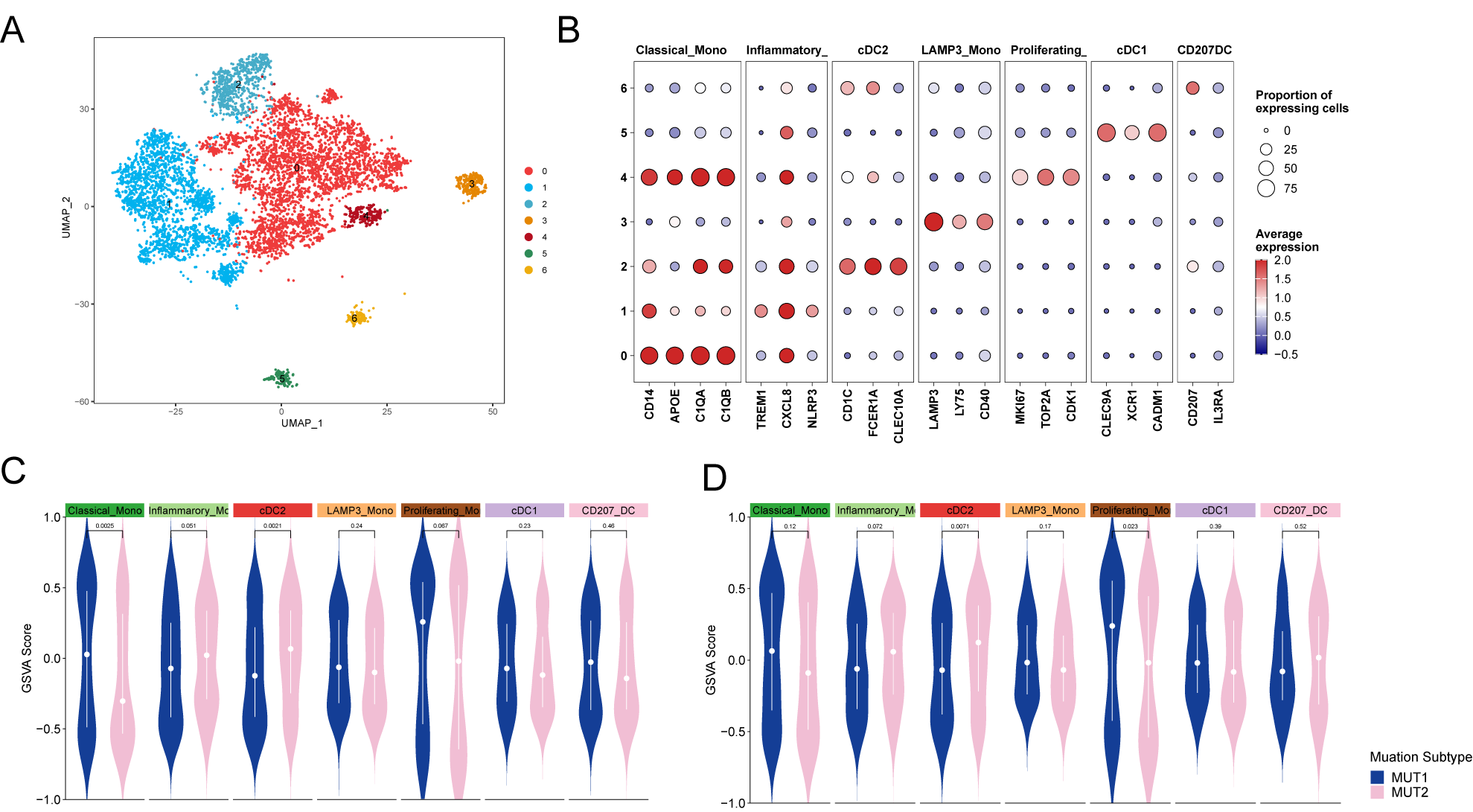

Supplement: Supplementary file 1 — Supporting Information Additional supporting information can be found online in the Supporting Information 1. Supporting Information. Methods S1: Computation and interpretation of cosine similarity. Methods S2: Identification of the mutational subtype of urothelial carcinoma. Methods S3: Identification of the risk score for urothelial carcinoma. Methods S4: Development and external validation of a machine learning–based prediction model. Supporting Information 2. Figure S1: Non‐negative matrix factorization (NMF) of the cosine similarity matrix of mutation signatures from TCGA cohort. Supporting Information 3. Figure S2: Identification and clinical characteristics of mutational signature classification in TCGA training cohort. Supporting Information 4. Figure S3: Clinical characteristics of mutational signature classifications in the MSK2022 test cohort. Supporting Information 5. Figure S4: Clinical characteristics according to the mutational signature classification in the MSK2015 test cohort. Supporting Information 6. Figure S5: Clinical characteristics of mutational signature classification in the IMvigor210 and UC‐GENOME test cohorts. Supporting Information 7. Figure S6: Kaplan–Meier curves depicting overall survival (OS) in patients stratified by the genomic mutation–based risk score: score < 1 (low risk) versus ≥ 1 (high risk). Supporting Information 8. Figure S7: Differences in clinical characteristics according to the mutation signature subtype and immunotherapy response. Supporting Information 9. Figure S8: Subgroup analysis of clinical characteristics based on the mutational subtype (IMvigor210 cohort). Supporting Information 10. Figure S9: Subgroup analysis of clinical characteristics based on the mutational subtype (UC‐GENOME cohort). Supporting Information 11. Figure S10: Univariate analysis for mutational signature subtype and clinical characteristics across multiple cohorts. Supporting Information 12. Figure S11: Multivariate analysis for mutational s [file HUMU-2026-2797474-s001.zip › Supplementary Figure 13.tif]

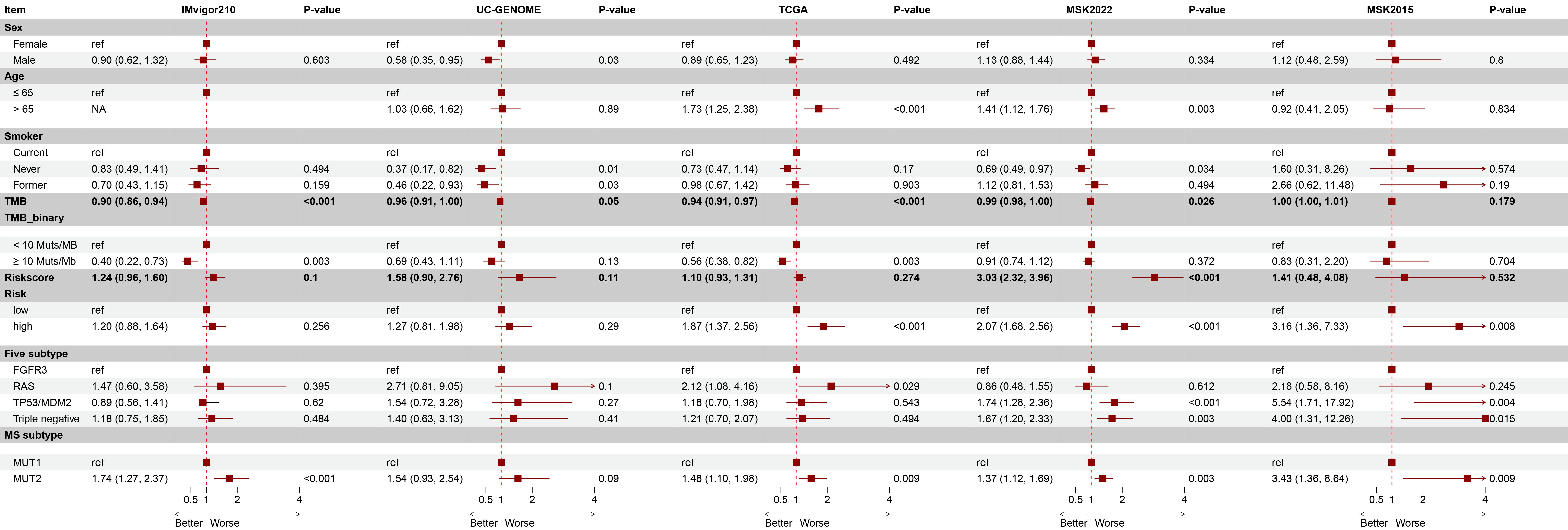

Supplement: Supplementary file 1 — Supporting Information Additional supporting information can be found online in the Supporting Information 1. Supporting Information. Methods S1: Computation and interpretation of cosine similarity. Methods S2: Identification of the mutational subtype of urothelial carcinoma. Methods S3: Identification of the risk score for urothelial carcinoma. Methods S4: Development and external validation of a machine learning–based prediction model. Supporting Information 2. Figure S1: Non‐negative matrix factorization (NMF) of the cosine similarity matrix of mutation signatures from TCGA cohort. Supporting Information 3. Figure S2: Identification and clinical characteristics of mutational signature classification in TCGA training cohort. Supporting Information 4. Figure S3: Clinical characteristics of mutational signature classifications in the MSK2022 test cohort. Supporting Information 5. Figure S4: Clinical characteristics according to the mutational signature classification in the MSK2015 test cohort. Supporting Information 6. Figure S5: Clinical characteristics of mutational signature classification in the IMvigor210 and UC‐GENOME test cohorts. Supporting Information 7. Figure S6: Kaplan–Meier curves depicting overall survival (OS) in patients stratified by the genomic mutation–based risk score: score < 1 (low risk) versus ≥ 1 (high risk). Supporting Information 8. Figure S7: Differences in clinical characteristics according to the mutation signature subtype and immunotherapy response. Supporting Information 9. Figure S8: Subgroup analysis of clinical characteristics based on the mutational subtype (IMvigor210 cohort). Supporting Information 10. Figure S9: Subgroup analysis of clinical characteristics based on the mutational subtype (UC‐GENOME cohort). Supporting Information 11. Figure S10: Univariate analysis for mutational signature subtype and clinical characteristics across multiple cohorts. Supporting Information 12. Figure S11: Multivariate analysis for mutational s [file HUMU-2026-2797474-s001.zip › Supplementary Figure 10.jpg]

**a**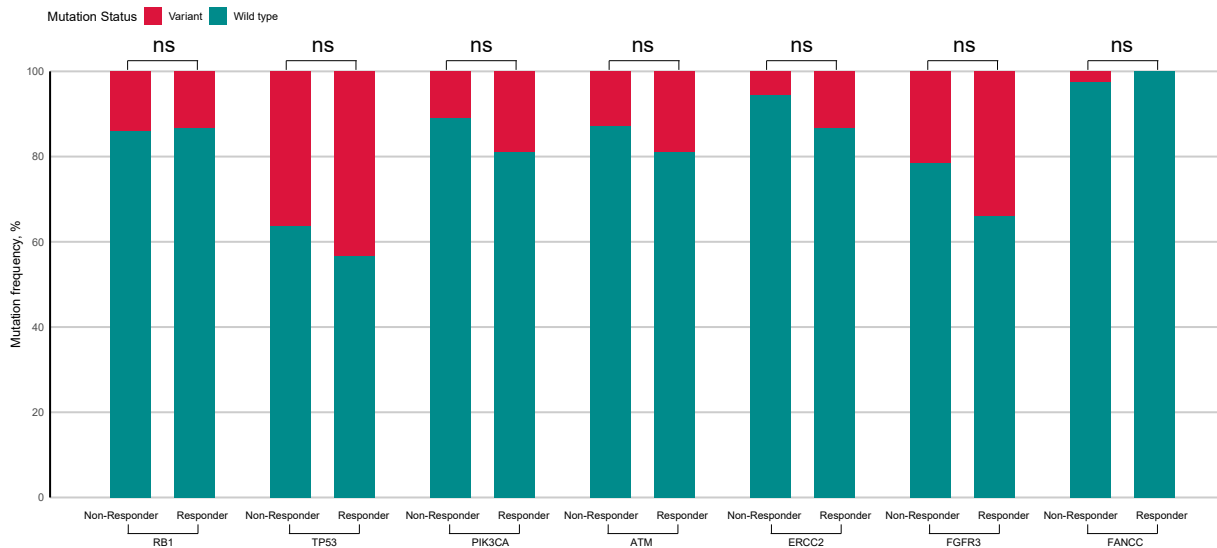**b**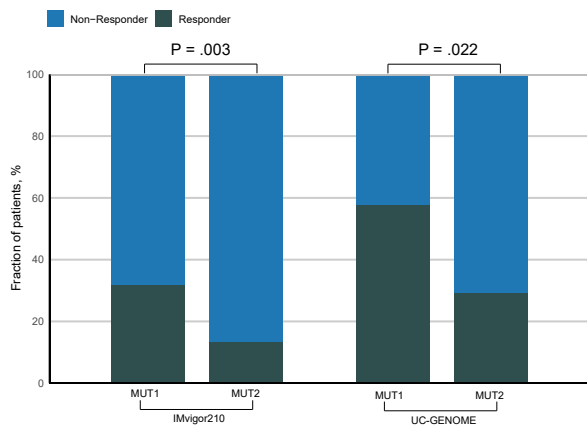**c**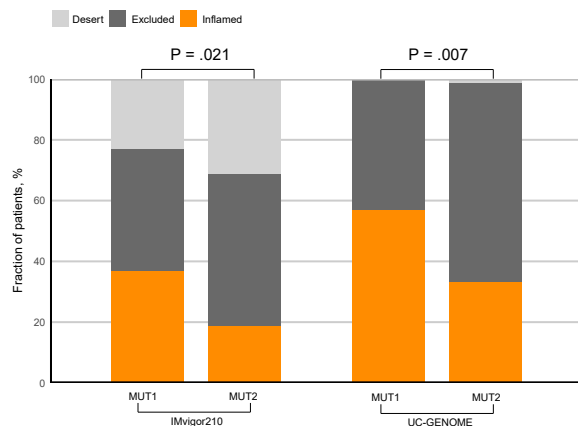**d**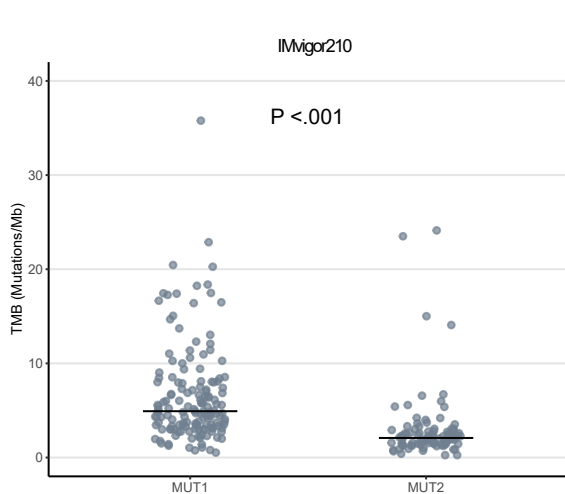**e**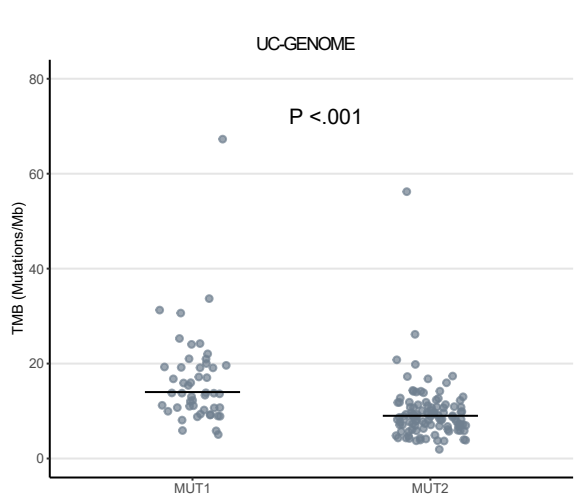

Supplement: Supplementary file 1 — Supporting Information Additional supporting information can be found online in the Supporting Information 1. Supporting Information. Methods S1: Computation and interpretation of cosine similarity. Methods S2: Identification of the mutational subtype of urothelial carcinoma. Methods S3: Identification of the risk score for urothelial carcinoma. Methods S4: Development and external validation of a machine learning–based prediction model. Supporting Information 2. Figure S1: Non‐negative matrix factorization (NMF) of the cosine similarity matrix of mutation signatures from TCGA cohort. Supporting Information 3. Figure S2: Identification and clinical characteristics of mutational signature classification in TCGA training cohort. Supporting Information 4. Figure S3: Clinical characteristics of mutational signature classifications in the MSK2022 test cohort. Supporting Information 5. Figure S4: Clinical characteristics according to the mutational signature classification in the MSK2015 test cohort. Supporting Information 6. Figure S5: Clinical characteristics of mutational signature classification in the IMvigor210 and UC‐GENOME test cohorts. Supporting Information 7. Figure S6: Kaplan–Meier curves depicting overall survival (OS) in patients stratified by the genomic mutation–based risk score: score < 1 (low risk) versus ≥ 1 (high risk). Supporting Information 8. Figure S7: Differences in clinical characteristics according to the mutation signature subtype and immunotherapy response. Supporting Information 9. Figure S8: Subgroup analysis of clinical characteristics based on the mutational subtype (IMvigor210 cohort). Supporting Information 10. Figure S9: Subgroup analysis of clinical characteristics based on the mutational subtype (UC‐GENOME cohort). Supporting Information 11. Figure S10: Univariate analysis for mutational signature subtype and clinical characteristics across multiple cohorts. Supporting Information 12. Figure S11: Multivariate analysis for mutational s [file HUMU-2026-2797474-s001.zip › Supplementary Figure 7.pdf]

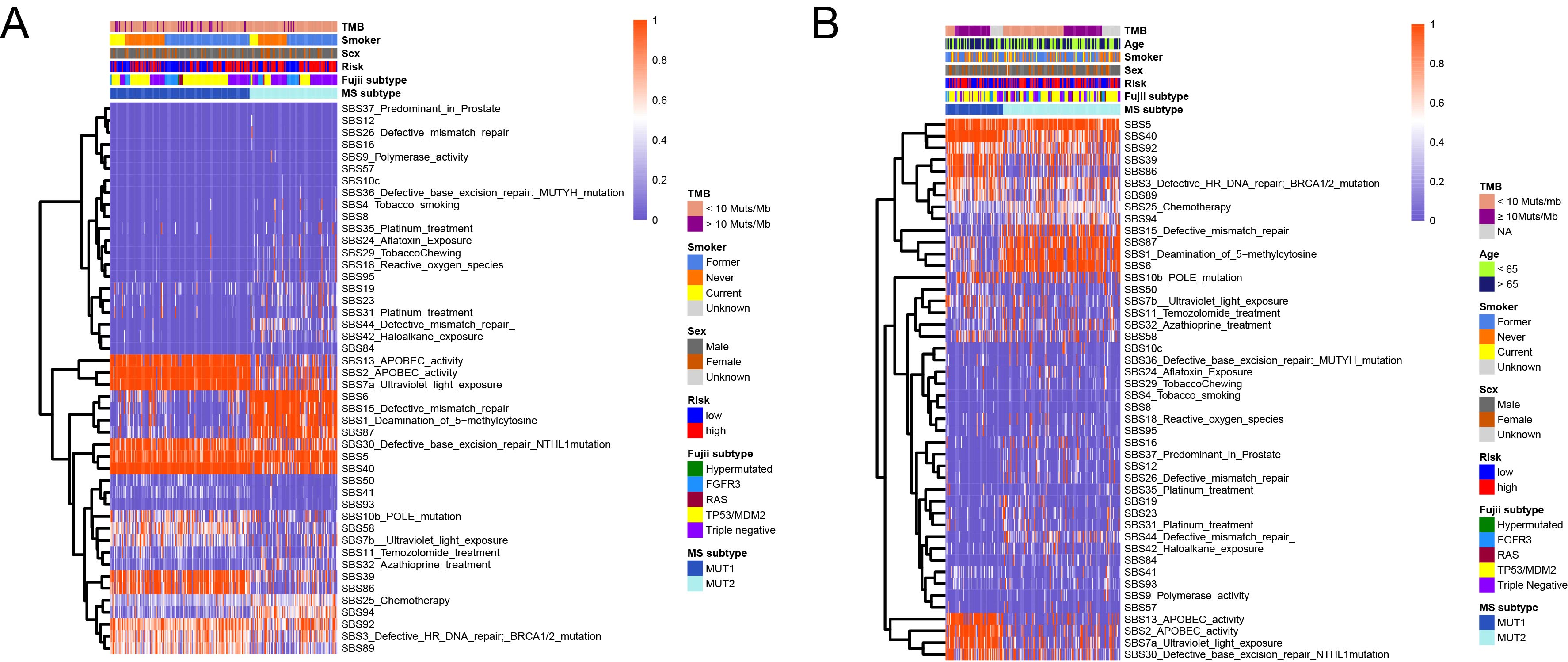

Supplement: Supplementary file 1 — Supporting Information Additional supporting information can be found online in the Supporting Information 1. Supporting Information. Methods S1: Computation and interpretation of cosine similarity. Methods S2: Identification of the mutational subtype of urothelial carcinoma. Methods S3: Identification of the risk score for urothelial carcinoma. Methods S4: Development and external validation of a machine learning–based prediction model. Supporting Information 2. Figure S1: Non‐negative matrix factorization (NMF) of the cosine similarity matrix of mutation signatures from TCGA cohort. Supporting Information 3. Figure S2: Identification and clinical characteristics of mutational signature classification in TCGA training cohort. Supporting Information 4. Figure S3: Clinical characteristics of mutational signature classifications in the MSK2022 test cohort. Supporting Information 5. Figure S4: Clinical characteristics according to the mutational signature classification in the MSK2015 test cohort. Supporting Information 6. Figure S5: Clinical characteristics of mutational signature classification in the IMvigor210 and UC‐GENOME test cohorts. Supporting Information 7. Figure S6: Kaplan–Meier curves depicting overall survival (OS) in patients stratified by the genomic mutation–based risk score: score < 1 (low risk) versus ≥ 1 (high risk). Supporting Information 8. Figure S7: Differences in clinical characteristics according to the mutation signature subtype and immunotherapy response. Supporting Information 9. Figure S8: Subgroup analysis of clinical characteristics based on the mutational subtype (IMvigor210 cohort). Supporting Information 10. Figure S9: Subgroup analysis of clinical characteristics based on the mutational subtype (UC‐GENOME cohort). Supporting Information 11. Figure S10: Univariate analysis for mutational signature subtype and clinical characteristics across multiple cohorts. Supporting Information 12. Figure S11: Multivariate analysis for mutational s [file HUMU-2026-2797474-s001.zip › Supplementary Figure 5.jpg]

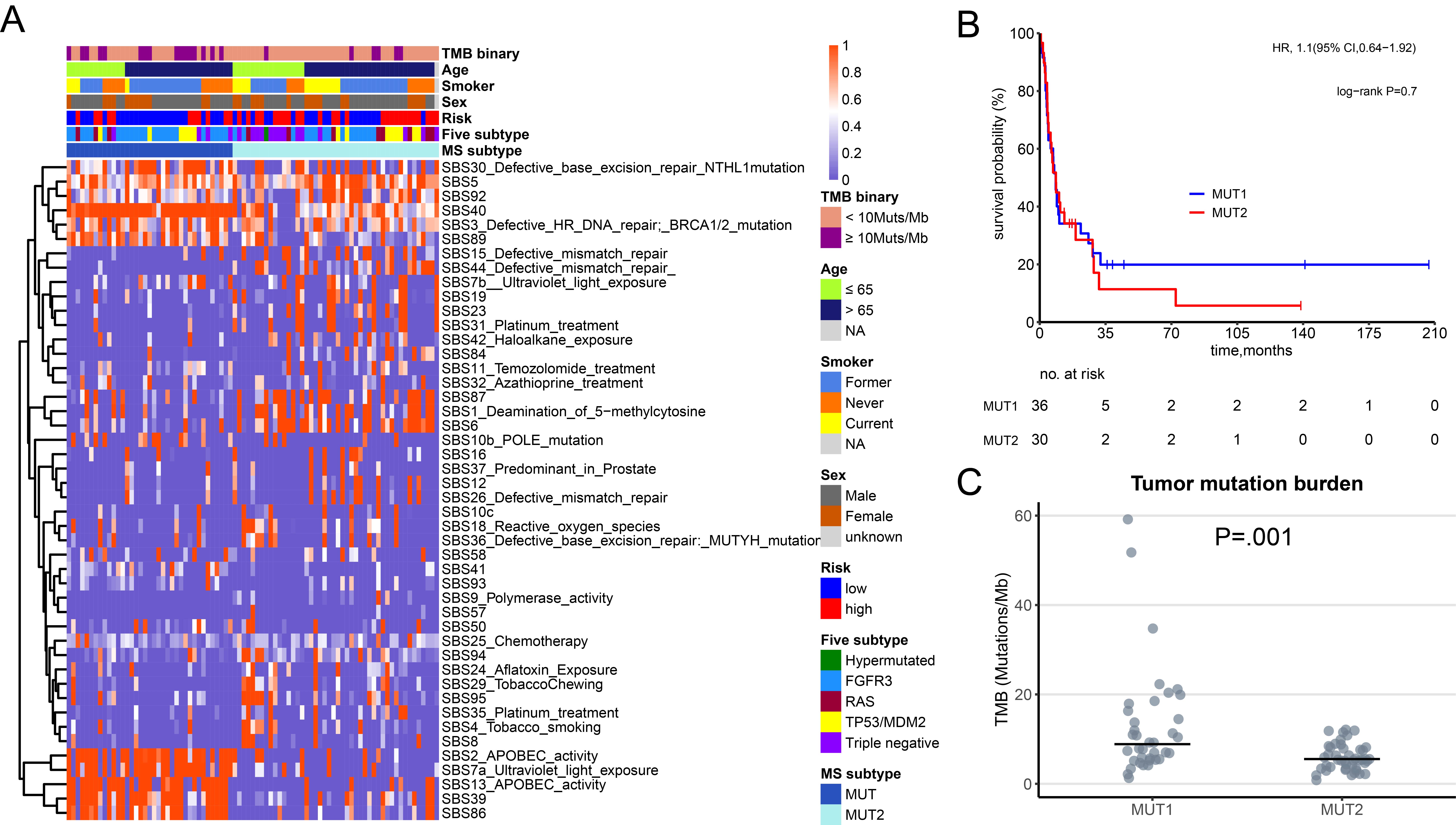

Supplement: Supplementary file 1 — Supporting Information Additional supporting information can be found online in the Supporting Information 1. Supporting Information. Methods S1: Computation and interpretation of cosine similarity. Methods S2: Identification of the mutational subtype of urothelial carcinoma. Methods S3: Identification of the risk score for urothelial carcinoma. Methods S4: Development and external validation of a machine learning–based prediction model. Supporting Information 2. Figure S1: Non‐negative matrix factorization (NMF) of the cosine similarity matrix of mutation signatures from TCGA cohort. Supporting Information 3. Figure S2: Identification and clinical characteristics of mutational signature classification in TCGA training cohort. Supporting Information 4. Figure S3: Clinical characteristics of mutational signature classifications in the MSK2022 test cohort. Supporting Information 5. Figure S4: Clinical characteristics according to the mutational signature classification in the MSK2015 test cohort. Supporting Information 6. Figure S5: Clinical characteristics of mutational signature classification in the IMvigor210 and UC‐GENOME test cohorts. Supporting Information 7. Figure S6: Kaplan–Meier curves depicting overall survival (OS) in patients stratified by the genomic mutation–based risk score: score < 1 (low risk) versus ≥ 1 (high risk). Supporting Information 8. Figure S7: Differences in clinical characteristics according to the mutation signature subtype and immunotherapy response. Supporting Information 9. Figure S8: Subgroup analysis of clinical characteristics based on the mutational subtype (IMvigor210 cohort). Supporting Information 10. Figure S9: Subgroup analysis of clinical characteristics based on the mutational subtype (UC‐GENOME cohort). Supporting Information 11. Figure S10: Univariate analysis for mutational signature subtype and clinical characteristics across multiple cohorts. Supporting Information 12. Figure S11: Multivariate analysis for mutational s [file HUMU-2026-2797474-s001.zip › Supplementary Figure 4.jpg]

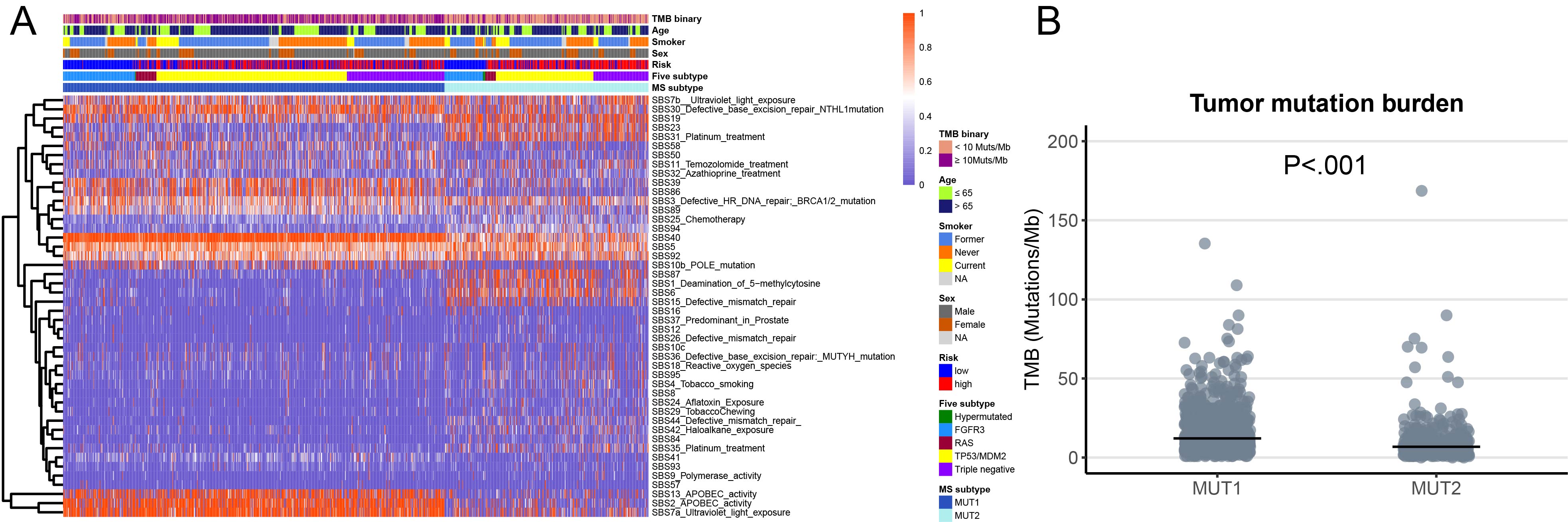

Supplement: Supplementary file 1 — Supporting Information Additional supporting information can be found online in the Supporting Information 1. Supporting Information. Methods S1: Computation and interpretation of cosine similarity. Methods S2: Identification of the mutational subtype of urothelial carcinoma. Methods S3: Identification of the risk score for urothelial carcinoma. Methods S4: Development and external validation of a machine learning–based prediction model. Supporting Information 2. Figure S1: Non‐negative matrix factorization (NMF) of the cosine similarity matrix of mutation signatures from TCGA cohort. Supporting Information 3. Figure S2: Identification and clinical characteristics of mutational signature classification in TCGA training cohort. Supporting Information 4. Figure S3: Clinical characteristics of mutational signature classifications in the MSK2022 test cohort. Supporting Information 5. Figure S4: Clinical characteristics according to the mutational signature classification in the MSK2015 test cohort. Supporting Information 6. Figure S5: Clinical characteristics of mutational signature classification in the IMvigor210 and UC‐GENOME test cohorts. Supporting Information 7. Figure S6: Kaplan–Meier curves depicting overall survival (OS) in patients stratified by the genomic mutation–based risk score: score < 1 (low risk) versus ≥ 1 (high risk). Supporting Information 8. Figure S7: Differences in clinical characteristics according to the mutation signature subtype and immunotherapy response. Supporting Information 9. Figure S8: Subgroup analysis of clinical characteristics based on the mutational subtype (IMvigor210 cohort). Supporting Information 10. Figure S9: Subgroup analysis of clinical characteristics based on the mutational subtype (UC‐GENOME cohort). Supporting Information 11. Figure S10: Univariate analysis for mutational signature subtype and clinical characteristics across multiple cohorts. Supporting Information 12. Figure S11: Multivariate analysis for mutational s [file HUMU-2026-2797474-s001.zip › Supplementary Figure 3.jpg]

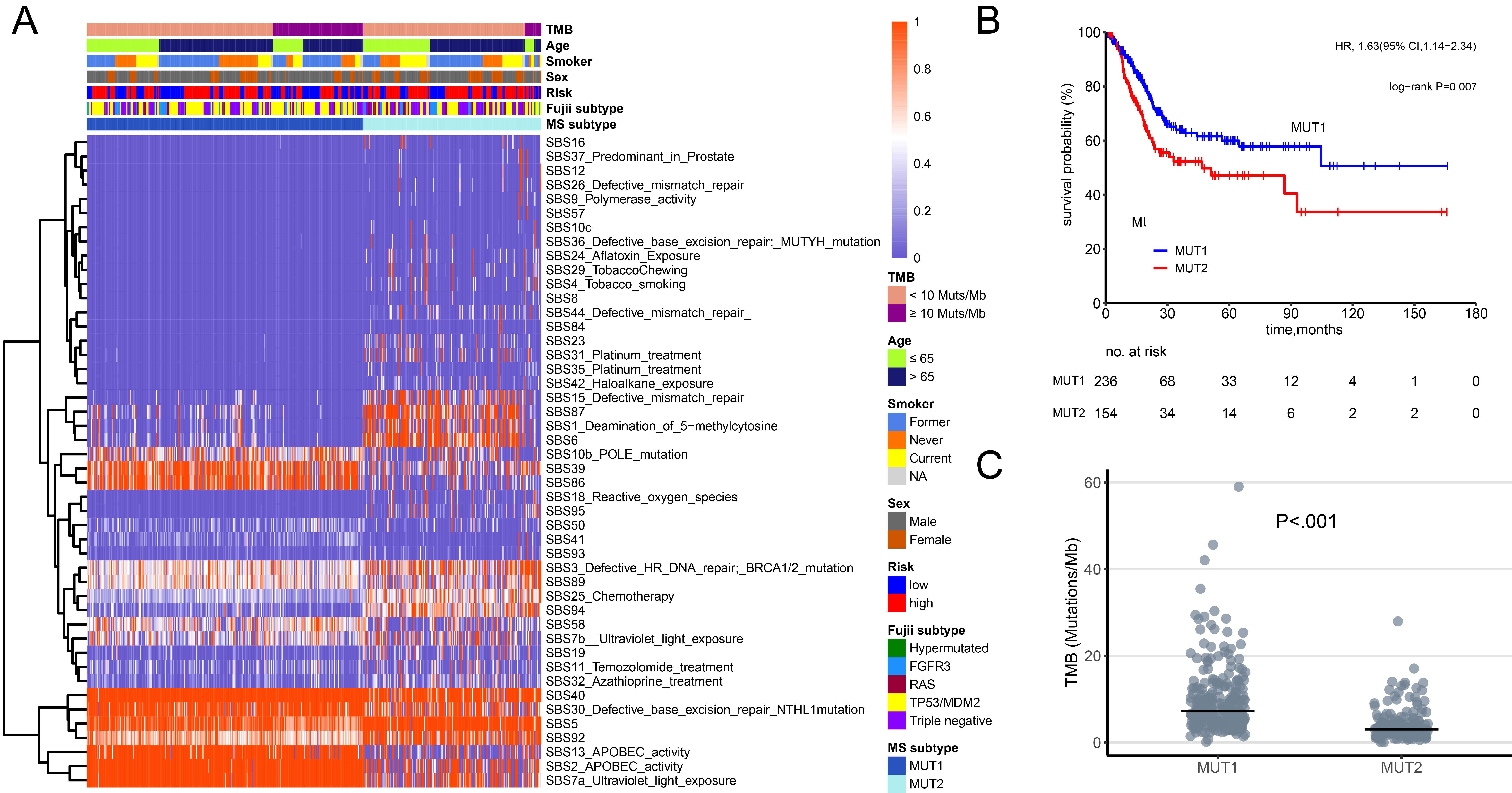

Supplement: Supplementary file 1 — Supporting Information Additional supporting information can be found online in the Supporting Information 1. Supporting Information. Methods S1: Computation and interpretation of cosine similarity. Methods S2: Identification of the mutational subtype of urothelial carcinoma. Methods S3: Identification of the risk score for urothelial carcinoma. Methods S4: Development and external validation of a machine learning–based prediction model. Supporting Information 2. Figure S1: Non‐negative matrix factorization (NMF) of the cosine similarity matrix of mutation signatures from TCGA cohort. Supporting Information 3. Figure S2: Identification and clinical characteristics of mutational signature classification in TCGA training cohort. Supporting Information 4. Figure S3: Clinical characteristics of mutational signature classifications in the MSK2022 test cohort. Supporting Information 5. Figure S4: Clinical characteristics according to the mutational signature classification in the MSK2015 test cohort. Supporting Information 6. Figure S5: Clinical characteristics of mutational signature classification in the IMvigor210 and UC‐GENOME test cohorts. Supporting Information 7. Figure S6: Kaplan–Meier curves depicting overall survival (OS) in patients stratified by the genomic mutation–based risk score: score < 1 (low risk) versus ≥ 1 (high risk). Supporting Information 8. Figure S7: Differences in clinical characteristics according to the mutation signature subtype and immunotherapy response. Supporting Information 9. Figure S8: Subgroup analysis of clinical characteristics based on the mutational subtype (IMvigor210 cohort). Supporting Information 10. Figure S9: Subgroup analysis of clinical characteristics based on the mutational subtype (UC‐GENOME cohort). Supporting Information 11. Figure S10: Univariate analysis for mutational signature subtype and clinical characteristics across multiple cohorts. Supporting Information 12. Figure S11: Multivariate analysis for mutational s [file HUMU-2026-2797474-s001.zip › Supplementary Figure 2.jpg]

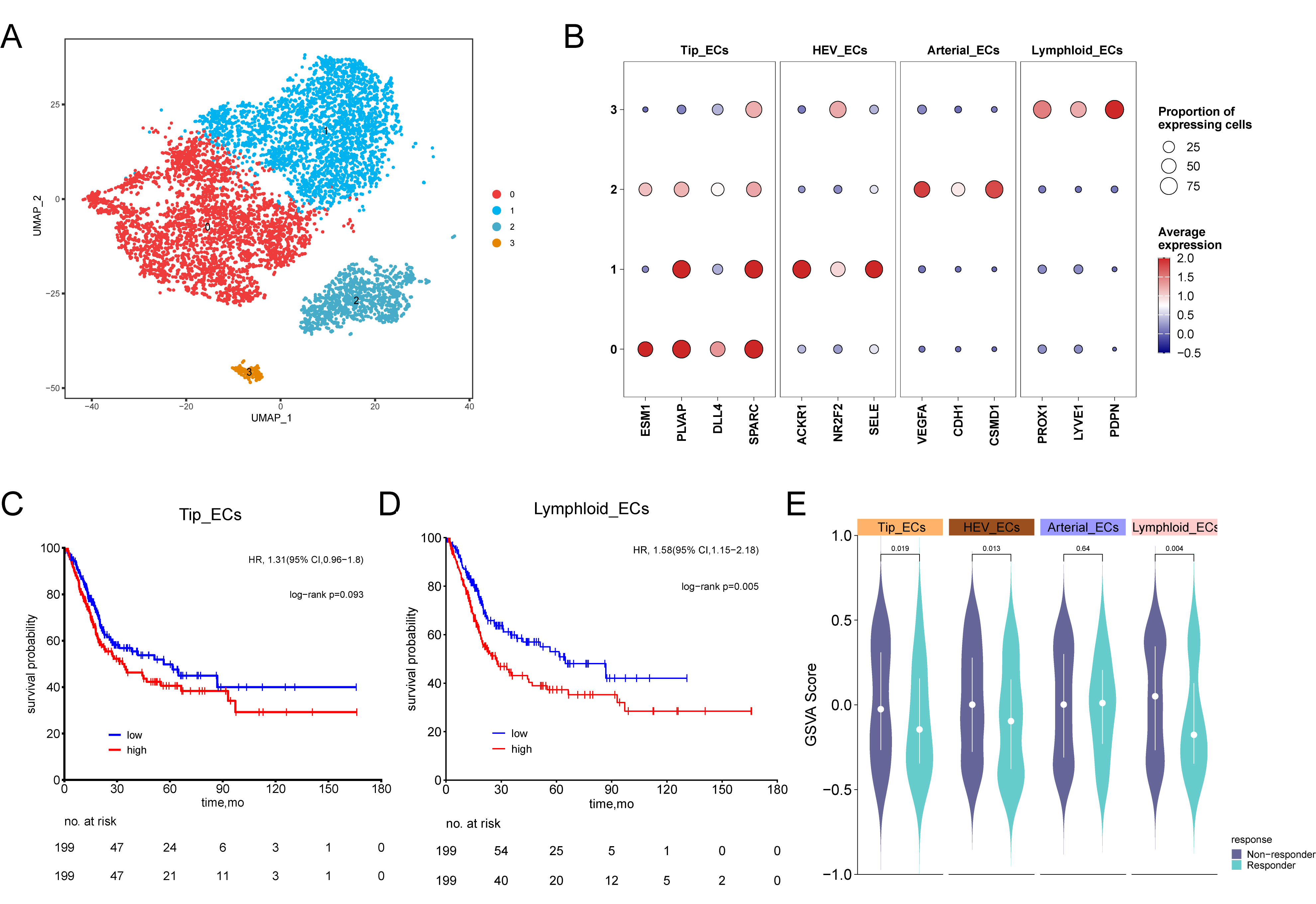

Supplement: Supplementary file 1 — Supporting Information Additional supporting information can be found online in the Supporting Information 1. Supporting Information. Methods S1: Computation and interpretation of cosine similarity. Methods S2: Identification of the mutational subtype of urothelial carcinoma. Methods S3: Identification of the risk score for urothelial carcinoma. Methods S4: Development and external validation of a machine learning–based prediction model. Supporting Information 2. Figure S1: Non‐negative matrix factorization (NMF) of the cosine similarity matrix of mutation signatures from TCGA cohort. Supporting Information 3. Figure S2: Identification and clinical characteristics of mutational signature classification in TCGA training cohort. Supporting Information 4. Figure S3: Clinical characteristics of mutational signature classifications in the MSK2022 test cohort. Supporting Information 5. Figure S4: Clinical characteristics according to the mutational signature classification in the MSK2015 test cohort. Supporting Information 6. Figure S5: Clinical characteristics of mutational signature classification in the IMvigor210 and UC‐GENOME test cohorts. Supporting Information 7. Figure S6: Kaplan–Meier curves depicting overall survival (OS) in patients stratified by the genomic mutation–based risk score: score < 1 (low risk) versus ≥ 1 (high risk). Supporting Information 8. Figure S7: Differences in clinical characteristics according to the mutation signature subtype and immunotherapy response. Supporting Information 9. Figure S8: Subgroup analysis of clinical characteristics based on the mutational subtype (IMvigor210 cohort). Supporting Information 10. Figure S9: Subgroup analysis of clinical characteristics based on the mutational subtype (UC‐GENOME cohort). Supporting Information 11. Figure S10: Univariate analysis for mutational signature subtype and clinical characteristics across multiple cohorts. Supporting Information 12. Figure S11: Multivariate analysis for mutational s [file HUMU-2026-2797474-s001.zip › Supplementary Figure 12.tif]
